# Light-enabled intramolecular [2 + 2] cycloaddition via photoactivation of simple alkenylboronic esters

**DOI:** 10.3762/bjoc.21.69

**Published:** 2025-04-30

**Authors:** Lewis McGhie, Hannah M Kortman, Jenna Rumpf, Peter H Seeberger, John J Molloy

**Affiliations:** 1 Biomolecular Systems Department, Max Planck Institute of Colloids and Interfaces, Am Mühlenberg 1, 14476 Potsdam, Germany; 2 Department of Chemistry and Biochemistry, Freie Universität Berlin, Arnimallee 22, 14195 Berlin, Germany

**Keywords:** boron, catalysis, [2 + 2] cycloaddition, energy transfer, photochemistry

## Abstract

The photoactivation of organic molecules via energy transfer (EnT) catalysis is often limited to conjugated systems that have low-energy triplet excited states, with simple alkenes remaining an intractable challenge. The ability to address this limitation, using high energy sensitizers, would represent an attractive platform for future reaction design. Here, we disclose the photoactivation of simple alkenylboronic esters established using alkene scrambling as a rapid reaction probe to identify a suitable catalyst and boron motif. Cyclic voltammetry, UV–vis analysis, and control reactions support sensitization, enabling an intramolecular [2 + 2] cycloaddition to be realized accessing 3D bicyclic fragments containing a boron handle.

## Introduction

The strategic use of a photon to activate organic molecules has had a profound impact on contemporary synthesis, enabling the practitioner to strategically construct molecules that are higher in energy from simple feedstock commodities [1–5]. Indeed, the unique ability to access high energy intermediates leveraging light, has facilitated landmark organic transformations, such as the venerable Paternò–Büchi [6–8], Norrish–Yang [9–11], and enone–alkene cycloadditions [12–14], that proceed via the generation of a singlet or triplet diradical through the activation of an unsaturated bond [2,14]. While these seminal contributions have enabled the efficient activation of carbonyl and alkene moieties, the inability of most organic molecules to efficiently absorb photons at longer wavelengths often preclude their use in direct excitation strategies, requiring unique experimental set ups and light sources that are prohibitively high in energy for selective reactivity [5].

**Figure 1:**
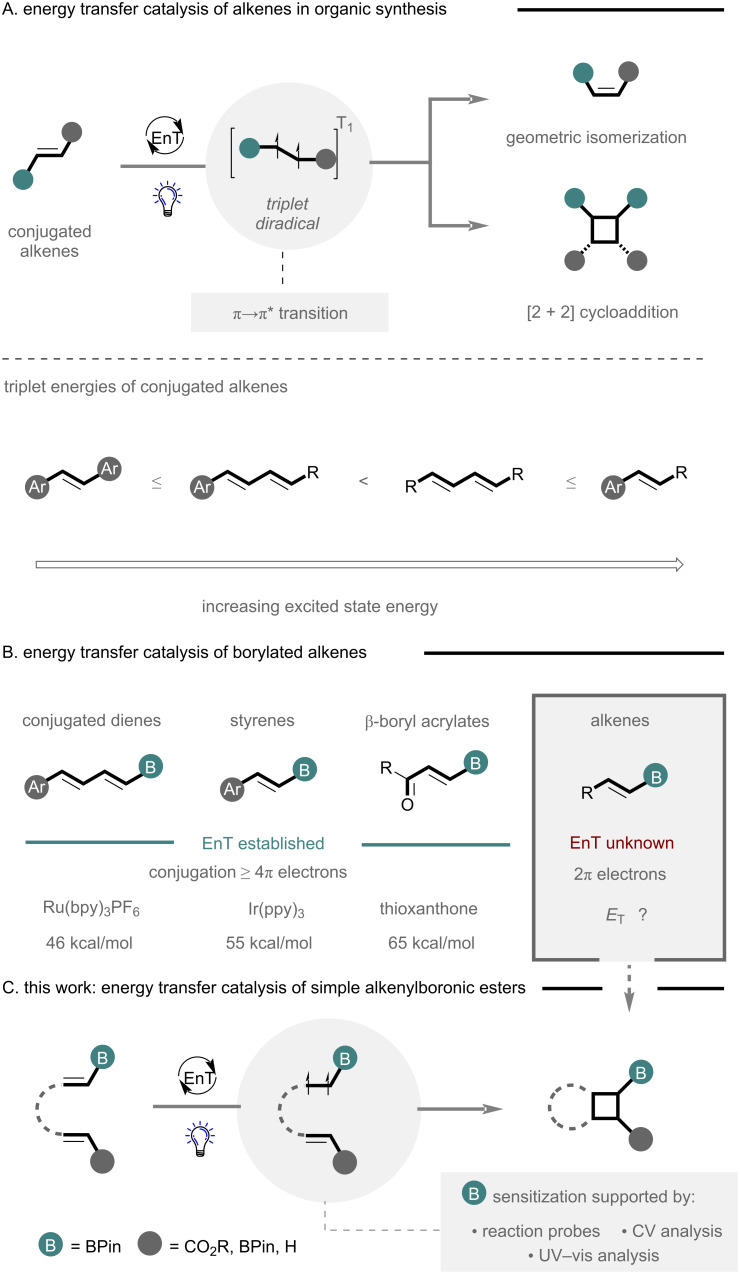
A) Energy transfer catalysis of alkenes in organic synthesis. B) Energy transfer catalysis of conjugated borylated alkenes. C) Energy transfer catalysis of simple alkenylboronic esters.

The inception of energy transfer catalysis (EnT) has expedited discoveries concerning the photoactivation of organic molecules [15–17], enabling direct access to the triplet excited state through the use of a photocatalyst (Figure 1A, top). Pioneering studies have leveraged this platform with great effect, typically invoking π→π* transitions of conjugated alkenes to lower the bond order and generate a triplet diradical, primed for further reactivity. This key intermediate is pivotal in a plenum of synthetic transformations including geometric isomerization of alkenes [18–19], [2 + 2] cycloadditions [20–24], and dearomative [4 + 2] cycloadditions [25–27]. When considering conjugated alkenes, the triplet excited state energy and excited state lifetime are intrinsically linked to the degree of conjugation and substitution of a scaffold (Figure 1A, bottom), with increasing excited state energy moving from stilbenes and conjugated dienes to simple dienes, styrenes and enones (not shown) [28]. Consequently, small truncated chromophores, such as simple alkenes remain an intractable challenge for efficient EnT due to prohibitively high excited state energies and short lifetimes [29]. However, with notable strides in catalyst design, leading to catalysts with high excited state energies [30–33], in combination with concomitant advances in machine learning excited state predictions [34], it is anticipated that perhaps even the most challenging of substrates can be realized in future endeavors.

Given the dexterity of organoboron handles, both as exit vectors for the exploration of chemical space [35–38], and as covalent binders to target biomolecules [39–41], it serves as no great surprise that their reactivity in EnT catalysis has also been intensively pursued (Figure 1B). The controlled geometric isomerization of boron-containing conjugated dienes [42], styrenes [43–45] and β-boryl acrylates [46–47] has been established with great effect, while elegant [2 + 2] cycloaddition strategies to readily translate 2D to 3D chemical space have also been explored [48–52]. Here, efficient excitation, via EnT catalysis, is typically contingent on extended chromophores ≥ 4π electrons, with less conjugated systems requiring more powerful catalysts. A recent elegant example by Masarwa and co-workers demonstrated the efficient sensitization of an alkene-containing four boron substituents using Ir(ppy)_3_ as a suitable sensitizer in the presence of styrene, indicating a prominent role of the adjacent p-orbital [51]. While simple alkenylboronic esters have been employed as triplet diradical quenchers to facilitate the synthesis of boron-containing cyclobutanes [53–55], excitation of these motifs to form a triplet diradical, and its application in a synthetic process, is conspicuously underexplored, presumably due to unreachable excited states and poor lifetimes when limited to a single boron unit.

Herein, we demonstrate the sensitization of alkenylboronates to enable efficient intramolecular [2 + 2] cycloaddition using high energy photosensitizers (Figure 1C). Sensitization was quickly established and explored through the use of alkene scrambling (geometrical isomerization) reaction probes, to identify a suitable catalyst and boron residue, while control reactions and mechanistic studies support the proposed sensitization. The platform enables direct access to mono- and vicinal cyclobutylboronic esters that could be effectively derivatized to demonstrate their potential in synthesis.

## Results and Discussion

To validate the proposed sensitization of alkenylboronic esters, we initiated our study probing the geometric isomerization of easily accessible substrate (*E*)-**1a** under photocatalysis conditions (Table 1). It is pertinent to note, while the core substrate (*E*)-**1a** lacks the necessary non-covalent interactions to achieve directionality (*E*→*Z*), synonymous with conventional photocatalyzed isomerization processes [19], it serves as a rapid reaction probe to support sensitization via the generation of a photostationary state equilibrium. The use of Ir(ppy)_3_ (Table 1, entry 1), an efficient sensitizer for the activation of polyboronated alkenes [51] and β-borylstyrenes [43,48], was ineffective and no reactivity was observed. Implementing a higher energy iridium sensitizer (≈60 kcal/mol) was also unsuccessful (Table 1, entry 2), however, employing thioxanthone (65 kcal/mol) demonstrated efficient activation of the alkene leading to the generation of an 89:11 (*E*/*Z*) mixture of geometrical isomers (Table 1, entry 3). The use of xanthone (74 kcal/mol), a highly powerful organic photocatalyst, enabled enhanced reactivity producing a photostationary state of 73:27 after 16 hours (Table 1, entry 4). Varying solvent had a profound effect on reactivity with more Lewis-basic solvents suppressing reactivity (Table 1, entries 4 and 5 vs entries 6 and 7), indicating p-orbital availability plays a prominent role [56–57]. A control reaction (Table 1, entry 8), in combination with UV–vis absorption studies (see Supporting Information File 1 for full details), demonstrate that catalysis is operational.

**Table 1:** Probing EnT catalysis of alkenylboronic ester **1a** via alkene scrambling.^a^

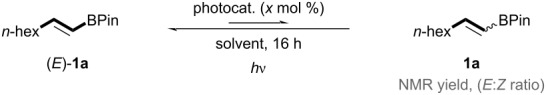						

Entry	Catalyst	*E*_T_ (kcal/mol)^b^	λ (nm)	Solvent	Yield (%)^c^	*E*/*Z*^c^

1	Ir(ppy)_3_	55	450	MeCN	92	>95:5
2	(Ir[dF(CF_3_)ppy]_2_(dtbpy))PF_6_	60	450	MeCN	92	>95:5
3^d^	thioxanthone	65	400	MeCN	81	89:11
4^d^	xanthone	74	365	MeCN	83	73:27
5^d^	xanthone	74	365	DCM	80	75:25
6^d^	xanthone	74	365	THF	86	>95:5
7^d^	xanthone	74	365	DMF	86	>95:5
8	–	–	365	MeCN	92	>95:5

^a^Standard conditions: (*E*)-**1a** (0.1 mmol), cat. (1 mol %), solvent (0.03 M), under light irradiation (1 W), rt, 16 h; ^b^see [28]; ^c^determined by ^1^H NMR spectroscopy against a known internal standard (1,3,5-trimethoxybenzene); ^d^5 mol % catalyst loading.

**Figure 2:**
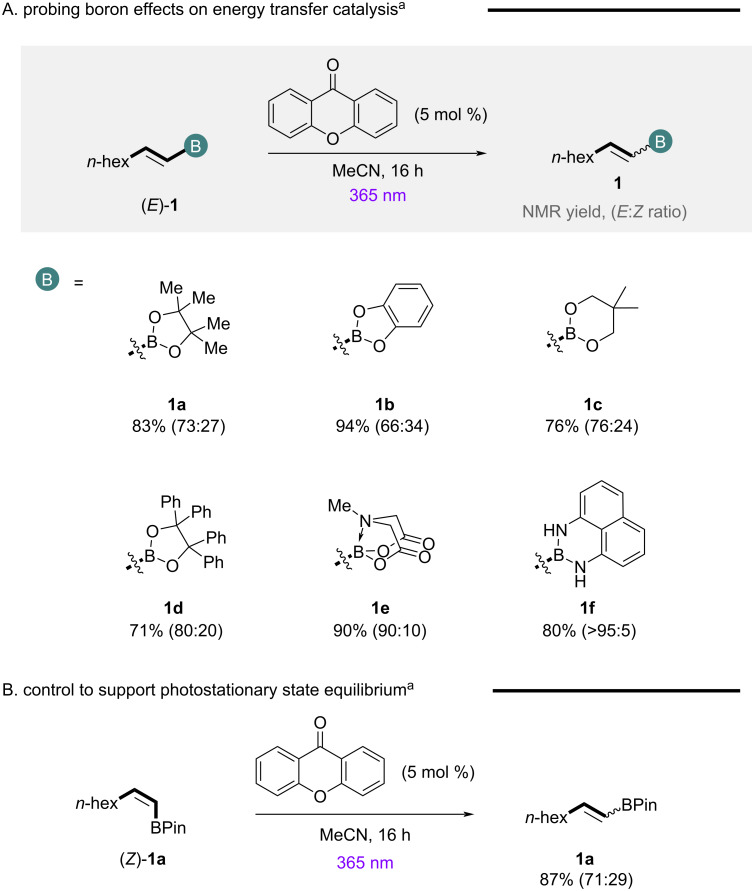
Probing boron effects on reactivity (A) and confirming the generation of a photostationary state equilibrium (B). Standard reaction conditions: (*E*)-**1** (0.1 mmol), xanthone (5 mol %), MeCN (0.03 M), rt, 16 h.

Having established xanthone as a suitable high energy sensitizer for the efficient activation of simple alkenylboronic esters, we next set out to determine the role of the boron unit and hybridization state (Figure 2A). Efficient EnT catalysis could be achieved with neutral trigonal planar systems **1a**–**d**, while BMIDA substrate **1e** was also effective presumably due to the proposed hemilabile nature of the MIDA protecting group in acetonitrile [58]. More electron-donating ligands such as 1,8-diaminonaphthalene (BDAN, **1f**) were detrimental to reactivity leading to substrate degradation. Given the ease of access and enhanced stability of pinacol esters to column chromatography, this motif was advanced for further reaction design. Cyclic voltammetry analysis of **1a** indicates that single-electron-transfer processes with the excited state catalyst are not operational (see Supporting Information File 1 for full details), providing support for an EnT mechanism, while exposing the *Z*-isomer (*Z*)-**1a** to the model reaction conditions, led to the generation of a similar photostationary state equilibrium of isomers, characteristic of an EnT process (Figure 2B) [19].

**Figure 3:**
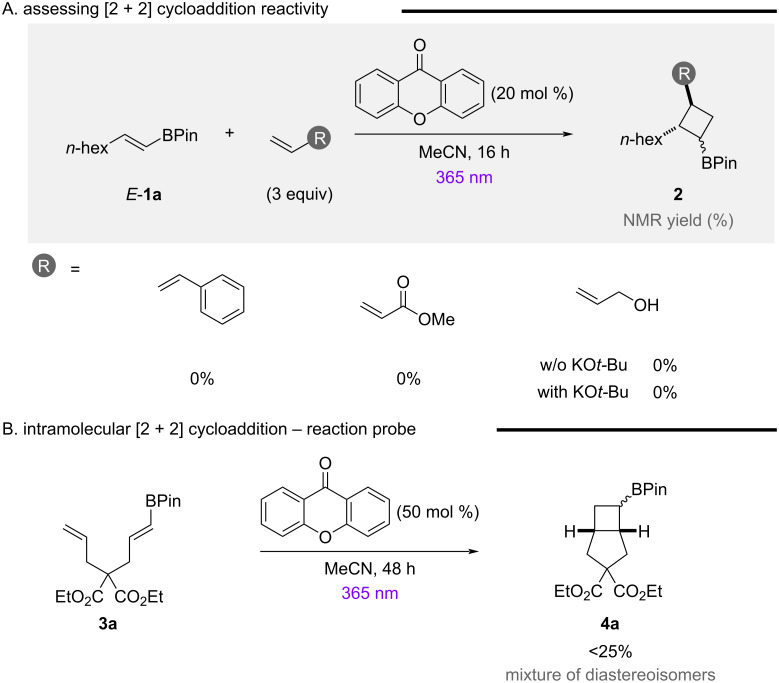
Probing EnT catalysis enabled [2 + 2] cycloaddition of simple alkenylboronic esters.

In a bid to translate sensitization reaction probes to meaningful synthetic transformations, conventional intermolecular [2 + 2] cycloaddition reactions were initially trialled (Figure 3A). Preliminary reactions using styrene or methyl acrylate were unsuccessful, with no [2 + 2] cycloaddition observed, despite the use of higher catalyst loadings. The efficient isomerization of **1a** during these reactions indicated that substrate lifetime for efficient intermolecular reactivity may be problematic. While substrate-tethered reactivity, developed by Brown and co-workers was unsuccessful [49], the strategic incorporation of an alkene tether, bringing both alkenes spatially proximal, facilitated a [2 + 2] cycloaddition to generate bicyclic product **4a** as a 1:1 mixture of diastereoisomers in low yield. This provides an exciting proof of principle that it is possible to efficiently quench the generated triplet diradical in an intramolecular system.

To further assess the properties relevant for intramolecular reactivity, substrate **3b** was designed (see Supporting Information File 1 for full details), given that non-activated α,β-unsaturated systems are also comparatively underexplored via EnT activation (Table 2) [59–60]. As anticipated, guided by alkene scrambling reaction probes (Table 1), iridium sensitizers were unsuccessful (Table 2, entries 1 and 2). Thioxanthone provided the cyclobutyl product **4b** in 10% yield (Table 2, entry 3), indicating catalysis with poor overlap between the catalyst (65 kcal/mol) and substrate. The use of xanthone demonstrated clean translation to product favouring *syn* diastereoselectivity [61–62] (Table 2, entry 4), with additional catalyst increasing yield during the 16 h reaction time (Table 2, entry 5).

**Table 2:** Reaction optimization of intramolecular [2 + 2] cycloaddition.^a^

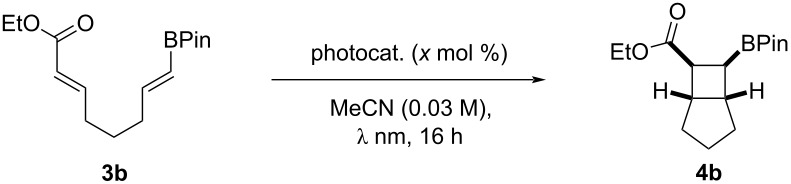						

Entry	Catalyst	mol %	*E*_T_ (kcal/mol)	λ (nm)	Yield (%)^b^	dr^b^

1	Ir(p-CF_3_)_3_	1	56	450	0	–
2	(Ir[dF(CF_3_)ppy]_2_(dtbpy))PF_6_	1	60	450	0	–
3	thioxanthone	5	65	390	10	–
4	xanthone	5	74	365	35	6:1
**5**	**xanthone**	**20**	**74**	**365**	**76**	**5:1**

^a^Standard conditions: **3b** (0.1 mmol), cat. (*x* mol %), MeCN (0.03 M), under light irradiation (1 W), rt, 16 h; ^b^determined by ^1^H NMR spectroscopy against a known internal standard (1,3,5-trimethoxybenzene).

**Scheme 1:**
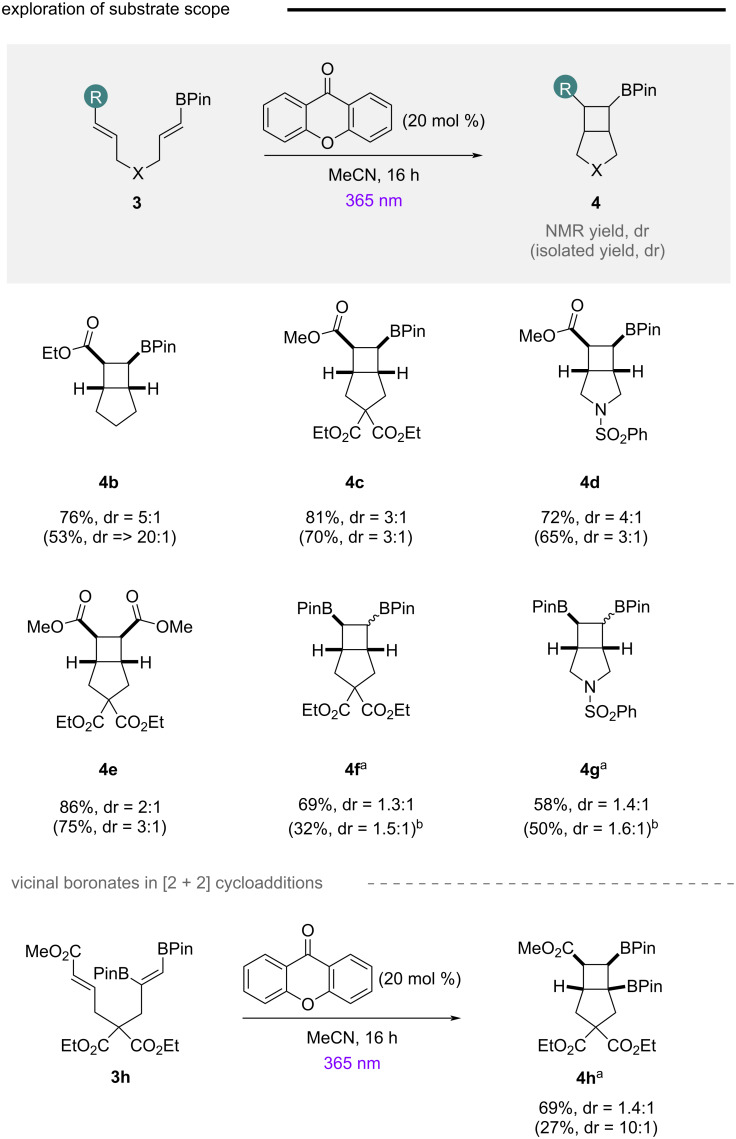
Establishing the substrate scope. Conditions: **3** (1 equiv), xanthone (20 mol %), MeCN (0.03 M), under 365 nm irradiation, rt, 16 h; ^a^xanthone (50 mol %) was used and reactions were run for 48 h; ^b^to aid characterization of isolated material, the product was isolated after oxidation to the corresponding cyclobutyldiol.

With a general set of reaction conditions for the [2 + 2] cycloaddition established, the scope and pivotal properties of the core structure was assessed (Scheme 1). Single point modifications of the tethered backbone were tolerated, enabling access to small 3D bicyclic scaffolds **4b**, **4c**, and **4d** containing both a boron and ester handle. Consciously aware that α,β-unsaturated esters could potentially also undergo energy transfer in the presence of high energy sensitizers [59–60], the system **4e** with two ester components was also designed. The reaction proceeded cleanly to the cycloadduct, indicating sensitization of the acrylate is also operational. Access to *vicinal* boron scaffolds **4f** and **4g** provided conclusive evidence that sensitization of alkenylboronic esters is achievable using xanthone, with in situ oxidation enabling direct access to otherwise challenging to synthesize cyclobutyldiols. The lower observed diastereoselectivity may reflect differences in stereoelectronic stabilization of the transient 1,4-biradical intermediate on changing substituent (CO_2_R→BPin) [63–65]. It is pertinent to note that increased catalyst loading and reaction times were necessary for efficient reactivity with alkenylboronic esters, suggesting acrylates are more efficiently sensitized (lower T_1_ excited state) than their alkenylboronic ester counterparts. This is unsurprising given their enhanced conjugation (4π electrons vs 2π electrons and p-orbital). To probe the efficiency of trisubstituted alkenes, *vicinal* boron precursor **3h** was designed (Scheme 1, bottom). Despite the additional substituent, the reaction was tolerated, albeit with decreased diastereoselectivity in comparison to previous alkenylboronic ester examples **4b**–**d**.

**Scheme 2:**
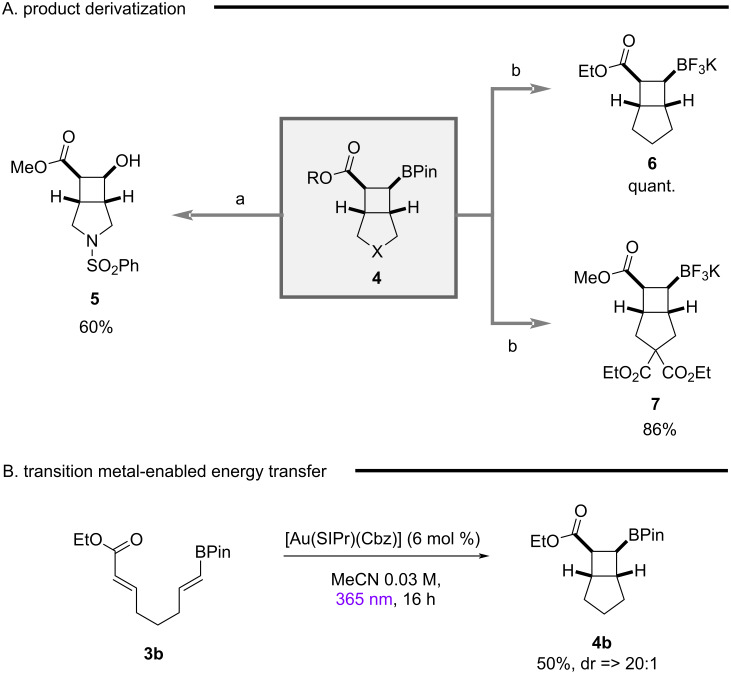
A) Product derivatization and B) transition-metal EnT catalysis. Reaction conditions A): **4d** (1 equiv), H_2_O_2_ (30 wt % in H_2_O), aq NaH_2_PO_4_, THF, 0 °C; B) **4** (1 equiv), KF (4 equiv), ʟ-tartaric acid (2.1 equiv), MeOH, MeCN, H_2_O, rt.

To demonstrate the synthetic utility of the generated small 3D fragments and complement existing [2 + 2] strategies via direct excitation [66–67], product derivatization was explored (Scheme 2A). While initial efforts for oxidation proved challenging, due to a competing retro-aldol ring-opening reaction, employing a buffered system enabled access to β-alcohol **5**. Given the previous power of trifluoroborates in cross-coupling strategies for cyclobutyl scaffolds [53–55], products **6** and **7** could be synthesized via mild conditions [68].

Inspired by recent advances by Nolan and co-workers demonstrating the synthetic power of gold catalysts in EnT catalysis [31,69–72], we probed the reactivity of [Au(SIPr)(Cbz)] in our model system given the excited state energy (67 kcal/mol) is between both active catalysts thioxanthone (65 kcal/mol) and xanthone (74 kcal/mol). While a higher catalyst loading than conventional systems was required for efficient reactivity (Scheme 2B), the [2 + 2] cycloaddition could be readily achieved. It is pertinent to note the enhanced levels of diastereoselectivity obtained for this reaction further underscore the potential of gold catalysts for future EnT reactions. Control reactions indicate selective activation of the α,β-unsaturated ester (see Supporting Information File 1 for full details).

## Conclusion

In summary, photoactivation of simple alkenylboronic esters was established using alkene scrambling as a rapid reaction probe to determine the catalyst and boron moiety for sensitization. The use of cyclic voltammetry, UV–vis analysis, and control reaction probes support sensitization as an operational mechanism. While intermolecular alkene quenching to enable [2 + 2] cycloaddition was inefficient, presumably due to poor substrate lifetime, intramolecular [2 + 2] reactivity with both acrylates and alkenylboronic esters was effective. The small 3D, boron-containing fragments could be derivatized to demonstrate the synthetic utility of the process. Although reactivity is currently limited to intramolecular quenching, it is envisaged the developed insights will serve as a blueprint for future endeavors to achieve sensitization using high energy photocatalysts, especially when considering recent advances enabling subsequent intramolecular H-atom abstraction [51] and efficient rearrangements [73–74].

## Supporting Information

File 1 Experimental section, characterization, and copies of spectra.

## Data Availability

All data that supports the findings of this study is available in the published article and/or the supporting information of this article.
