# Supplementary material for: Light-enabled intramolecular [2 + 2] cycloaddition via photoactivation of simple alkenylboronic esters
Source: Beilstein J Org Chem. 2025 Apr 30;21:854–63. doi: 10.3762/bjoc.21.69 (PMC12051466; doi:10.3762/bjoc.21.69)
Supplement: File 1 — Experimental section, characterization, and copies of spectra. [file Beilstein_J_Org_Chem-21-854-s001.pdf]

BEILSTEIN JOURNAL OF ORGANIC CHEMISTRY

Supporting Information

for

Light-enabled intramolecular [2 + 2] cycloaddition via photoactivation of simple alkenylboronic esters

Lewis McGhie, Hannah M. Kortman, Jenna Rumpf, Peter H. Seeberger and John J. Molloy

Beilstein J. Org. Chem. **2025,** *21,* 854–863. doi:10.3762/bjoc.21.69

Experimental section, characterization, and copies of spectra

License and Terms: This is a supporting information file under the terms of the Creative Commons Attribution License (https://creativecommons.org/licenses/by/4.0). Please note that the reuse, redistribution and reproduction in particular requires that the author(s) and source are credited and that individual graphics may be subject to special legal provisions.
The license is subject to the *Beilstein Journal of Organic Chemistry* terms and conditions: (https://www.beilstein-journals.org/bjoc/terms)

Table of contents

General information S2
Synthesis of starting material S3
Vinylboronic esters S3
[2 + 2] Precursors S6
Experimental setup for photoreactions S15
Reaction probe: alkene Isomerization via energy transfer S16
Reaction optimization: [2 + 2] cycloaddition S18
Substrate scope S20
Mechanistic studies S25
Control reactions S25
Cyclic voltammetry studies S28
UV–vis analysis S30
Derivatization S31
References S33
NMR Spectra S34

General information

All chemicals were purchased as reagent grade and used without further purification unless stated otherwise. Dry solvents were obtained by passing solvents through activated alumina columns and storing them over activated 4 Å molecular sieves for 24 hours prior to use. Degassed solvent refers to bubbling argon through the solvent for a minimum of 15 min. Solvents for purification (extraction and chromatography) were purchased as technical grade and distilled on the rotary evaporator prior to use. For column chromatography SiO_2 (40–63 µm for flash chromatography, Macherey Nagel or VWR) was used as a stationary phase. Analytical thin-layer chromatography (TLC) was performed on pre-coated TLC sheets ALUGRAM® XtraSIL G/UV254 (Macherey Nagel). UV light (254 nm), potassium permanganate (KMnO4), vanillin and *p*-anisaldehyde stain solutions were used for visualization. Concentration under reduced pressure was performed at ≈ 10 mbar and 40 °C, drying at ≈ 10^{-2} mbar and room temperature. NMR Spectra were measured on either a Varian 400 MHz, Bruker Ascend 400 MHz, Varian 600 MHz or Bruker Ascend 700 MHz at room temperature. The chemical shifts are referenced to the residual solvent peak as internal standard and are reported in ppm. The resonance multiplicity is abbreviated as: s (singlet), d (doublet), t (triplet), q (quartet), quint (quintet), m (multiplet) and br (broad). Assignments of unknown compounds are based on APT, DEPT, COSY(HH), HMBC, HSQC and NOESY spectra. Carbon atoms bearing boron were not observed by ^{13}C NMR and are not reported. High-resolution mass spectra were measured by the MS service of Freie Universität Berlin. UV–vis spectra were recorded using a Shimadzu UV-1900 I spectrophotometer. Cyclic voltammetry was conducted on an Interface 1000 Gamry potentiostat using a 3-electrode cell configuration. IR spectra were recorded on a Perkin-Elmer Spectrum 100 FT-IR spectrometer, selected adsorption bands are reported in wavenumbers (cm^{-1}). Photoreactions were performed using LG Innotek UV SMD-LED 3535, LEUVA35T01RL01 1.8 W lamps (365 nm), WINGER WEPUV3-S2 UV Power LED Star (Blacklight) 1.2 W (400 nm), and WINGER WEPRB3-S1 Power LED Star royal blue 3 W (450 nm). Photoreaction setup, including light source emission is comprehensively described (vide infra).

Synthesis of starting material

Vinylboronic esters

(*E*)-Oct-1-en-1-ylboronic acid (S1)

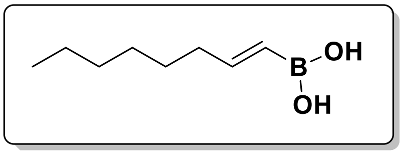

To a solution of 1-octyne (0.88 mL, 6 mmol, 1 equiv) in anhydrous DCM (3 mL, 2 M) under argon was added dibromoborane dimethyl sulfide complex solution (1 M in methylene chloride, 6 mL, 6 mmol, 1 equiv) dropwise at 0 °C. The reaction mixture was gradually warmed to room temperature and stirred for 5 hours. Upon completion, the reaction mixture was cooled to 0 °C and quenched with sat. aq. NH_4Cl. The reaction mixture was then warmed to room temperature and stirred for a further 1 hour. The reaction mixture was extracted with Et_2O (3 × 10 mL), and the combined organics washed with sat. aq. $NaHCO_3$, then washed with brine, dried over Na_2SO_4, filtered and concentrated under reduced pressure to afford **S1** as a beige solid (811 mg, 5.2 mmol, 87% yield).

^{1}H NMR (400 MHz, CD_3OD): δ = 6.53 (dt, *J* = 17.5, 6.6 Hz, 1H), 5.56 (dt, *J* = 17.7, 1.5 Hz, 1H), 2.20 – 2.11 (m, 2H), 1.47 – 1.37 (m, 2H), 1.35 – 1.27 (m, 6H), 0.91 (t, *J* = 6.5 Hz, 3H) ppm; analytical data in agreement with literature.[1]

(*E*)-Oct-1-en-1-ylboronic acid, pinacol ester (*E*-1a)

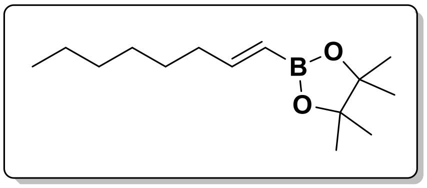

To an oven-dried Schlenk flask was added 1-octyne (1.5 mL, 10 mmol, 1 equiv) and bis(cyclopentadienyl)zirconium chloride hydride (258 mg, 1 mmol, 0.1 equiv), and the flask placed under a nitrogen atmosphere. To the flask was added 1,2-DCE (3.5 mL, 2.9 M) and the reaction mixture was cooled to 0 °C before the dropwise addition of 4,4,5,5-tetramethyl-1,3,2-dioxaborolane (1.6 mL, 11 mmol, 1.1 equiv) under a positive nitrogen flow. The reaction mixture was then sealed and stirred at 60 °C for 20 h. The reaction mixture was cooled to room temperature before the dropwise addition of MeOH (60 μL, 1.5 mmol, 0.15 equiv) and concentration under reduced pressure to afford a crude residue. The crude material was purified by flash column chromatography (SiO_2, 0→3% EtOAc/*n*-hexane) to afford ***E*-1a** as a colorless oil (1.86 g, 7.8 mmol, 78% yield).

^{1}H NMR (400 MHz, $CDCl_3$): δ = 6.63 (dt, *J* = 18.0, 6.4 Hz, 1H), 5.42 (dt, *J* = 18.0, 1.6 Hz, 1H), 2.18 – 2.10 (m, 2H), 1.45 – 1.36 (m, 2H), 1.33 – 1.21 (m, 18H), 0.91 – 0.83 (m, 3H) ppm; analytical data in agreement with literature.[2]

Oct-1-yn-1-ylboronic acid pinacol ester (S2)

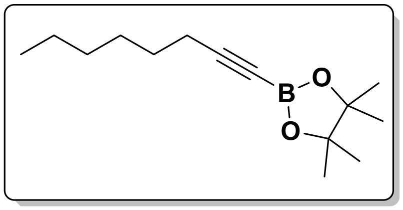

S2 was prepared according to a procedure of Batey et al.[3] To an oven-dried round-bottomed flask was added 1-octyne (0.89 mL, 6 mmol, 1.2 equiv) and anhydrous THF (10 mL, 0.6 M) under an argon atmosphere before being cooled to −78 °C. To the reaction mixture was added *n*-BuLi (2.5 mL, 2.4 M in hexanes, 6 mmol, 1.2 equiv) dropwise and the reaction mixture stirred for 1 hour. The resulting mixture was added dropwise to a solution of 2-isopropoxy-4,4,5,5-tetramethyl-1,3,2-dioxaborolane (1 mL, 5 mmol, 1 equiv) in THF (10 mL, 0.5 M) under an argon atmosphere at −78 °C. The reaction mixture was stirred for 2 hours before being quenched with HCl (2.5 mL, 3 M in CPME, 7.5 mmol, 1.5 equiv). The

reaction mixture was gradually warmed to room temperature with an additional 1 hour of stirring. The reaction mixture was filtered and concentrated under reduced pressure to afford a crude residue. The crude residue was dissolved in *n*-pentane (5 mL), filtered and concentrated under reduced pressure to afford **S2** as a colorless oil (703 mg, 3 mmol, 50% yield).

^{1}H NMR (400 MHz, $CDCl_3$): δ = 2.25 (t, *J* = 7.2 Hz, 2H), 1.58 – 1.48 (m, 3H), 1.44 – 1.27 (m, 2H), 1.27 (s, 15H), 0.87 (t, *J* = 6.9 Hz, 3H) ppm; analytical data in agreement with literature.[3]

(*Z*)-Oct-1-en-1-ylboronic acid pinacol ester (*Z*-1a)

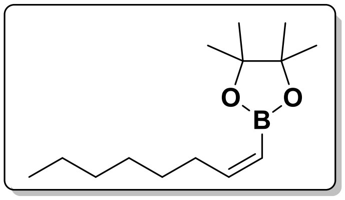

***Z*-1a** was prepared according to a procedure of Morken et al.[4] To an oven-dried round-bottomed flask was added bis(cyclopentadienyl)zirconium chloride hydride (284 mg, 1.1 mmol, 1.1 equiv) and the flask placed under an argon atmosphere. To the flask was added anhydrous THF (3 mL, 0.3 M) and **S2** (236 mg, 1 mmol, 1 equiv), and the reaction mixture stirred for 1 hour. The reaction mixture was then cooled to 0 °C before the dropwise addition of HCl (0.37 mL, 3 M in CPME, 1.1 mmol, 1.1 equiv). The reaction mixture was warmed to room temperature and stirred for 30 minutes. The solvent was then removed under reduced pressure to afford a crude residue. The crude material was purified by flash column chromatography (SiO_2, 0→5% EtOAc/*n*-hexane) to afford ***Z*-1a** as a colorless oil (152 mg, 0.64 mmol, 64% yield).

^{1}H NMR (400 MHz, $CDCl_3$): δ = 6.43 (dt, *J* = 14.2, 7.5 Hz, 1H), 5.32 (dt, *J* = 13.5, 1.3 Hz, 1H), 2.39 (qd, *J* = 7.3, 1.4 Hz, 2H), 1.41 – 1.22 (m, 20H), 0.91 – 0.84 (m, 3H) ppm; analytical data in agreement with literature.[3]

(*E*)-Oct-1-en-1-ylboronic acid catechol ester (*E*-1b)

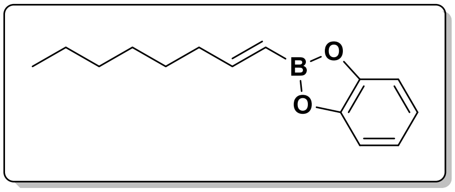

To an oven-dried round-bottomed flask was added 1-octyne (0.44 mL, 3 mmol, 1 equiv) and the flask placed under an argon atmosphere. To the reaction flask was added catecholborane (1 M in THF, 3.6 mL, 3.6 mmol, 1.2 equiv) dropwise before the reaction mixture was heated to 70 °C and stirred for 2 hours. The reaction mixture was cooled to room temperature and concentrated under reduced pressure. The crude material was purified by flash column chromatography (SiO_2, 0→5% EtOAc/*n*-hexane) to afford ***E*-1b** as a pale yellow oil (92 mg, 0.4 mmol, 13% yield).

^{1}H NMR (400 MHz, $CDCl_3$): δ = 7.21 (dd, *J* = 5.8, 3.3 Hz, 2H), 7.10 – 6.99 (m, 3H), 5.79 (dt, *J* = 18.1, 1.6 Hz, 1H), 2.33 – 2.24 (m, 2H), 1.53 – 1.45 (m, 2H), 1.39 – 1.24 (m, 6H), 0.94 – 0.86 (m, 3H) ppm; analytical data in agreement with literature.[5]

(*E*)-Oct-1-en-1-ylboronic acid neopentyl glycol ester (*E*-1c)

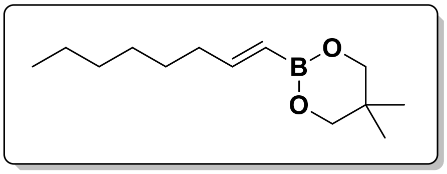

To an oven-dried round-bottomed flask was added **S1** (156 mg, 1 mmol, 1 equiv), neopentylglycol (115 mg, 1.1 mmol, 1.1 equiv), and $MgSO_4$ (160 mg, 1.3 mmol, 1.3 equiv) before being placed under an argon atmosphere. Anhydrous THF (7 mL, 0.14 M) was added to the reaction mixture and the reaction stirred for 15 hours. The reaction mixture was then filtered and concentrated under reduced pressure. The crude material was purified by flash column chromatography (SiO_2, 0→5% EtOAc/*n*-hexane) to afford ***E*-1c** as a pale yellow oil (92.2 mg, 0.41 mmol, 41% yield).

^{1}H NMR (400 MHz, $CDCl_3$): δ = 6.54 (dt, *J* = 17.8, 6.5 Hz, 1H), 5.34 (dt, *J* = 17.8, 1.6 Hz, 1H), 3.63 (s, 4H), 2.18 – 2.07 (m, 2H), 1.45 – 1.35 (m, 2H), 1.33 – 1.23 (m, 6H), 0.97 (s, 6H), 0.91 – 0.84 (m, 3H) ppm; analytical data in agreement with literature.[6]

(*E*)-2-(Oct-1-en-1-yl)-4,4,5,5-tetraphenyl-1,3,2-dioxaborolane (*E*-1d)

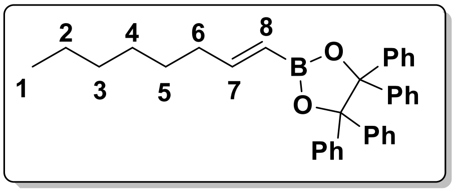

To an oven-dried round-bottomed flask was added **S1** (125 mg, 0.8 mmol, 1 equiv) and benzopinacol (293 mg, 0.8 mmol) before being placed under an argon atmosphere. Anhydrous toluene (1.7 mL, 0.5 M) was added to the reaction mixture and the reaction mixture was heated to 90 °C and stirred for 16 hours. The reaction mixture was cooled to room temperature and the solvent removed under reduced pressure to afford a crude residue. The crude material was purified by flash column chromatography (SiO_2, 0→5% EtOAc/*n*-hexane) to afford ***E*-1d** as a colourless oil (263 mg, 0.54 mmol, 68% yield).

^{1}H NMR (400 MHz, $CDCl_3$): δ = 7.21 – 7.12 (m, 8H, Ar-H), 7.10 – 7.00 (m, 13H, H7 and Ar-H), 5.81 (dt, *J* = 18.0, 1.6 Hz, 1H, H8), 2.33 – 2.25 (m, 2H, H6), 1.54 – 1.47 (m, 2H, H5), 1.41 – 1.27 (m, 6H, H4, H3, and H2), 0.94 – 0.87 (m, 3H, H1) ppm; **^{13}C NMR** (101 MHz, $CDCl_3$): δ = 157.3, 142.8, 128.7, 127.3, 127.0, 95.8, 36.2, 31.9, 29.2, 28.3, 22.8, 14.3 ppm; **IR** (ATR): 2926, 1635, 1493, 1446, 1401, 1364, 1328, 1283, 1219, 1174, 1035, 887, 749, 695, 659, 643 cm^{-1}; **HRMS** (EI) calc. for $C_{34}H_{35}BO_2$ $[M]^+$ 486.2725, found 486.2730.

(*E*)-Oct-1-en-1-ylboronic acid, MIDA ester (*E*-1e)

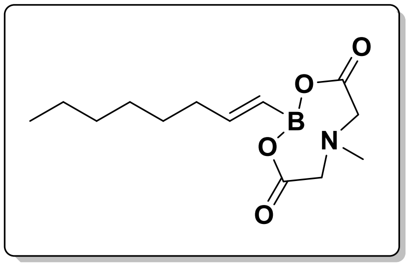

To an oven-dried round-bottomed flask was added **S1** (156 mg, 1 mmol, 1 equiv) and methyliminodiacetic acid (147 mg, 1 mmol, 1 equiv) before being placed under an argon atmosphere. Anhydrous DMF (7 mL, 0.14 M) was added and the reaction mixture stirred at 70 °C for 15 hours. The reaction mixture was cooled to room temperature before the addition of cold Et_2O to form a white precipitate. The precipitate was isolated via filtration to afford ***E*-1e** as a white solid (26 mg, 0.1 mmol, 10% yield).

^{1}H NMR (400 MHz, acetone-*d*$_6$): δ = 6.09 (dt, *J* = 17.6, 6.4 Hz, 1H), 5.47 (dt, *J* = 17.6, 1.6 Hz, 1H), 4.18 (d, *J* = 16.8 Hz, 2H), 3.98 (d, *J* = 16.8 Hz, 2H), 2.98 (s, 3H), 2.16 – 2.08 (m, 2H), 1.44 – 1.36 (m, 2H), 1.33 – 1.26 (m, 6H), 0.91 – 0.84 (m, 3H) ppm; analytical data in agreement with literature.[7]

(*E*)-2-(Oct-1-en-1-yl)-2,3-dihydro-1*H*-naphtho[1,8-*de*][1,3,2]diazaborinine (*E*-1f)

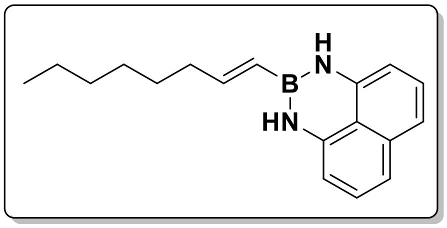

To an oven-dried round-bottomed flask was added **S1** (445 mg, 2.9 mmol, 1 equiv), 1,8-diaminonaphthalene (451 mg, 2.9 mmol, 1 equiv), and 4 Å molecular sieves (2.6 g) before being placed under an argon atmosphere. Anhydrous toluene (25 mL, 0.12 M) was added to the reaction mixture and the reaction stirred at 110 °C for 15 hours. The reaction mixture was cooled to room temperature and filtered through a pad of celite. The filtrate was diluted with H_2O (30 mL) and organics extracted with EtOAc (3 x 25 mL). The combined organics were washed with brine, dried over Na_2SO_4, filtered and concentrated under reduced pressure. The crude material

was purified by flash column chromatography (SiO_2, 0→5% EtOAc/*n*-hexane) to afford ***E*-1f** as a pale yellow oil (600 mg, 2.2 mmol, 76% yield).

^{1}H NMR (400 MHz, $CDCl_3$): δ = 7.09 (dd, *J* = 8.3, 7.2 Hz, 2H), 7.00 (d, *J* = 8.2 Hz, 2H), 6.41 – 6.29 (m, 3H), 5.70 (s, 2H), 5.56 (dt, *J* = 18.0, 1.5 Hz, 1H), 2.24 – 2.16 (m, 2H), 1.49 – 1.39 (m, 2H), 1.35 – 1.24 (m, 6H), 0.93 – 0.87 (m, 3H) ppm; analytical data in agreement with literature.[6]

[2 + 2] Precursors

General procedure A: hydroboration of terminal alkynes

The reaction was performed according to a modified procedure of Yoon et al.[8] To an oven-dried Schlenk flask was added alkyne (1 equiv) and bis(cyclopentadienyl)zirconium chloride hydride (0.1 equiv), and the flask placed under a nitrogen atmosphere. To the flask was added 1,2-DCE (3 M) and the reaction mixture cooled to 0 °C before the dropwise addition of 4,4,5,5-tetramethyl-1,3,2-dioxaborolane (1.1 equiv) under a positive nitrogen flow. The reaction mixture was then sealed and stirred at 60 °C for 20 h. The reaction mixture was cooled to room temperature before the dropwise addition of MeOH (0.15 equiv) and concentration under reduced pressure to afford a crude residue. The crude material was purified by flash column chromatography (SiO_2, specified combination of solvents).

Diethyl 2-allylmalonate (S3)

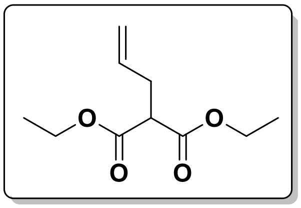

S3 was prepared according to a procedure of Palomo et al.[9] To an oven-dried round-bottomed flask was added potassium carbonate (4.1 g, 30 mmol, 3 equiv). The flask was sealed and purged with argon before the sequential addition of anhydrous acetone (50 mL, 0.6 M), diethyl malonate (2.3 mL, 15 mmol, 1.5 equiv), and allyl bromide (0.86 mL, 10 mmol, 1 equiv). The reaction mixture was stirred at room temperature for 24 h. After completion, the reaction mixture was quenched with sat. aq. NH_4Cl and extracted with ethyl acetate (3 × 50 mL). The combined organic phases were washed with brine, dried over Na_2SO_4, filtered and concentrated under reduced pressure. The resulting crude material was purified via flash column chromatography (SiO_2, 0→10% EtOAc/*n*-hexane) to yield **S3** as a colorless oil (1.02 g, 5.1 mmol, 51% yield).

^{1}H NMR (400 MHz, $CDCl_3$): δ = 5.78 (ddt, *J* = 17.0, 10.2, 6.8 Hz, 1H), 5.16 – 5.03 (m, 2H), 4.20 (qd, *J* = 7.1, 1.6 Hz, 4H), 3.42 (t, *J* = 7.6 Hz, 1H), 2.64 (ddt, *J* = 7.9, 6.8, 1.3 Hz, 2H), 1.26 (t, *J* = 7.1 Hz, 6H) ppm; analytical data in agreement with literature.[9]

Diethyl 2-allyl-2-(prop-2-yn-1-yl)malonate (S4)

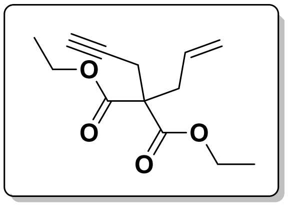

S4 was prepared according to a procedure of Yoon et al.[10] To an oven-dried round-bottomed flask was added NaH 60% in mineral oil (152 mg, 6.4 mmol, 1.25 equiv) before being sealed and placed under an argon atmosphere. To the flask was added anhydrous THF (20 mL, 0.3 M) and the reaction mixture cooled to 0 °C before the dropwise addition of **S3** (1.02 g, 5.1 mmol, 1 equiv). The reaction mixture was stirred at room temperature for 10 minutes before the dropwise addition of propargyl bromide (0.6 mL, 6.1 mmol, 1.2 equiv) at 0 °C. The reaction mixture was gradually warmed to room temperature and stirred for 18 h. After completion, the reaction mixture was quenched with sat. aq. NH_4Cl and extracted with ethyl acetate (3 × 20 mL). The combined organic phases were washed with brine, dried over Na_2SO_4, filtered and concentrated under reduced pressure. The resulting

crude material was purified via flash column chromatography (SiO_2, 0→5% EtOAc/*n*-hexane) to yield **S4** as a colorless oil (1.05 g, 4.5 mmol, 88% yield).

^{1}**H NMR** (400 MHz, $CDCl_3$): δ = 5.63 (ddt, *J* = 17.4, 10.0, 7.5 Hz, 1H), 5.24 – 5.07 (m, 2H), 4.21 (q, *J* = 7.1 Hz, 4H), 2.84 – 2.77 (m, 4H), 2.01 (t, *J* = 2.7 Hz, 1H), 1.25 (t, *J* = 7.1 Hz, 6H) ppm; analytical data in agreement with literature.[10]

(*E*)-(4,4-Bis(ethoxycarbonyl)hepta-1,6-dien-1-yl)boronic acid pinacol ester (3a)

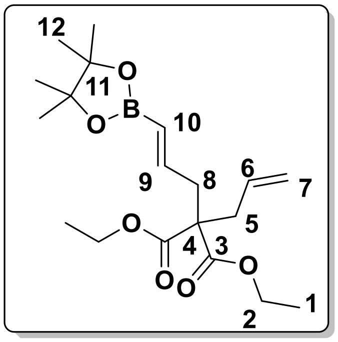

Prepared according to General Procedure A, **S4** (1.05 g, 4.5 mmol, 1 equiv) was converted to **3a**, yielding a colourless oil (789 mg, 2.2 mmol, 49%) after purification by flash column chromatography (SiO2, 0→6% EtOAc/n-hexane).

R*f* (10% EtOAc/*n*-hexane) = 0.29; ^{1}**H NMR** (400 MHz, $CDCl_3$): δ = 6.41 (dt, *J* = 17.7, 7.2 Hz, 1H, H9), 5.65 (ddt, *J* = 17.5, 10.1, 7.4 Hz, 1H, H6), 5.52 (dt, *J* = 17.6, 1.4 Hz, 1H, H10), 5.15 – 5.06 (m, 2H, H7), 4.18 (qd, *J* = 7.2, 1.6 Hz, 4H, H2), 2.74 (dd, *J* = 7.3, 1.4 Hz, 2H, H8), 2.67 – 2.61 (m, 2H, H5), 1.27 – 1.23 (m, 18H, H1, H12) ppm; ^{13}**C NMR** (100 MHz, $CDCl_3$): δ = 170.8 (C3), 147.2 (C9), 132.4 (C6), 119.4 (C7), 83.3 (C11), 61.5 (C2), 57.3 (C4), 39.0 (C8), 37.0 (C5), 24.9 (C12), 14.3 (C1) ppm; ^{11}**B NMR** (128 MHz, $CDCl_3$) δ = 29.68 ppm; **IR** (ATR): 2979, 1730, 1639, 1444, 1391, 1380, 1361, 1321, 1285, 1258, 1203, 1179, 1167, 1143, 1113, 1097, 1031, 998, 970, 920, 850, 757, 646 cm^{-1}; **HRMS** (ESI) calc. for $C_{19}H_{31}BO_6Na^+$ $[M+Na]^+$ 389.2106, found 389.2111.

Ethyl (*E*)-oct-2-en-7-ynoate (S5)

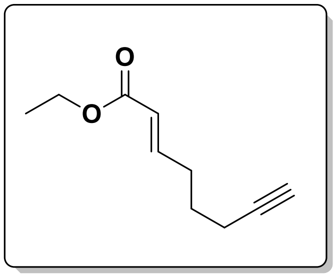

S5 was prepared according to a procedure of Breinbauer et al.[11] To an oven-dried round-bottomed flask was added oxalyl chloride (1.2 mL, 14 mmol, 1.4 equiv) and anhydrous DCM (50 mL, 0.28 M) under an argon atmosphere. The reaction mixture was cooled to −78 °C before the dropwise addition of anhydrous DMSO (1.8 mL, 25 mmol, 2.5 equiv). The reaction mixture was stirred at −78 °C for 15 minutes before the dropwise addition of 5-hexyn-1-ol (1.1 mL, 10 mmol, 1 equiv) in anhydrous DCM (12.5 mL, 0.8 M). The reaction mixture was stirred at −78 °C for a further 15 minutes before the dropwise addition of triethylamine (7.1 mL, 51 mmol, 5.1 equiv). The reaction mixture was stirred at −78 °C for a further 30 minutes, before being gradually warmed to room temperature and stirred for a further 90 minutes. To the reaction mixture was added (carbethoxymethylene)triphenylphosphorane (4.24 g, 12.2 mmol, 1.2 equiv) in one portion and the reaction stirred for 18 h. The solvent was removed under reduced pressure and the crude material purified via flash column chromatography (SiO_2, 0→10% EtOAc/*n*-hexane) to yield **S5** as a colorless oil (1.36 g, 8.2 mmol, 82%).

^{1}**H NMR** (400 MHz, $CDCl_3$): δ = 6.94 (dt, *J* = 15.7, 7.0 Hz, 1H), 5.85 (dt, *J* = 15.6, 1.6 Hz, 1H), 4.19 (q, *J* = 7.1 Hz, 2H), 2.33 (q, *J* = 7.1 Hz, 2H), 2.23 (td, *J* = 7.0, 2.6 Hz, 2H), 1.97 (t, *J* = 2.7 Hz, 1H), 1.70 (p, *J* = 7.2 Hz, 2H), 1.29 (t, *J* = 7.1 Hz, 3H) ppm; analytical data in agreement with literature.[11]

((1*E*,6*E*)-8-Ethoxy-8-oxoocta-1,6-dien-1-yl)boronic acid pinacol ester (3b)

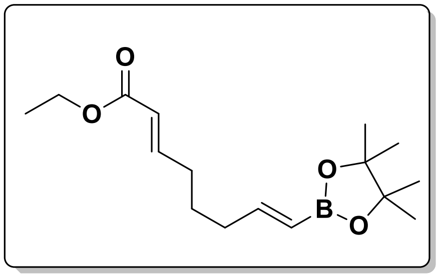

Prepared according to General Procedure A, **S5** (665 mg, 4 mmol, 1 equiv) was converted to **3b**, yielding a colourless oil (384 mg, 1.3 mmol, 33%) after purification by flash column chromatography (SiO2, 0→10% EtOAc/n-hexane).

^{1}H NMR (400 MHz, $CDCl_3$): δ = 6.94 (dt, *J* = 15.6, 6.9 Hz, 1H), 6.59 (dt, *J* = 17.9, 6.4 Hz, 1H), 5.81 (dt, *J* = 15.7, 1.6 Hz, 1H), 5.45 (dt, *J* = 18.0, 1.5 Hz, 1H), 4.18 (q, *J* = 7.1 Hz, 2H), 2.25 – 2.14 (m, 4H), 1.59 (p, *J* = 7.5 Hz, 2H), 1.32 – 1.24 (m, 15H) ppm; analytical data in agreement with literature.[12]

Diethyl 2-(2,2-diethoxyethyl)malonate (S6)

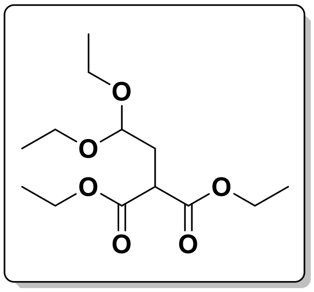

S6 was prepared according to a procedure of Procter et al.[13] To a suspension of NaH 60% in mineral oil (0.264 g, 11 mmol, 1.1 equiv) in anhydrous DMF (20 mL, 0.55 M) was added diethyl malonate (1.5 mL, 10 mmol, 1 equiv) at 0 °C under an argon atmosphere. The suspension was stirred for 30 minutes before the addition of 2-bromo-1,1-diethoxyethane (2.0 mL, 13 mmol, 1.3 equiv). The reaction mixture was gradually warmed to room temperature and then heated to 120 °C and stirred for 18 h. The reaction mixture was cooled to room temperature and quenched with sat. aq. NH_4Cl. The mixture was diluted with ethyl acetate (10 mL) and the organic layer was separated. The organic layer was washed with brine (4 × 20 mL), dried over Na_2SO_4, filtered and concentrated under reduced pressure. The crude material was used in the next step without further purification.

Diethyl 2-(2-oxoethyl)malonate (S7)

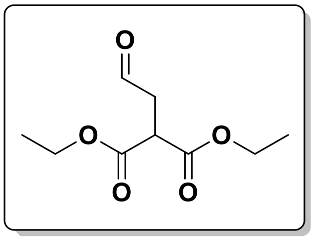

S7 was prepared according to a procedure of Procter et al.[13] **S6** was dissolved in $CHCl_3$ (8 mL) and H_2O (3 mL) and cooled to 0 °C before the dropwise addition of TFA (8 mL). The reaction mixture was stirred at 0 °C for 2 hours before being neutralized with aq. sat. K_2CO_3. The organic layer was extracted with DCM (3 × 20 mL), and the combined organic layers were washed with brine (20 mL), dried over Na_2SO_4, filtered and concentrated under reduced pressure. The crude material was used in the next step without further purification.

1,1-Diethyl 4-methyl (*E*)-but-3-ene-1,1,4-tricarboxylate (S8)

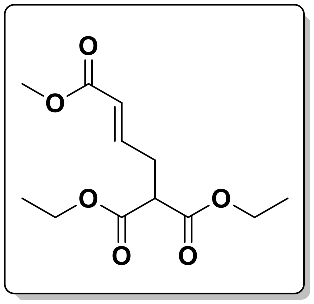

S8 was prepared according to a procedure of Procter et al.[13] **S7** was dissolved in DCM (10 mL, 1 M) under an argon atmosphere before the addition of methyl (triphenylphosphoranylidene)acetate (3.3 g, 10 mmol, 1 equiv). The reaction mixture was then stirred for 18 hours at room temperature. The solvent was then removed under reduced pressure and the crude residue purified via flash column chromatography (SiO_2, 0→10% EtOAc/*n*-hexane) to yield **S8** as a colorless oil (1.58 g, 6.1 mmol, 61% yield over three steps).

^{1}H NMR (400 MHz, $CDCl_3$): δ = 6.89 (dt, *J* = 15.6, 7.1 Hz, 1H), 5.90 (dt, *J* = 15.6, 1.5 Hz, 1H), 4.24 – 4.18 (m, 4H), 3.72 (s, 3H), 3.48 (t, *J* = 7.4 Hz, 1H), 2.79 (td, *J* = 7.3, 1.6 Hz, 2H), 1.27 (t, *J* = 7.1 Hz, 6H) ppm; analytical data in agreement with literature.[14]

4,4-Diethyl 1-methyl (*E*)-hept-1-en-6-yne-1,4,4-tricarboxylate (S9)

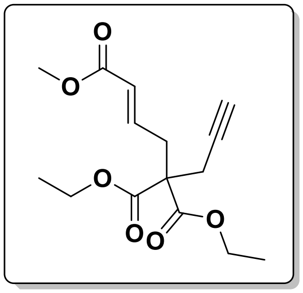

S9 was prepared according to a procedure of Takemoto et al.[15] To an oven-dried round-bottomed flask was added cesium carbonate (4 g, 12.3 mmol, 2 equiv). The flask was sealed and purged with argon before the sequential addition of anhydrous acetone (30 mL, 0.4 M), **S8** (1.58 g, 6.1 mmol, 1 equiv), and propargyl bromide (0.94 mL, 9.2 mmol, 1.5 equiv). The reaction mixture was stirred at room temperature for 18 h. The mixture was filtered, washed with acetone (3 × 20 mL), and the solvent removed under recued pressure. The crude residue was purified via flash column chromatography (SiO_2, 0→10% EtOAc/*n*-hexane) to yield **S9** as a colorless oil (1.18 g, 4.0 mmol, 65% yield).

^{1}H NMR (400 MHz, $CDCl_3$): δ = 6.78 (dt, *J* = 15.6, 7.8 Hz, 1H), 5.95 (dt, *J* = 15.4, 1.4 Hz, 1H), 4.22 (td, *J* = 7.2, 6.6 Hz, 4H), 3.72 (s, 3H), 2.95 (dd, *J* = 7.8, 1.4 Hz, 2H), 2.80 (d, *J* = 2.7 Hz, 2H), 2.05 (t, *J* = 2.7 Hz, 1H), 1.26 (t, *J* = 7.1 Hz, 6H) ppm; analytical data in agreement with literature.[16]

((1*E*,6*E*)-4,4-Bis(ethoxycarbonyl)-8-methoxy-8-oxoocta-1,6-dien-1-yl)boronic acid, pinacol ester (3c)

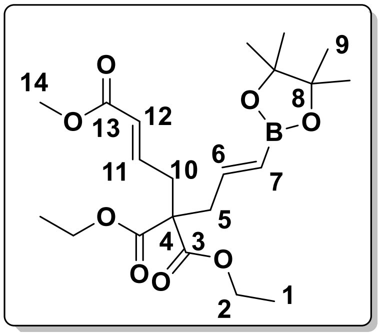

Prepared according to General Procedure A, **S9** (1.87 g, 6.3 mmol, 1 equiv) was converted to **3c**, yielding a colorless oil (824 mg, 1.9 mmol, 31%) after purification by flash column chromatography (SiO_2, 0→10% EtOAc/n-hexane).

R*f* (20% EtOAc/*n*-hexane) = 0.40; **^{1}H NMR** (400 MHz, $CDCl_3$): δ = 6.80 (ddd, *J* = 15.5, 8.1, 7.3 Hz, 1H, H11), 6.38 (dt, *J* = 17.7, 7.2 Hz, 1H, H6), 5.90 – 5.83 (m, 1H, H12), 5.58 – 5.50 (m, 1H, H7), 4.24 – 4.15 (m, 4H, H2), 3.73 – 3.70 (m, 3H, H14), 2.75 (ddd, *J* = 7.3, 4.3, 1.4 Hz, 4H, H5, H10), 1.28 – 1.23 (m, 18H, H1, H9) ppm; **^{13}C NMR** (100 MHz, $CDCl_3$): δ = 170.2 (C3), 166.4 (C13), 146.5 (C6), 143.0 (C11), 124.9 (C12), 83.4 (C8), 61.8 (C2), 57.0 (C4), 51.7 (C14), 39.4 (C5), 35.5 (C10), 24.9 (C9), 24.7 (C9), 14.2 (C1) ppm; **^{11}B NMR** (128 MHz, $CDCl_3$) δ = 29.59 ppm; **IR** (ATR): 2979, 1726, 1660, 1639, 1437, 1391, 1362, 1324, 1272, 1249, 1166, 1143, 1113, 1097, 1034, 1005, 983, 970, 926, 848, 757, 676, 650 cm^{-1}; **HRMS** (ESI) calc. for $C_{21}H_{33}BO_8Na^+$ $[M+Na]^+$ 447.2160, found 447.2124.

((1*E*,6*E*)-4,4-Bis(ethoxycarbonyl)-8-methoxy-8-oxoocta-1,6-diene-1,2-diyl)diboronic acid pinacol ester (3h)

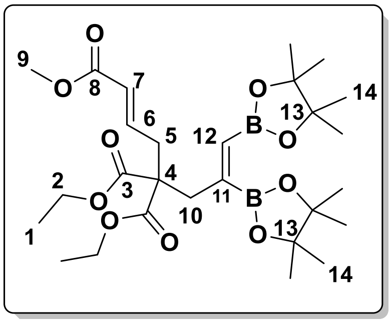

3h was prepared according to a literature procedure of Miyaura et al.[17] To an oven-dried microwave vial were added **S9** (889 mg, 3 mmol, 1 equiv), B_2Pin_2 (761 mg, 3 mmol, 1 equiv), and $Pt(PPh_3)_4$ (111 mg, 0.09 mmol, 3 mol %) before it was sealed and purged with nitrogen. The reaction mixture was heated to 100 °C and stirred for 24 h. Upon completion, reaction mixture was concentrated under reduced pressure. The crude material was purified via flash column chromatography (SiO_2, 0→7.5% EtOAc/*n*-hexane) to yield **3h** as a colorless oil (819 mg, 1.49 mmol, 50%).

R*f* (20% Acetone/*n*-hexane) = 0.35; **^{1}H NMR** (400 MHz, $CDCl_3$) δ = 6.97 (ddd, *J* = 17.8, 8.9, 6.6 Hz, 1H, H6), 5.96 (s, 1H, H12), 5.82 (dt, *J* = 15.5, 1.5 Hz, 1H, H7), 4.31 – 4.00 (m, 4H, H2), 3.70 (d, *J* = 2.5 Hz, 3H, H9), 2.89 (s, 2H, H10), 2.74 (dd, *J* = 7.6, 1.5 Hz, 2H, H5), 1.27 (s, 12H, H14), 1.26 – 1.24 (m, 12H,

H14), 1.22 (t, *J* = 7.1 Hz, 6H, H1) ppm; **^{13}C NMR** (100 MHz, $CDCl_3$) δ = 170.5 (C3), 166.6 (C8), 144.5 (C6), 124.1 (C7), 84.1 (C13), 83.6 (C13), 61.5 (C2), 58.4 (C4), 51.5 (C9), 41.3 (C10), 35.5 (C5), 25.1 (C14), 25.0 (C14), 14.2 (C1) ppm; **^{11}B NMR** (128 MHz, $CDCl_3$) δ = 30.09 ppm; **IR** (ATR): 2979, 1726, 1657, 1612, 1436, 1405, 1371, 1332, 1312, 1272, 1242, 1193, 1167, 1138, 1111, 1051, 967, 861, 850, 833, 724, 702, 668; **HRMS** (ESI) calc. for $C_{27}H_{44}B_2O_{10}Na^+$ $[M+Na]^+$ 573.3013, found 573.3040.

***N*-(Prop-2-yn-1-yl)benzenesulfonamide (S10) and *N,N*-di(prop-2-yn-1-yl)benzenesulfonamide (S11)**

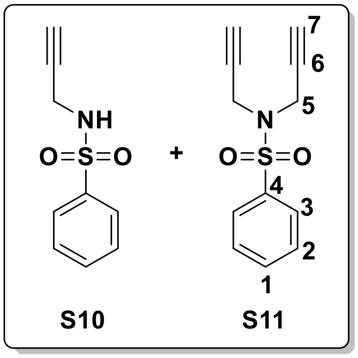

S10 and **S11** were prepared according to a modified procedure of Artok et al.[18] To an oven-dried round-bottomed flask was added anhydrous K_2CO_3 (1.4 g, 10 mmol, 1 equiv) and benzenesulfonamide (6.3 g, 40 mmol, 4 equiv). The flask was sealed and purged with argon before the addition of anhydrous MeCN (40 mL, 1 M) and propargyl bromide (1 mL, 10 mmol, 1 equiv). The reaction mixture was heated to reflux and stirred for 2 h. The reaction mixture was gradually cooled to room temperature, quenched with H_2O, and extracted with EtOAc (3 × 20 mL). The combined organics were washed with brine, dried over Na_2SO_4, filtered and concentrated under reduced pressure. The crude material was purified via flash column chromatography (SiO_2, 0→20% EtOAc/*n*-hexane) to yield **S10** as a white solid (759 mg, 3.9 mmol, 39%) and **S11** as a white solid (570 mg, 2.4 mmol, 24%).

For S10: ^{1}H NMR (400 MHz, $CDCl_3$): δ = 7.93 – 7.87 (m, 2H), 7.63 – 7.57 (m, 1H), 7.56 – 7.49 (m, 2H), 4.59 (s, 1H), 3.86 (dd, *J* = 6.1, 2.5 Hz, 2H), 2.09 (t, *J* = 2.5 Hz, 1H) ppm; analytical data in agreement with literature.[19]

For S11: R*f* (50% EtOAc/*n*-hexane) = 0.77; **^{1}H NMR** (400 MHz, $CDCl_3$): δ = 7.87 – 7.81 (m, 2H, H3), 7.63 – 7.57 (m, 1H, H1), 7.55 – 7.48 (m, 2H, H2), 4.19 (d, *J* = 2.4 Hz, 4H, H5), 2.14 (t, *J* = 2.4 Hz, 2H, H7) ppm; **^{13}C NMR** (101 MHz, $CDCl_3$): 138.3 (C4), 133.3 (C1), 129.1 (C2), 128.0 (C3), 76.2 (C6), 74.2 (C7), 36.4 (C5) ppm; **IR** (ATR): 3276, 3262, 2969, 2936, 2890, 2847, 2122, 1907, 1588, 1483, 1449, 1443, 1428, 1362, 1337, 1319, 1312, 1294, 1248, 1163, 1126, 1094, 1070, 1026, 1001, 958, 925, 887, 768, 745, 712, 699, 685, 669, 609 cm^{-1}; **HRMS** (ESI) calc. For $C_{12}H_{11}NO_2SK^+$ $[M+K]^+$ 272.0142, found 272.0264.

Methyl (*E*)-4-(*N*-(prop-2-yn-1-yl)phenylsulfonamido)but-2-enoate (S12)

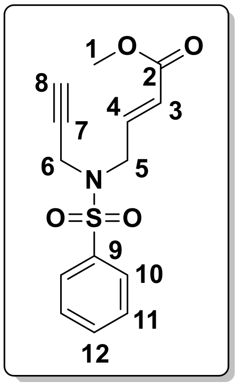

S12 was prepared according to a modified procedure of Artok et al.[18] To an oven-dried round-bottomed flask was added anhydrous K_2CO_3 (2.07 g, 15 mmol, 2 equiv) and **S10** (1.46 g, 7.5 mmol, 1 equiv). The flask was sealed and purged with argon before the addition of anhydrous MeCN (40 mL, 0.19 M) and methyl (*E*)-4-bromobut-2-enoate (1.8 mL, 15 mmol, 2 equiv). The reaction mixture was heated to reflux and stirred for 3 h. The reaction mixture was gradually cooled to room temperature, quenched with H_2O, and extracted with EtOAc (3 × 30 mL). The combined organics were washed with brine, dried over Na_2SO_4, filtered, and concentrated under reduced pressure. The crude material was purified via flash column chromatography (SiO_2, 0→20% EtOAc/*n*-hexane) to yield **S12** as a white solid (1.93 g, 6.6 mmol, 88%).

R*f* (20% EtOAc/*n*-hexane) = 0.21; **^{1}H NMR** (400 MHz, $CDCl_3$): δ = 7.89 – 7.83 (m, 2H, H10), 7.64 – 7.57 (m, 1H, H12), 7.56 – 7.49 (m, 2H, H11), 6.81 (dt, *J* = 15.7, 5.9 Hz, 1H, H4), 6.05 (dt, *J* = 15.6, 1.6 Hz, 1H, H3), 4.11 (d, *J* = 2.5 Hz, 2H, H6), 4.01 (dd, *J* = 5.9, 1.6 Hz, 2H, H5), 3.75 (s, 3H, H1), 2.03 (t, *J* = 2.4 Hz,

1H, H8) ppm; **^{13}C NMR** (101 MHz, $CDCl_3$): δ = 166.1 (C2), 141.6 (C4), 138.7 (C9), 133.2 (C12), 129.2 (C11), 127.8 (C10), 124.5 (C3), 76.0 (C7), 74.5 (C8), 51.9 (C1), 47.2 (C5), 36.8 (C6) ppm; **IR** (ATR): 3250, 2120, 1713, 1662, 1585, 1447, 1433, 1367, 1352, 1335, 1304, 1276, 1250, 1202, 1161, 1128, 1088, 1072, 1014, 998, 981, 949, 896, 851, 767, 739, 725, 715, 686, 614 cm^{-1}; **HRMS** (ESI) calc. For $C_{14}H_{15}NO_4SNa^+$ $[M+Na]^+$ 316.0614, found 316.0649.

((*E*)-3-(*N*-((*E*)-4-Methoxy-4-oxobut-2-en-1-yl)phenylsulfonamido)prop-1-en-1-yl)boronic acid pinacol ester (3d)

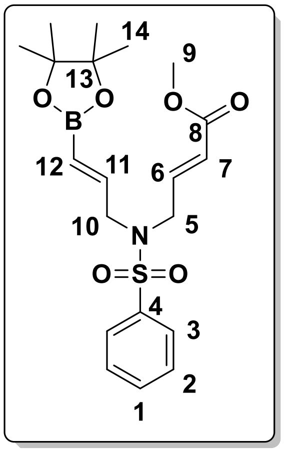

Prepared according to General Procedure A, **S12** (1.64 g, 5.6 mmol, 1 equiv) was converted to **3d**, yielding a white solid (344 mg, 0.8 mmol, 14%) after purification by flash column chromatography (SiO2, 0→15% EtOAc/n-hexane).

R*f* (20% EtOAc/*n*-hexane) = 0.19; **^{1}H NMR** (400 MHz, $CDCl_3$): δ = 7.81 (dd, *J* = 7.1, 1.6 Hz, 2H, H3), 7.61 – 7.55 (m, 1H, H1), 7.54 – 7.47 (m, 2H, H2), 6.67 (dt, *J* = 15.7, 5.8 Hz, 1H, H6), 6.35 (dt, *J* = 18.0, 5.8 Hz, 1H, H11), 5.87 (dt, *J* = 15.8, 1.6 Hz, 1H, H7), 5.52 (dt, *J* = 17.9, 1.5 Hz, 1H, H12), 3.95 (dd, *J* = 5.9, 1.6 Hz, 2H, H5), 3.88 (dd, *J* = 5.8, 1.5 Hz, 2H, H10), 3.71 (d, *J* = 0.9 Hz, 3H, H9), 1.24 (s, 12H, H14) ppm; **^{13}C NMR** (101 MHz, $CDCl_3$): δ = 166.1 (C8), 146.1 (C11), 142.3 (C6), 140.1 (C4), 132.9 (C1), 129.4 (C2), 127.4 (C3), 123.9 (C7), 83.6 (C13), 51.8 (C9), 51.5 (C10), 47.8 (C5), 25.0 (C14), 24.9 (C14) ppm; **^{11}B NMR** (128 MHz, $CDCl_3$) δ = 29.67 ppm; **IR** (ATR): 2978, 2949, 1723, 1660, 1647, 1447, 1439, 1413, 1403, 1383, 1372, 1364, 1322, 1309, 1301, 1275, 1203, 1193, 1171, 1157, 1140, 1095, 1021, 999, 991, 973, 963, 923, 912, 894, 851, 828, 782, 765, 752, 744, 735, 726, 686, 675, 668, 635, 619 cm^{-1}; **HRMS** (ESI) calc. For $C_{20}H_{28}BNO_6SNa^+$ $[M+Na]^+$ 444.1622, found 444.1695.

4,4-Diethyl 1,7-dimethyl (1*E*,6*E*)-hepta-1,6-diene-1,4,4,7-tetracarboxylate (3e)

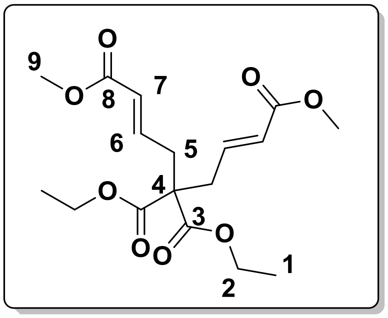

To an oven-dried round-bottomed flask was added NaH 60% in mineral oil (144 mg, 6 mmol, 3 equiv) before being sealed and placed under an argon atmosphere. To the flask was added anhydrous THF (6 mL, 1 M) and the reaction mixture cooled to 0 °C before the dropwise addition of diethyl malonate (0.3 mL, 2 mmol, 1 equiv). The reaction mixture was stirred at 0 °C for 30 minutes before the dropwise addition of methyl (*E*)-4-bromobut-2-enoate (0.6 mL, 5 mmol, 2.5 equiv). The reaction mixture was gradually warmed to room temperature and stirred for 18 h. After completion, the reaction mixture was quenched with sat. aq. NH_4Cl and extracted with ethyl acetate (3 × 10 mL). The combined organic phases were washed with brine, dried over Na_2SO_4, filtered and concentrated under reduced pressure. The resulting crude material was purified via flash column chromatography (SiO_2, 0→15% EtOAc/*n*-hexane) to yield **3e** as a colorless oil (525 mg, 1.5 mmol, 74%).

R*f* (20% EtOAc/*n*-hexane) = 0.30; **^{1}H NMR** (400 MHz, $CDCl_3$): δ = 6.78 (dt, *J* = 15.4, 7.7 Hz, 2H, H6), 5.89 (dt, *J* = 15.5, 1.4 Hz, 2H, H7), 4.22 (q, *J* = 7.1 Hz, 4H, H2), 3.73 (s, 6H, H9), 2.77 (dd, *J* = 7.7, 1.5 Hz, 4H, H5), 1.26 (t, *J* = 7.1 Hz, 6H, H1) ppm; **^{13}C NMR** (100 MHz, $CDCl_3$): δ = 169.7 (C3), 166.3 (C8), 142.2 (C6), 125.2 (C7), 62.1 (C2), 56.8 (C4), 51.8 (C9), 35.8 (C5), 14.2 (C1) ppm; **IR** (ATR): 2983, 2953, 1720, 1660, 1437, 1368, 1271, 1166, 1095, 1035, 981, 925, 893, 856, 725, 619 cm^{-1}; **HRMS** (ESI) calc. For $C_{17}H_{24}O_8Na^+$ $[M+Na]^+$ 379.1363, found 379.1413.

Diethyl 2,2-di(prop-2-yn-1-yl)malonate (S13)

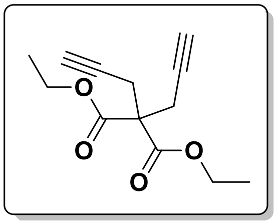

S13 was prepared according to a procedure of von Wangelin et al.[20] To an oven-dried round-bottomed flask was added NaH 60% in mineral oil (0.8 g, 20 mmol, 2 equiv) before being sealed and placed under an argon atmosphere. To the flask was added anhydrous THF (10 mL, 2 M) and the reaction mixture cooled to 0 °C before the dropwise addition of diethyl malonate (1.5 mL, 10 mmol, 1 equiv). The reaction mixture was stirred at 0 °C for 2 hours before the dropwise addition of propargyl bromide (2.6 mL, 25 mmol, 2.5 equiv). The reaction mixture was gradually warmed to room temperature and stirred for 18 h. After completion, the reaction mixture was quenched with sat. aq. NH_4Cl and extracted with ethyl acetate (3 × 10 mL). The combined organic phases were washed with brine, dried over Na_2SO_4, filtered and concentrated under reduced pressure. The resulting crude material was purified via flash column chromatography (SiO_2, 0→2% EtOAc/*n*-hexane) to yield **S13** as a colorless oil (1.79 g, 7.6 mmol, 76%).

^{1}H NMR (400 MHz, $CDCl_3$): δ = 4.23 (q, *J* = 7.1 Hz, 4H), 3.00 (d, *J* = 2.7 Hz, 4H), 2.03 (t, *J* = 2.6 Hz, 2H), 1.26 (t, *J* = 7.1 Hz, 6H) ppm; analytical data in agreement with literature.[20]

((1*E*,6*E*)-4,4-Bis(ethoxycarbonyl)hepta-1,6-diene-1,7-diyl)diboronic acid pinacol ester (3f)

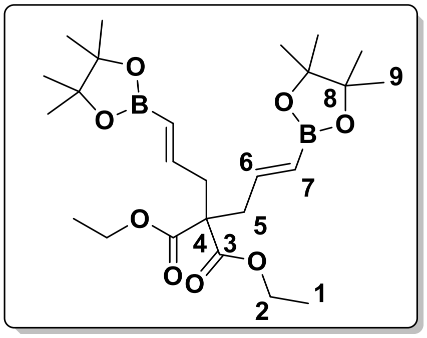

3f was prepared according to a modified version of General Procedure A, in which the reaction was ran with 2.2 equiv of 4,4,5,5-tetramethyl-1,3,2-dioxaborolane and 20 mol % Schwartz reagent. **S13** (553 mg, 2.3 mmol, 1 equiv) was converted to **3f** yielding a colorless oil (908 mg, 1.9 mmol, 79% yield) after purification by flash column chromatography (SiO_2, 0→10% EtOAc/*n*-hexane).

R*f* (10% EtOAc/*n*-hexane) = 0.15; **^{1}H NMR** (400 MHz, $CDCl_3$): δ = 6.41 (dt, *J* = 17.5, 7.2 Hz, 2H, H6), 5.56 – 5.47 (m, 2H, H7), 4.17 (q, *J* = 7.2 Hz, 4H, H2), 2.74 (d, *J* = 7.2 Hz, 4H, H5), 1.26 – 1.22 (m, 30H, H9, H1) ppm; **^{13}C NMR** (100 MHz, $CDCl_3$): δ = 170.6 (C3), 147.1 (C6), 83.3 (C8), 61.5 (C2), 57.2 (C4), 39.0 (C5), 25.0 (C9), 24.9 (C9), 14.2 (C1) ppm; **^{11}B NMR** (128 MHz, $CDCl_3$) δ = 29.89 ppm; **IR** (ATR): 2979, 1731, 1638, 1446, 1391, 1380, 1360, 1321, 1285, 1259, 1216, 1183, 1166, 1143, 1113, 1095, 1031, 999, 969, 848, 754, 668, 649 cm^{-1}; **HRMS** (ESI) calc. for $C_{25}H_{42}B_2O_8Na^+$ $[M+Na]^+$ 515.2958, found 515.2896.

((1*E*,1'*E*)-((Phenylsulfonyl)azanediyl)bis(prop-1-ene-3,1-diyl))diboronic acid pinacol ester (3g)

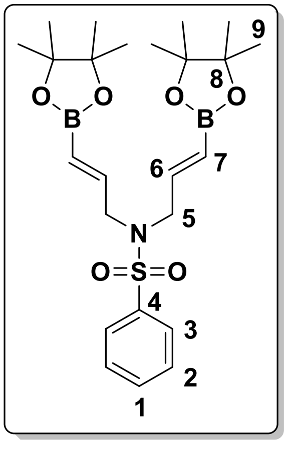

3g was prepared according to a modified version of General Procedure A, in which the reaction was ran with 2.2 equiv of 4,4,5,5-tetramethyl-1,3,2-dioxaborolane and 20 mol % Schwartz reagent. **S11** (1.17 g, 5 mmol, 1 equiv) was converted to **3g** yielding a white solid (1.09 g, 2.2 mmol, 44% yield) after purification by flash column chromatography (SiO_2, 0→20% EtOAc/*n*-hexane).

R*f* (20% EtOAc/*n*-hexane) = 0.32; **^{1}H NMR** (400 MHz, $CDCl_3$): δ = 7.83 – 7.78 (m, 2H, H3), 7.58 – 7.51 (m, 1H, H1), 7.51 – 7.45 (m, 2H, H2), 6.34 (dt, *J* = 17.9, 5.7 Hz, 2H, H6), 5.50 (dt, *J* = 17.8, 1.5 Hz, 2H, H7), 3.89 (dd, *J* = 5.7, 1.5 Hz, 4H, H5), 1.24 (s, 24H, H9) ppm; **^{13}C NMR** (101 MHz, $CDCl_3$): δ = 146.5 (C6), 140.5 (C4), 132.6 (C1), 129.1 (C2), 127.4 (C3), 83.5 (C8), 50.9 (C5), 24.9 (C9) ppm; **^{11}B NMR** (128 MHz, $CDCl_3$) δ = 29.68 ppm; **IR** (ATR): 2976, 2933,

1740, 1644, 1474, 1447, 1391, 1380, 1361, 1344, 1316, 1265, 1213, 1143, 1091, 1055, 1005, 972, 950, 922, 887, 848, 831, 805, 764, 747, 719, 706, 686, 668, 622 cm^{-1}; **HRMS** (ESI) calc. For $C_{24}H_{37}B_2NO_6SNa^+$ $[M+Na]^+$ 512.2420, found 512.2480.

Diethyl 2-(3-methylbut-2-en-1-yl)malonate (S14)

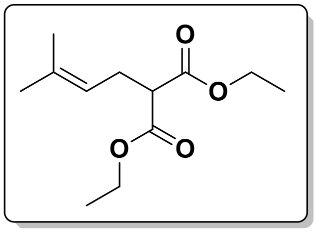

To an oven-dried round-bottomed flask was added K_2CO_3 (2.73 g, 20 mmol, 2 equiv). The flask was sealed and purged with nitrogen before the sequential addition of anhydrous acetone (50 mL, 0.2 M) and diethyl malonate (1.53 mL, 10 mmol, 1 equiv). The reaction mixture was cooled to 0 °C and 1-bromo-3-methyl-2-butene (1.28 mL, 11 mmol, 1.1 equiv) was added dropwise. The reaction mixture was stirred at room temperature for 18 h. After completion, the reaction was quenched with sat. aq. NH_4Cl (30 mL) and extracted with ethyl acetate (3 × 50 mL). The combined organic phases were washed with brine, dried over Na_2SO_4, filtered and concentrated under reduced pressure. The resulting crude was purified via flash column chromatography (SiO_2, 0→2% EtOAc/*n*-hexane) to yield **S14** as a colorless oil (1.64 g, 7.2 mmol, 72%).

^{1}H NMR (400 MHz, $CDCl_3$) δ = 5.06 (dddd, *J* = 7.4, 5.9, 2.9, 1.5 Hz, 1H), 4.18 (qd, *J* = 7.2, 1.6 Hz, 4H), 3.32 (td, *J* = 7.7, 1.6 Hz, 1H), 2.68 – 2.34 (m, 2H), 1.67 (t, *J* = 1.4 Hz, 3H), 1.63 (d, *J* = 1.8 Hz, 3H), 1.37 – 1.16 (m, 6H) ppm; analytical data in agreement with literature.[21]

Diethyl 2-(3-methylbut-2-en-1-yl)-2-(prop-2-yn-1-yl)malonate (S15)

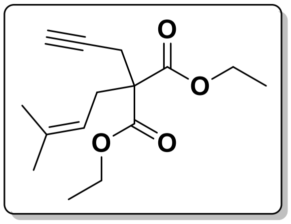

To an oven-dried round-bottomed flask was added NaH (60% in paraffin oil, 350 mg, 8.75 mmol, 1.25 equiv) before being sealed and placed under nitrogen atmosphere. To the flask was added anhydrous THF (25 mL) via syringe and the reaction mixture was cooled to 0 °C before the dropwise addition of **S14** (1.60 g, 7 mmol, 1 equiv). The reaction mixture was stirred at room temperature for 10 minutes before the dropwise addition of propargyl bromide (0.76 mL, 8.4 mmol, 1.2 equiv) at 0 °C. The reaction mixture was stirred at room temperature for 5 h. After completion, the reaction was quenched with sat. aq. NH_4Cl (20 mL) and extracted with ethyl acetate (3 × 30 mL). The combined organic phases were washed with brine, dried over Na_2SO_4, filtered and concentrated under reduced pressure. The resulting crude was purified via flash column chromatography (SiO_2, 0→20% EtOAc/*n*-hexane) to yield **S15** as a yellow oil (1.67 g, 6.3 mmol, 90%).

^{1}H NMR (400 MHz, $CDCl_3$) δ = 4.91 (ddq, *J* = 8.0, 6.6, 1.7 Hz, 1H), 4.30 – 4.08 (m, 4H), 2.84 – 2.70 (m, 4H), 2.04 – 1.91 (m, 1H), 1.69 (s, 3H), 1.66 (s, 3H), 1.25 (td, *J* = 7.1, 1.4 Hz, 6H) ppm; analytical data in agreement with literature.[22]

(*E*)-(4,4-Bis(ethoxycarbonyl)-7-methylocta-1,6-dien-1-yl)boronic acid pinacol ester (S16)

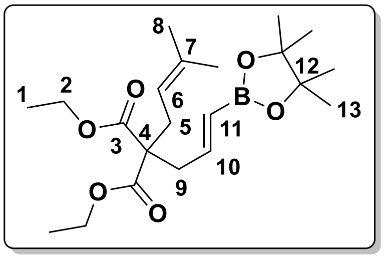

Prepared according to General Procedure A, **S15** (799 mg, 3 mmol, 1 equiv) was converted to **S16**, yielding a colorless oil (924 mg, 2.3 mmol, 78%) after purification by flash column chromatography (SiO_2, 0→10% EtOAc/n-hexane).

R_f (20% EtOAc/*n*-hexane) = 0.27; **^{1}H NMR** (400 MHz, $CDCl_3$) δ = 6.42 (ddd, *J* = 18.0, 8.2, 6.4 Hz, 1H, H10), 5.54 – 5.44 (m, 1H, H11), 4.97

(ddd, *J* = 9.1, 6.7, 2.1 Hz, 1H, H6), 4.28 – 4.03 (m, 4H, H2), 2.73 (dd, *J* = 7.2, 1.5 Hz, 2H, H9), 2.59 (d, *J* = 7.4 Hz, 2H, H5), 1.67 (d, *J* = 1.9 Hz, 3H, H8), 1.59 (d, *J* = 1.6 Hz, 3H, H8), 1.25 – 1.20 (m, 18H, H1+H13) ppm; **^{13}C NMR** (100 MHz, $CDCl_3$) δ = 171.2 (C3), 147.7 (C10), 135.6 (C7), 117.8 (C6), 83.3 (C12), 61.3 (C2), 57.6 (C4), 39.1 (C9), 31.3 (C5), 26.1 (C8), 24.9 (C13), 18.2 (C8), 14.2 (C1) ppm; **^{11}B NMR** (128 MHz, $CDCl_3$) δ = 29.66 ppm; **IR** (ATR): 2979, 2933, 1730, 1639, 1446, 1361, 1321, 1283, 1258, 1225, 1216, 1166, 1166, 1144, 1113, 1095, 1065, 1025, 1002, 970, 848, 777, 652; **HRMS** (ESI) calc. for $C_{21}H_{35}BO_6Na^+$ $[M+Na]^+$ 417.2419, found 417.2406.

Experimental setup for photoreactions

The photocatalyzed energy transfer reactions yielding cyclobutane boronic esters were carried out using LG Innotek UV SMD-LED 3535, model LEUVA35T01RL01 (365 nm) LEDs. The LEDs were placed 0.5 cm away from the reaction vial. To regulate temperature, a fan was used for cooling.

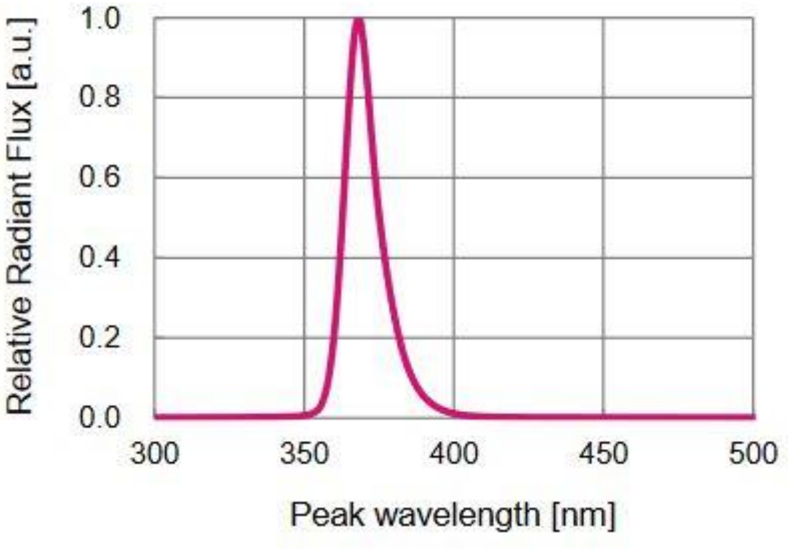

Figure S1. Emission spectra of LG Innotek UV SMD-LED 3535, model LEUVA35T01RL01.[23]

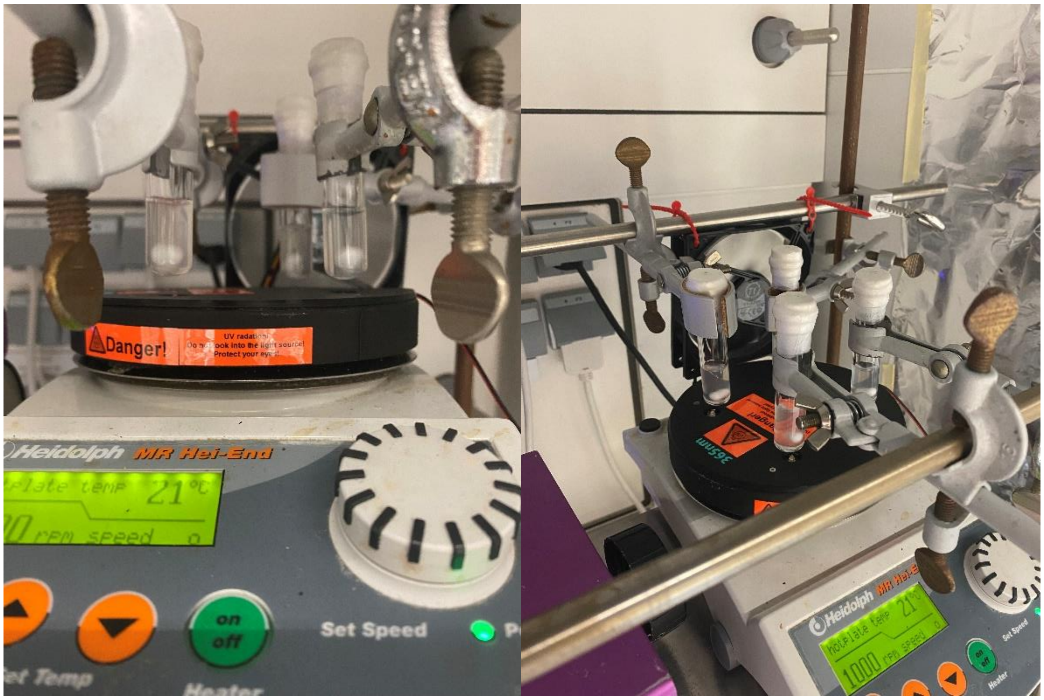

Figure S2. Reaction set up for photcatalyzed energy transfer reaction. (Left: side view, right: diagonal view)

Reaction probe: alkene Isomerization via energy transfer

Table S1. Photocatalyst screening

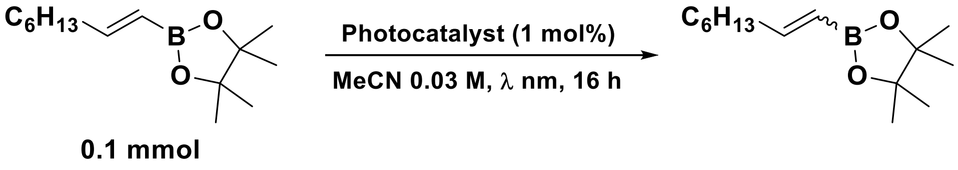

Entry	Catalyst	Light Source (nm)	$E_{1/2}(M^*/M^+)$ (V)[24]	E_T (Kcal/mol)[24]	Yield[a] (%)	*E*:*Z* ratio[a]
1[b]	Xanthone	365	-1.65	74.2	83	73:27
2[b]	Thioxanthone	365	-1.62	65.5	81	89:11
3	$(Ir[dF(CF_3)ppy]_2(dtbpy))PF_6$	450	-0.89	60.1	92	96:4
4[b]	4CzIPN	450	-1.04	58.3	99	95:5
5	Ir(*p*-CF_3)$_3$	450	-1.695	56.4	92	>99:1
6	$Ir(ppy)_3$	450	-1.73	55.2	92	>99:1
7	$(Ir[Me(Me)ppy]_2(dtbpy))PF_6$	450	-0.87	47.9	97	97:3
8[b]	Eosin Y	450	-1.08	45.4	89	>99:1
9	$[Ru(dmbpy)_3](PF_6)_2$	450	-0.51	45.3	91	>99:1

[a] Determined by ^{1}H NMR spectroscopy against a known internal standard (1,3,5-trimethoxybenzene). [b] 5 mol% cat. loading.

Table S2. Solvent screening

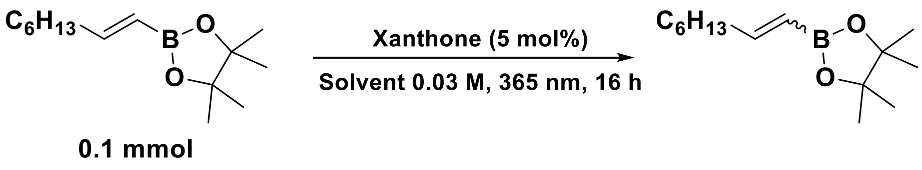

Entry	Solvent	Yield[a] (%)	*E*:*Z* ratio[a]
1	MeCN	83	73:27
2	EtOAc	92	78:22
3	DMF	86	>99:1
4	DCM	80	75:25
5	MeOH	73	85:15
6	THF	86	>99:1
7	Toluene	92	97:3
8	Cyclohexane	91	95:5

[a] Determined by ^{1}H NMR spectroscopy against a known internal standard (1,3,5-trimethoxybenzene).

Table S3. Boron ligand screening

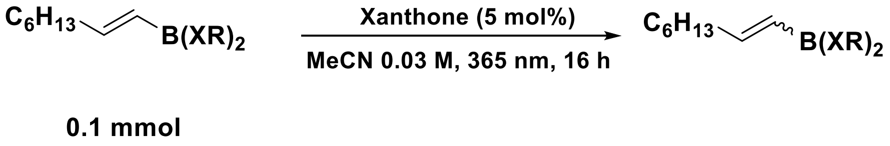

Entry	Ligand	Yield[a] (%)	*E*:*Z* ratio[a]
1	Pin	83	73:27
2	Cat	94	66:34
3	Neo-Pentyl	76	76:24
4	Benzopinacol	71	80:20
5	MIDA	90	90:10
6	DAN	80	>95:5

[a] Determined by ^1H NMR spectroscopy against a known internal standard (1,3,5-trimethoxybenzene).

Reaction optimization: [2 + 2] Cycloaddition

Table S4. Photocatalyst loading screening

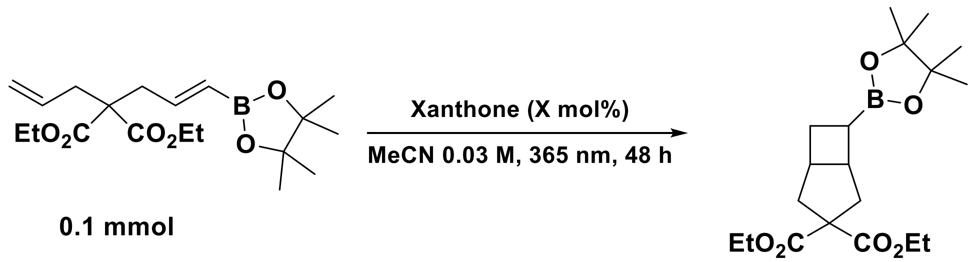

Entry	Photocatalyst Loading (mol%)	Yield[a] (%)	SM Remaining[a] (%)
1	5	8	81
2	10	10	77
3	20	15	60
4	50	24	49

[a] Determined by ^{1}H NMR spectroscopy against a known internal standard (1,3,5-trimethoxybenzene).

Table S5. Effect of alkene substitution

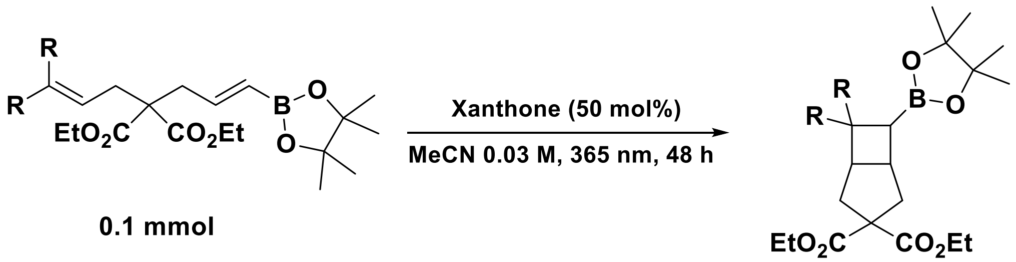

Entry	R	Yield[a] (%)	SM Remaining[a] (%)
1	H	24	49
2	Me	9	70

[a] Determined by ^{1}H NMR spectroscopy against a known internal standard (1,3,5-trimethoxybenzene).

Table S6. Photocatalyst screening

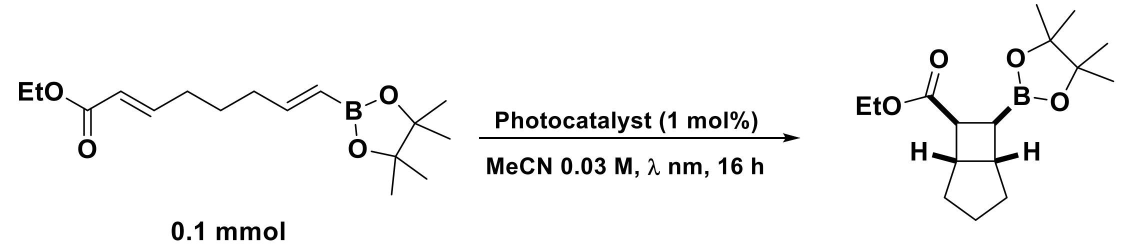

Entry	Catalyst	Light Source (nm)	$E_{1/2}$(M^*/M^+) (V)[24]	E_T (Kcal/mol)[24-25]	Yield[a] (%)	SM Remaining[a] (%)
1[b]	Xanthone	365	-1.65	74.2	76[c]	12
2[d]	Xanthone	365	-1.65	74.2	35[e]	30
3[f]	[Au(SIPr)(Cbz)]	365	-	66.6	50[g]	21
4[h]	[Au(SIPr)(Cbz)]	365	-	66.6	17[g]	71
5[i]	[Au(SIPr)(Cbz)]	365	-	66.6	8[g]	87
6[d]	Thioxanthone	400	-1.62	65.5	10[j]	45
7	(Ir[dF(Me)ppy]$_2$(dtbbpy))PF_6	450	-0.92	62.9	0	83
8	(Ir[dF(CF_3)ppy]$_2$(dtbpy))PF_6	450	-0.89	60.1	0	64
9[d]	4CzIPN	450	-1.04	58.3	0	80
10	Ir(*p*-CF_3)$_3$	450	-1.695	56.4	0	65

[a] Determined by ^{1}H NMR spectroscopy against a known internal standard (1,3,5-trimethoxybenzene). [b] 20 mol% cat. loading. [c] d.r. = 4.6:1. [d] 5 mol% cat. loading. [e] d.r. = 6:1. [f] 6 mol% cat. loading. [g] d.r. = >20:1. [h] 4 mol% cat. loading. [i] 2 mol% cat. loading. [j] d.r. = 9:1.

Table S7. Photocatalyst loading screening

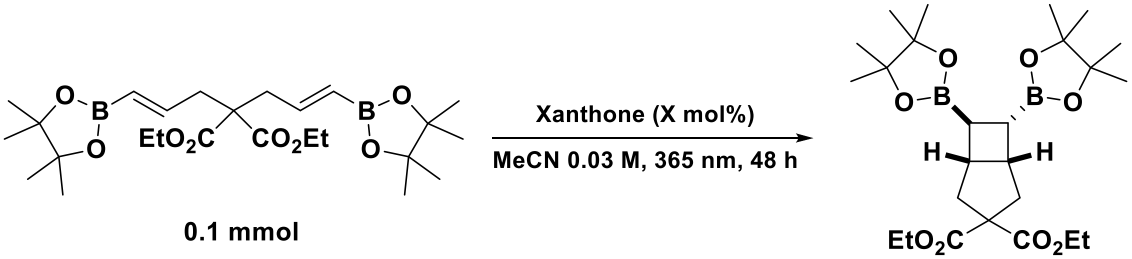

Entry	Photocatalyst Loading (mol%)	Yield[a] (%)	d.r.	SM Remaining[a] (%)
1	10	21	1.5:1	52
2	15	34	1.1:1	40
3	20	42	1.1:1	32
4	50	69	1.3:1	13

[a] Determined by ^{1}H NMR spectroscopy against a known internal standard (1,3,5-trimethoxybenzene).

Substrate scope

General Procedure B: Photocatalyzed intramolecular [2 + 2] of vinylboron pinacol esters and vinyl esters

To an oven-dried 5 mL microwave vial, the specified vinylboronate ester (1 equiv) and xanthone (20 mol %) were added. The vial was sealed with a septum and purged with nitrogen before the addition of degassed MeCN (0.03 M). The reaction mixture was stirred under light irradiation (365 nm) for 16 h. After completion, internal standard (1,3,5-trimethoxybenzene), as a solution in MeCN, was added and the reaction was washed with sat. aq. K_2CO_3 (10 mL). Organics were extracted with EtOAc (3 × 10 mL). The combined organic phases were dried over Na_2SO_4, filtered and concentrated under reduced pressure. The crude residue was purified by flash column chromatography ($B(OH)_3$-SiO_2 or SiO_2, specified combination of solvents). Borylated products purified by SiO_2 were exposed to the minimum amount of SiO_2 for as little time as possible to limit degradation.

General Procedure C: Photocatalyzed intramolecular [2 + 2] of vinylboron pinacol esters

To an oven-dried 5 mL microwave vial, the specified vinyl boronate ester (1 equiv) and xanthone (50 mol %) were added. The vial was sealed with a septum and purged with nitrogen before the addition of degassed MeCN (0.03 M). The reaction mixture was stirred under light irradiation (365 nm) for 48 h. After completion, internal standard (1,3,5-trimethoxybenzene), as a solution in MeCN, was added and the reaction was washed with sat. aq. K_2CO_3 (10 mL). Organics were extracted with EtOAc (3 × 10 mL). The combined organic phases were dried over Na_2SO_4, filtered and concentrated under reduced pressure. The crude residue was immediately used in an oxidation reaction (General Procedure D) to aid in separation and characterization.

General Procedure D: Oxidation of boronic acid pinacol esters with H_2O_2

Prepared according to a modified procedure of Yu et al.[26] The crude residue generated following General Procedure C was redissolved in THF (1.5 mL) and aqueous NaH_2PO_4 solution (0.5 M, 1.5 mL) before the dropwise addition of H_2O_2 (30 wt % in H_2O, 0.75 mL) at 0 °C. The reaction mixture was warmed to room temperature and stirred for 3 hours. After completion, the reaction mixture was quenched with H_2O, and extracted with EtOAc (3 × 10 mL). The combined organic layers were washed with brine, dried over Na_2SO_4, filtered and concentrated under reduced pressure. The crude residue was purified via flash column chromatography. (SiO_2, specified combination of solvents).

Spectral data is reported for the major diastereomer in the purified products unless stated otherwise.

(7-(Ethoxycarbonyl)bicyclo[3.2.0]heptan-6-yl)boronic acid pinacol ester (4b)

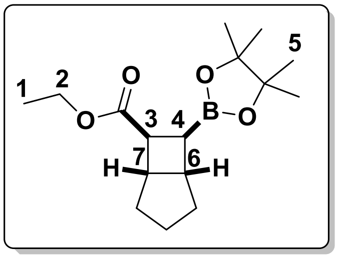

Prepared according to General Procedure B, **3b** (117.7 mg, 0.4 mmol) was converted to **4b** yielding a colorless oil (76% NMR yield d.r. = 4.6:1, 62.5 mg, 53%, d.r. = >20:1), after purification by flash column chromatography ($B(OH)_3$-SiO_2, 0→15% EtOAc/*n*-hexane).

R*f* (10% EtOAc/*n*-hexane) = 0.56; **^{1}H NMR** (400 MHz, $CDCl_3$): δ = 4.20 – 4.03 (m, 2H, H2), 2.95 – 2.86 (m, 1H, H7), 2.85 – 2.78 (m, 1H, H6), 2.69 (dd, *J* = 11.0, 4.6 Hz, 1H, H3), 1.92 – 1.78 (m, 2H), 1.62 (dd, *J* = 13.0, 6.1 Hz, 1H), 1.58 – 1.45 (m, 4H, H4 and 3 alkyl C-H's), 1.28 – 1.22 (m, 15H, H1 and H5) ppm; **^{13}C NMR** (101 MHz, $CDCl_3$): δ = 176.5, 83.4, 60.4, 42.7, 41.5, 37.0, 33.8, 33.0, 25.1, 25.0, 24.8, 14.4 ppm; **^{11}B NMR** (128

MHz, $CDCl_3$) δ = 33.27 ppm; **IR** (ATR): 2976, 2934, 1724, 1461, 1447, 1407, 1370, 1345, 1312, 1263, 1240, 1163, 1143, 1097, 1074, 1052, 1025, 981, 919, 863, 848, 731, 689, 669, 647 cm^{-1}; **HRMS** (ESI) calc. for $C_{16}H_{27}BO_4Na^+$ $[M+Na]^+$ 317.1894, found 317.1897. Relative stereochemistry of major diastereomer assigned through analogy with compounds **4c** and **4d**.

(3,3-Bis(ethoxycarbonyl)-7-(methoxycarbonyl)bicyclo[3.2.0]heptan-6-yl)boronic acid pinacol ester (4c)

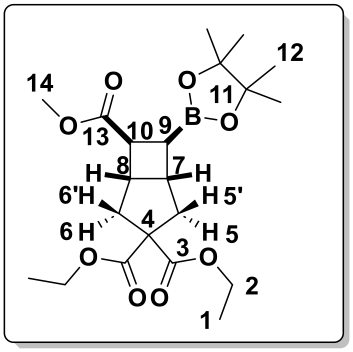

Prepared according to General Procedure B, **3c** (84.8 mg, 0.2 mmol) was converted to **4c** yielding a colorless oil (81% NMR yield, d.r. = 3:1, 59.4 mg, 70%, d.r. = 3:1), after purification by flash column chromatography ($B(OH)_3$-SiO_2, 0→15% EtOAc/*n*-hexane).

R*f* (20% EtOAc/*n*-hexane) = 0.40; **^{1}H NMR** (400 MHz, $CDCl_3$): δ = 4.23 (q, *J* = 7.1 Hz, 2H, H2), 4.15 (q, *J* = 7.1 Hz, 2H, H2), 3.65 (s, 3H, H14), 3.12 – 3.02 (m, 1H, H8), 2.96 (dd, *J* = 10.9, 5.3 Hz, 1H, H10), 2.93 – 2.86 (m, 1H, H7), 2.50 – 2.43 (m, 1H, H5’), 2.43 – 2.38 (m, 2H, H6 and H6’), 2.34 (dd, *J* = 14.0, 3.7 Hz, 1H, H5), 1.82 – 1.75 (m, 1H, H9), 1.30 – 1.19 (m, 18H, H1 and H12) ppm; **^{13}C NMR** (101 MHz, $CDCl_3$): δ = 176.2 (C13), 172.4 (C3), 172.3 (C3), 83.6 (C11), 63.7 (C4), 61.7 (C2), 51.7 (C14), 42.9 (C10), 41.93 (C5), 41.89 (C8), 40.7 (C6), 37.7 (C7), 25.1 (C12), 25.0 (C12), 14.2 (C1), 14.1 (C1) ppm; **^{11}B NMR** (128 MHz, $CDCl_3$) δ = 33.70 ppm; **IR** (ATR): 2979, 1727, 1436, 1408, 1371, 1321, 1249, 1194, 1167, 1141, 1055, 1024, 978, 925, 857, 794, 755, 668 cm^{-1}; **HRMS** (ESI) calc. for $C_{21}H_{33}BO_8Na^+$ $[M+Na]^+$ 447.2160, found 447.2117.

(7-(Methoxycarbonyl)-3-(phenylsulfonyl)-3-azabicyclo[3.2.0]heptan-6-yl)boronic acid pinacol ester (4d)

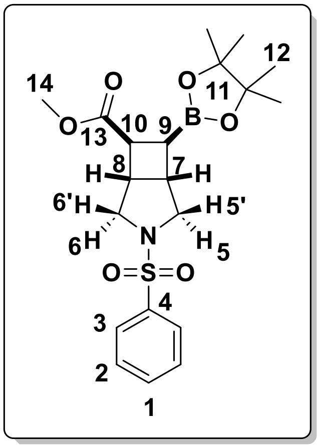

Prepared according to General Procedure B, **3d** (42.1 mg, 0.2 mmol) was converted to **4d** yielding a colorless oil (72% NMR yield d.r. = 4.1:1, 54.9 mg, 65%, d.r. = 3.2:1), after purification by flash column chromatography ($B(OH)_3$-SiO_2, 0→20% EtOAc/*n*-hexane).

R*f* (20% EtOAc/*n*-hexane) = 0.23; **^{1}H NMR** (400 MHz, $CDCl_3$): δ = 7.84 – 7.79 (m, 2H, H3), 7.64 – 7.58 (m, 1H, H1), 7.57 – 7.50 (m, 2H, H2), 3.66 (s, 3H, H14), 3.53 (d, *J* = 10.1 Hz, 1H, H6), 3.46 (d, *J* = 9.8 Hz, 1H, H5), 3.12 (dd, *J* = 11.0, 4.9 Hz, 1H, H10), 3.07 – 3.00 (m, 1H, H8), 2.89 (q, *J* = 6.6 Hz, 1H, H7), 2.68 – 2.60 (m, 2H, H5’ and H6’), 2.01 (dd, *J* = 11.1, 5.6 Hz, 1H, H9), 1.23 (d, *J* = 5.7 Hz, 12H, H12) ppm; **^{13}C NMR** (101 MHz, $CDCl_3$): δ = 175.5 (C13), 135.2 (C4), 133.0 (C1), 129.1 (C2), 128.1 (C3), 83.8 (C11), 55.0 (C5), 54.0 (C6), 51.8 (C13), 41.9 (C10), 40.4 (C8), 36.6 (C7), 25.1 (C12), 25.0 (C12) ppm; **^{11}B NMR** (128 MHz, $CDCl_3$) δ = 33.22 ppm; **IR** (ATR): 1713, 1446, 1368, 1341, 1327, 1174, 1160, 1144, 1094, 1051, 1008, 998, 979, 856, 838, 811, 764, 718, 695, 675, 606 cm^{-1}; **HRMS** (ESI) calc. for $C_{20}H_{28}BNO_6SNa^+$ $[M+Na]^+$ 444.1622, found 444.1679.

3,3-Diethyl 6,7-dimethyl bicyclo[3.2.0]heptane-3,3,6,7-tetracarboxylate (4e)

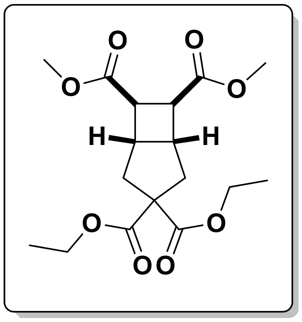

Prepared according to General Procedure B, **3e** (142.4 mg, 0.4 mmol) was converted to **4e** yielding a colorless oil (86% NMR yield d.r. = 2.3:1, 106.3 mg, 75%, d.r. = 3.3:1), after purification by flash column chromatography (SiO_2, 0→15% EtOAc/*n*-hexane).

R*f* (20% EtOAc/*n*-hexane) = 0.22; **(Mixture of diastereomers)** **^{1}H NMR** (400 MHz, $CDCl_3$): δ = 4.28 – 4.21 (m, 2.3H), 4.21 – 4.11 (m, 3.2H), 3.69 (d, *J* = 2.1 Hz, 1.9H), 3.66 (s, 6H), 3.47 (td, *J* = 9.2, 1.7 Hz, 0.3H), 3.25 – 3.21 (m, 0.3H), 3.21 – 3.15 (m, 2H), 3.11 – 3.06 (m, 1.9H), 3.01 (q, *J* = 8.4 Hz, 0.3H), 2.94-2.85 (td, 0.3H), 2.57 – 2.46 (m, 0.8H), 2.46 – 2.41 (m, 3.6H), 2.31 – 2.19 (m, 0.6H), 1.32 – 1.19 (m, 8.6H) ppm;

(major diastereomer - *syn*) **^{13}C NMR** (101 MHz, $CDCl_3$): δ = 173.4, 172.4, 171.7, 63.5, 61.9, 61.9, 52.0, 43.8, 40.1, 40.0, 14.2, 14.1 ppm;

(minor diastereomer - *anti*) **^{13}C NMR** (101 MHz, $CDCl_3$): δ = 174.0, 172.4, 171.9, 171.3, 63.9, 61.8, 52.2, 52.0, 45.0, 40.3, 40.0, 39.2, 38.9, 36.4, 14.1 ppm; analytical data in agreement with literature.[15]

(3,3-Bis(ethoxycarbonyl)bicyclo[3.2.0]heptane-6,7-diyl)diboronic acid, pinacol ester (4f)

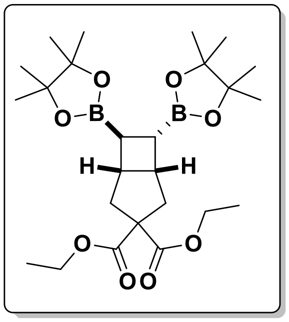

Prepared according to General Procedure C, **3f** (147.7 mg, 0.3 mmol) was converted to **4f** with a 69% NMR yield (d.r. = 1.3:1).

The crude reaction mixture was subsequently oxidized to aid with isolation from residual starting material and characterization of the major isomer.

Diethyl 6,7-dihydroxybicyclo[3.2.0]heptane-3,3-dicarboxylate (S17)

Prepared according to General Procedure D, **4f** was converted to **S17** yielding a pale yellow oil (26.5 mg, 32%, d.r. = 1.5:1) after purification by flash column chromatography (SiO_2, 0→100% Et_2O/*n*-pentane).

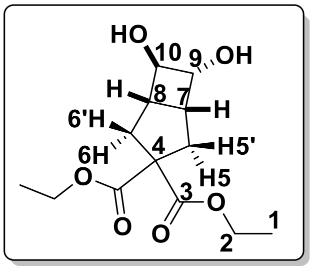

R*f* (100% Et_2O) = 0.19; **^{1}H NMR** (400 MHz, $CDCl_3$): δ = 4.29 – 4.10 (m, 4H, H2), 4.01 – 3.92 (m, 1H, H9), 3.78 (s, 1H, OH), 3.64 (t, *J* = 5.3 Hz, 1H, H10), 3.17 (s, 1H, OH), 2.95 (qd, *J* = 9.0, 5.0 Hz, 1H, H7), 2.60 (dd, *J* = 14.2, 8.0 Hz, 1H, H6'), 2.53 (dd, *J* = 14.7, 5.0 Hz, 1H, H5), 2.34 – 2.25 (m, 2H, H8 and H6), 2.09 (dd, *J* = 14.7, 9.8 Hz, 1H, H5'), 1.28 – 1.20 (m, 6H, H1) ppm; **^{13}C NMR** (101 MHz, $CDCl_3$): δ = 174.0 (C3), 171.9 (C3), 81.1 (C10), 72.5 (C9), 63.7 (C4), 62.2 (C2), 61.8 (C2), 42.2 (C8), 39.4 (C7), 39.1 (C6), 33.0 (C5), 14.12 (C1), 14.08 (C1) ppm; **IR** (ATR): 3382, 1721, 1367, 1250, 1183, 1062, 860, 761, 734 cm^{-1}; **HRMS** (ESI) calc. for $C_{13}H_{20}O_6Na^+$ $[M+Na]^+$ 295.1152, found 295.1159.

(3-(Phenylsulfonyl)-3-azabicyclo[3.2.0]heptane-6,7-diyl)diboronic acid, pinacol ester (4g)

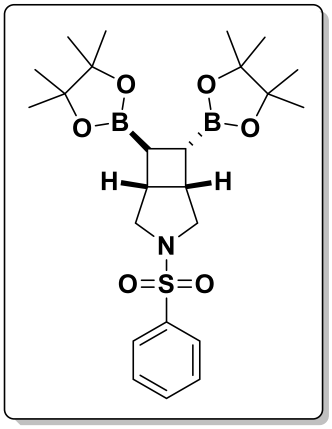

Prepared according to General Procedure C, **3g** (146.8 mg, 0.3 mmol) was converted to **4g** with a 58% NMR yield (d.r. = 1.4:1).

The crude reaction mixture was subsequently oxidized to aid with isolation from residual starting material and characterization of the major isomer.

3-(Phenylsulfonyl)-3-azabicyclo[3.2.0]heptane-6,7-diol

Prepared according to General Procedure D, **4g** was converted to **S18** yielding an off-white solid (39.6 mg, 50%, d.r. = 1.6:1) after purification by flash column chromatography (SiO_2, 0→2% MeOH/DCM).

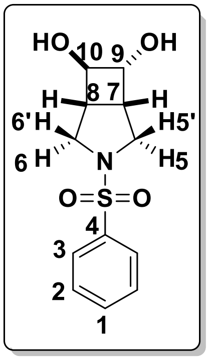

(S18-*anti*, major diastereomer) R*f* (10% MeOH/DCM) = 0.21; **^{1}H NMR** (400 MHz, MeOD): δ = 7.86 – 7.82 (m, 2H, H3), 7.71 – 7.66 (m, 1H, H1), 7.65 – 7.59 (m, 2H, H2), 4.00 – 3.92 (m, 1H, H9), 3.77 (t, *J* = 5.8 Hz, 1H, H10), 3.71 (d, *J* = 8.4 Hz, 1H, H5), 3.47 (d, *J* = 9.6 Hz, 1H, H6), 2.83 – 2.69 (m, 3H, H6', H5' and H7), 2.31 – 2.23 (m, 1H, H8) ppm; **^{13}C NMR** (101 MHz, MeOD): δ = 136.6 (C4), 134.2 (C1), 130.3 (C2), 129.0 (C3), 78.5 (C10), 72.4 (C9), 53.2 (C6), 46.6 (C5), 42.1 (C7), 38.6 (C8) ppm; **IR** (ATR): 3329, 1447, 1335, 1164, 1121, 1093, 949, 808, 754, 719, 688, 625 cm^{-1}; **HRMS** (ESI) calc. for $C_{12}H_{15}NO_4SNa^+$ $[M+Na]^+$ 292.0614, found 292.0595.

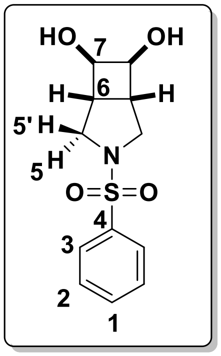

(S18-*syn*, minor diastereomer) R*f* (10% MeOH/DCM) = 0.28; **^{1}H NMR** (400 MHz, MeOD): δ = 7.84 – 7.80 (m, 2H, H3), 7.72 – 7.66 (m, 1H, H1), 7.65 – 7.59 (m, 2H, H2), 3.98 – 3.91 (m, 2H, H7), 3.48 (d, *J* = 9.2 Hz, 2H, H5), 2.69 – 2.59 (m, 4H, H5' and H6) ppm; **^{13}C NMR** (101 MHz, MeOD): δ = 136.0 (C4), 134.3 (C1), 130.3 (C2), 129.1 (C3), 72.5 (C7), 53.0 (C5), 45.9 (C6) ppm; **IR** (ATR): 3298, 2923, 2854, 1446, 1338, 1316, 1169, 1008, 909, 805, 784, 755, 719, 689, 612 cm^{-1}; **HRMS** (ESI) calc. for $C_{12}H_{15}NO_4SNa^+$ $[M+Na]^+$ 292.0614, found 292.0591.

(3,3-Bis(ethoxycarbonyl)-6-(methoxycarbonyl)bicyclo[3.2.0]heptane-1,7-diyl)diboronic acid pinacol ester (4h)

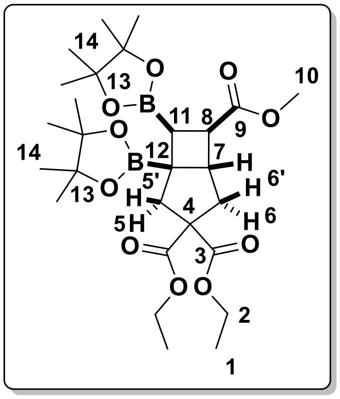

According to General Procedure B, **3h** was converted to **4h** yielding a colorless oil (69% NMR yield, d.r. = 1.4:1, 29.8 mg, 27%, d.r. = 10:1), after purification by flash column chromatography ($B(OH)_3$-SiO_2, 0→10% EtOAc/*n*-hexane).

R_f (20% EtOAc/*n*-hexane) = 0.18; **^{1}H NMR** (400 MHz, $CDCl_3$) δ = 4.20 (tq, *J* = 7.1, 3.7 Hz, 2H, H2), 4.13 (q, *J* = 7.2 Hz, 2H, H2), 3.63 (s, 3H, H10), 3.10 (q, *J* = 5.5 Hz, 1H, H7), 3.00 (dd, *J* = 10.8, 5.1 Hz, 1H, H8), 2.59 (d, *J* = 14.3 Hz, 1H, H5), 2.49 – 2.43 (m, 3H, H5'+H6'+H6), 1.95 (d, *J* = 10.7 Hz, 1H, H11), 1.22 (m, 30H, H1 and H14); **^{13}C NMR** (101 MHz, $CDCl_3$) δ = 175.6 (C9), 172.6 (C3), 172.1 (C3), 83.4 (C13), 64.21 (C4), 61.5 (C2), 61.4 (C2), 51.4 (C10), 45.7 (C5), 45.0 (C7), 42.4 (C8), 40.8 (C6), 25.18 (C14), 25.16 (C14), 25.1 (C14), 24.7 (C14), 14.1 (C1), 14.0 (C1); **^{11}B NMR** (128 MHz, $CDCl_3$) δ = 33.55 ppm; **IR** (ATR): 2979, 2936, 1727, 1446, 1367, 1314, 1246, 1212, 1193, 1167, 1141, 1095, 1070, 972, 916, 854, 731, 669, 646; **HRMS** (ESI) calc. for $C_{27}H_{44}B_2O_{10}Na^+$ $[M+Na]^+$ 573.3013, found 573.3041.

Mechanistic studies

Control reactions

Control reactions (isomerization)

All reactions were performed following a modified version of General Procedure B, with the described deviations.

Table S8. Control reactions for energy eransfer catalyzed isomerization.

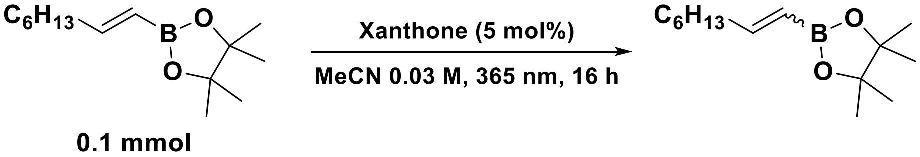

Entry	Deviation	Yield[a] (%)	*E*:*Z* Ratio[a]
1	No Photocatalyst	92	>99:1
2	No Light	93	>99:1
3	Presence of O_2	59	73:27

[a] Determined by ^{1}H NMR spectroscopy against a known internal standard (1,3,5-trimethoxybenzene).

*Reaction of Z-Alkene Z-*1a

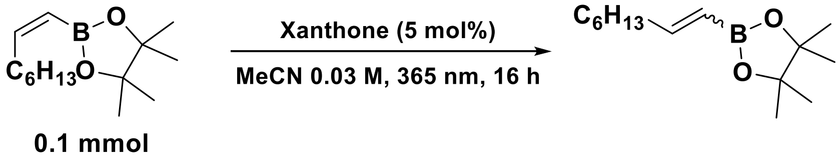

Reaction was performed according to General Procedure B, with ***Z*-1a** as starting material and 5 mol % of xanthone. After completion, internal standard (1,3,5-trimethoxybenzene), as a solution in MeCN, was added. Crude NMR analysis against the internal standard revealed isomerization of ***Z*-1a** had proceeded (87%, 71:29 *E*:*Z*).

Reaction of 3f with Au Photocatalyst

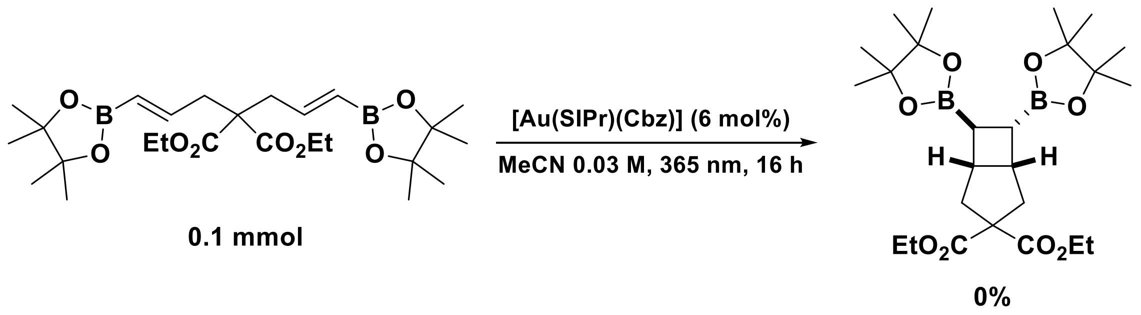

Reaction was performed according to a modified version of General Procedure B, with **3f** as starting material and 6 mol % of [Au(SIPr)(Cbz)]. After completion, internal standard (1,3,5-trimethoxybenzene), as a solution in MeCN, was added. Crude NMR analysis against the internal standard revealed no formation of product had occurred. 87% of **3f** was retained.

Comment: This result combined with entry 3 of Table S6 supports preferential activation of vinyl esters over vinyl BPin motifs through use of [Au(SIPr)(Cbz)] photocatalyst.

Intermolecular [2 + 2] control reactions

Reaction with styrene

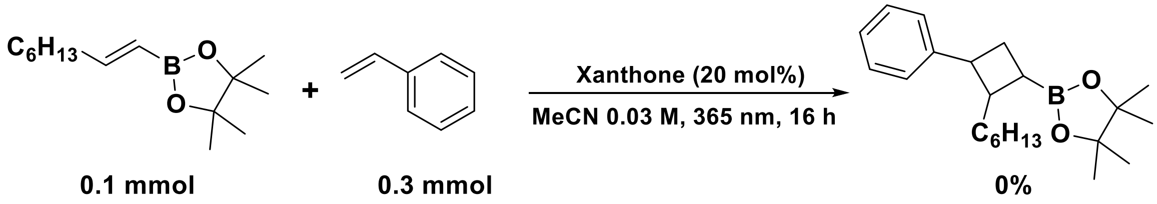

Reaction was performed according to a modified version of General Procedure B, with the addition of styrene (3 equiv). After completion, internal standard (1,3,5-trimethoxybenzene), as a solution in MeCN, was added. Crude NMR analysis against the internal standard revealed no formation of product had occurred. 92% of **1a** (85:15, *E*:*Z*) was retained.

Reaction with methyl acrylate

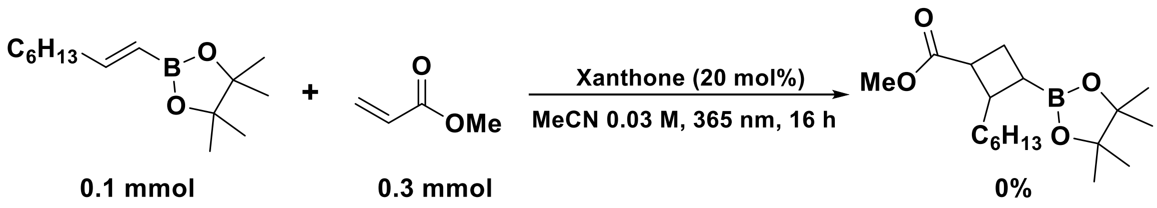

Reaction was performed according to a modified version of General Procedure B, with the addition of methyl acrylate (3 equiv). After completion, internal standard (1,3,5-trimethoxybenzene), as a solution in MeCN, was added. Crude NMR analysis against the internal standard revealed no formation of product had occurred. 87% of **1a** (72:28, *E*:*Z*) was retained.

Reaction with allyl alcohol

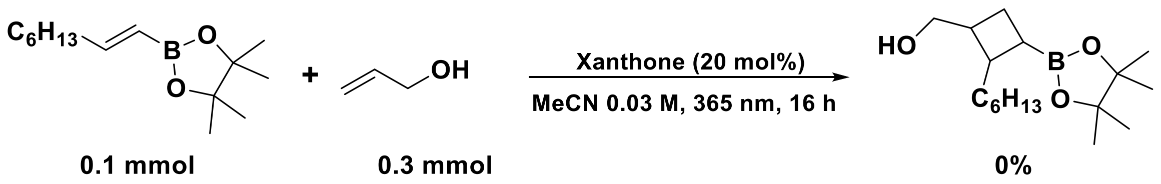

Reaction was performed according to a modified version of General Procedure B, with the addition of allyl alcohol (3 equiv). After completion, internal standard (1,3,5-trimethoxybenzene), as a solution in MeCN, was added. Crude NMR analysis against the internal standard revealed no formation of product had occurred. 85% of **1a** (72:28, *E*:*Z*) was retained.

Reaction with allyl alcohol via temporary coordination

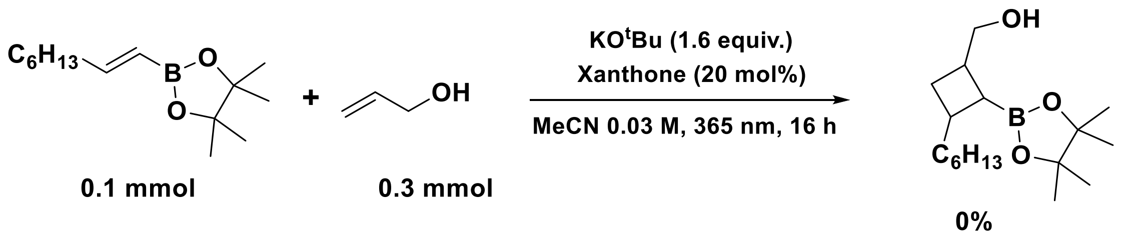

Reaction was performed according to modified procedure of Brown et al.[27] To an oven-dried 5 mL microwave vial, the specified vinyl boronate ester (1 equiv), potassium *tert*-butoxide (1.6 equiv), and xanthone (20 mol %) were added. The vial was sealed with a septum and purged with nitrogen before the addition of degassed MeCN (0.03 M) and allyl alcohol (3 equiv). The reaction mixture was stirred under light irradiation (365 nm) for 16 h. After completion, internal standard (1,3,5-trimethoxybenzene), as a solution in MeCN, was added. Crude NMR analysis against the internal standard revealed no formation of product had occurred. 100% of ***E*-1a** (>99:1, *E*:*Z*) was retained.

Cyclic voltammetry studies

Cyclic voltammetry was conducted on an Interface 1000 Gamry potentiostat using a 3-electrode cell configuration. A glassy carbon working electrode was employed alongside a platinum wire counter electrode and an Ag/Ag^{+} reference electrode. The solution was degassed by bubbling nitrogen prior to measurements. The analysis was carried out on a 5 mM solution in MeCN along with 0.1 M of tetrabutylammonium hexafluorophosphate as supporting electrolyte and with 5 mM of ferrocene as internal standard. It was examined at a scan rate of 0.05 V s^{-1}.

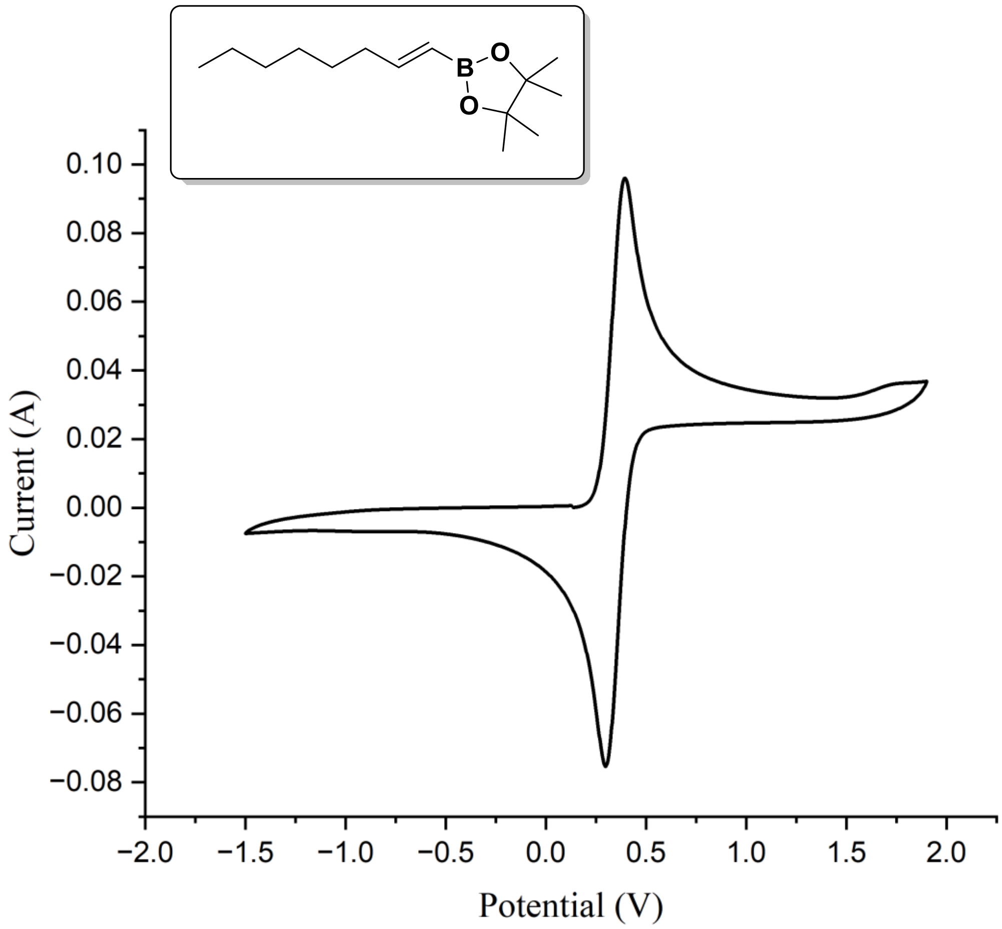

Figure S3. CV of ***E*-1a**

Comment: The potential was scanned between 1.93 V vs SCE and −1.47 V vs SCE, spanning the redox window from 1.57 V vs SCE to −1.42 V vs SCE. No specific redox peak was observed, indicating that the triplet excited state xanthone does not undergo a single electron transfer under these conditions.

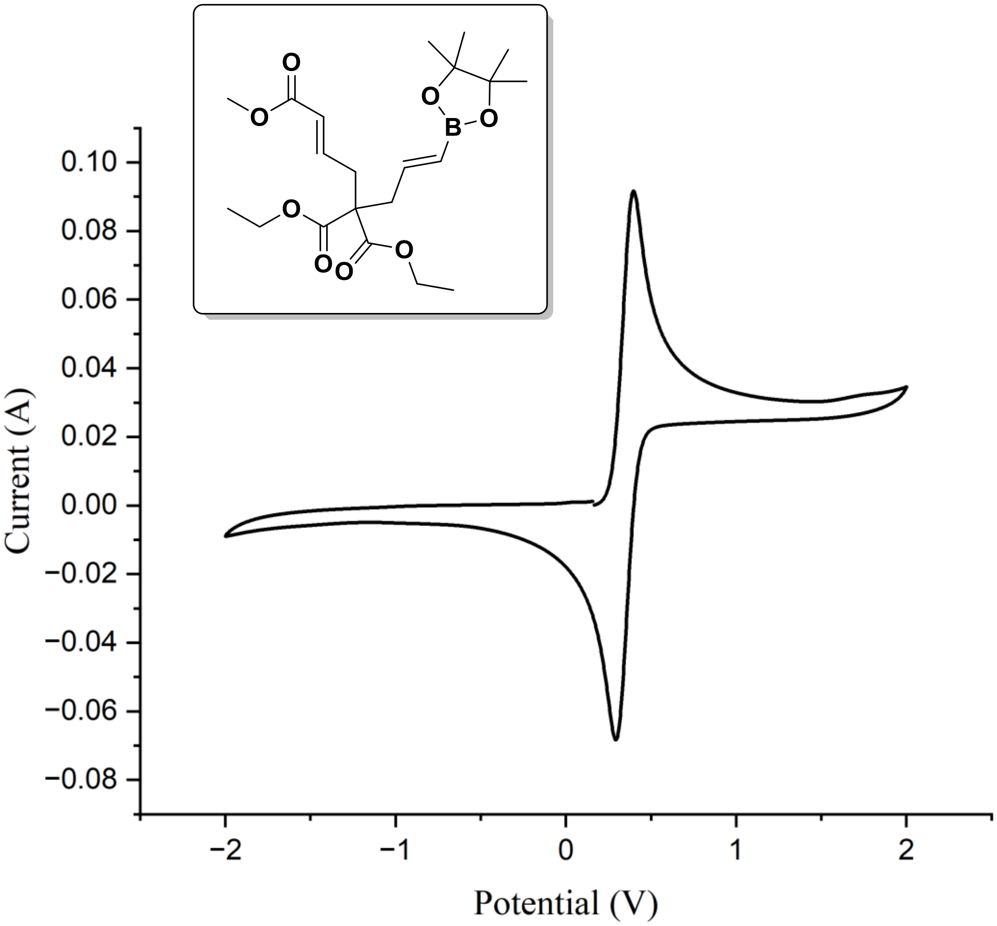

Figure S4. CV of **3c**

Comment: The potential was scanned between 2.03 V vs SCE and −1.97 V vs SCE, spanning the redox window from 1.57 V vs SCE to −1.42 V vs SCE. No specific redox peak was observed, indicating that the triplet excited state xanthone does not undergo a single electron transfer under these conditions.

UV–vis analysis

All absorption spectra were recorded on a Shimadzu UV-1900 I UV–vis Spectrophotometer, at medium speed with 2 nm steps in the 330–600 nm range using a 1 cm path quartz cuvette. All concentrations are based on the concentration of model reaction conditions unless stated otherwise.

Absorption spectra of starting materials

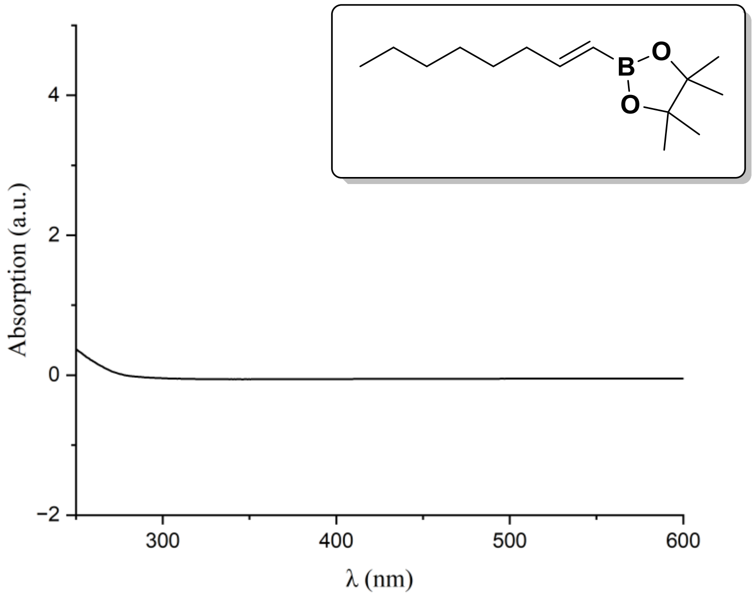

Figure S5. Absorption spectrum of ***E*-1a** in MeCN (0.03 M).

Comment: The absorption spectra of sample ***E*-1a** at reaction concentration showed no absorption bands above 260 nm.

Derivatization

Methyl 7-hydroxy-3-(phenylsulfonyl)-3-azabicyclo[3.2.0]heptane-6-carboxylate (5)

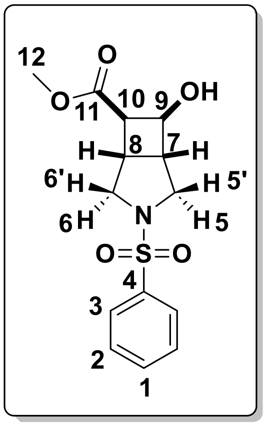

5 was prepared according to a modified procedure of Yu et al.[26] To a microwave vial was added **4d** (84 mg, 0.2 mmol, 1 equiv) before being sealed and placed under an argon atmosphere. To the flask was added THF (1 mL) and aqueous NaH_2PO_4 solution (0.5 M, 1 mL) before the dropwise addition of H_2O_2 (30 wt % in H_2O, 0.5 mL) at 0 °C. The reaction mixture was warmed to room temperature and stirred for 3 hours. After completion, the reaction mixture was quenched with H_2O, and extracted with EtOAc (3 × 10 mL). The combined organic layers were washed with brine, dried over Na_2SO_4, filtered and concentrated under reduced pressure. The resulting crude material was purified via flash column chromatography (SiO_2, 0→60% EtOAc/*n*-hexane) to yield **5** as a white solid (37 mg, 0.12 mmol, 60%, *d.r.* = 3:1).

$\mathbf{R_f}$ (50% EtOAc/*n*-hexane) = 0.22; **^{1}H NMR** (400 MHz, $CDCl_3$): δ = 7.82 – 7.77 (m, 2H, H3), 7.65 – 7.59 (m, 1H, H1), 7.59 – 7.52 (m, 2H, H2), 4.33 (td, *J* = 6.5, 3.3 Hz, 1H, H9), 3.72 (s, 3H, H12), 3.60 (d, *J* = 10.1 Hz, 1H, H5), 3.49 (d, *J* = 10.1 Hz, 1H, H6), 3.23 – 3.16 (m, 1H, H10), 3.14 – 3.05 (m, 1H, H8), 2.80 – 2.72 (m, 1H, H7), 2.62 (ddd, *J* = 16.2, 10.0, 6.5 Hz, 2H, H6 and H5) ppm; **^{13}C NMR** (101 MHz, $CDCl_3$): δ = 172.7 (C11), 134.6 (C4), 133.2 (C1), 129.2 (C2), 128.1 (C3), 70.0 (C9), 53.0 (C6), 52.3 (C5), 52.2 (C12), 47.7 (C10), 46.8 (C7), 36.0 (C8) ppm; **IR** (ATR): 3433, 1729, 1718, 1447, 1436, 1337, 1167, 1151, 1090, 1008, 998, 804, 757, 718, 688, 640, 606 cm^{-1}; **HRMS** (ESI) calc. for $C_{14}H_{17}NO_5SNa^+$ $[M+Na]^+$ 334.0719, found 334.0706.

Potassium (7-(ethoxycarbonyl)bicyclo[3.2.0]heptan-6-yl)trifluoroborate (6)

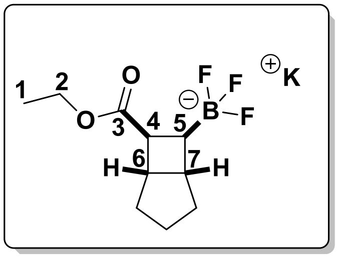

6 was prepared according to a modified procedure of Yoon et al.[8] To a microwave vial was added **4b** (29 mg, 0.1 mmol, 1 equiv) before being sealed and placed under an argon atmosphere. To the flask was added MeCN (0.5 mL) and MeOH (0.5 mL), before the addition of KF (23 mg, 0.4 mmol, 4 equiv) in H_2O (0.1 mL). The reaction mixture was stirred 10 minutes followed by the addition of L-tartaric acid (32 mg, 0.21 mmol, 2.1 equiv) in THF (0.5 mL). The reaction mixture was then stirred for a further 30 minutes. The reaction mixture was filtered through a sintered funnel and washed with MeCN (3 × 10 mL). The solvent was removed under reduced pressure to yield **6** as a white solid (27 mg, 0.1 mmol, quant. yield).

^{1}H NMR (400 MHz, DMSO-*d*$_6$): δ = 3.89 (qd, *J* = 7.1, 2.9 Hz, 2H, H2), 2.85 – 2.77 (m, 1H, H6), 2.40 – 2.32 (m, 1H, H7), 2.31 – 2.24 (m, 1H, H4), 1.86 – 1.71 (m, 1H), 1.70 – 1.61 (m, 1H), 1.39 – 1.28 (m, 4H), 1.11 (t, *J* = 7.1 Hz, 3H, H1), 1.01 – 0.90 (m, 1H, H5) ppm; **^{13}C NMR** (101 MHz, DMSO-*d*$_6$): δ 175.8 (C3), 58.5 (C2), 41.4 (C4), 37.6 (C6), 36.7 (C7), 34.1, 32.6, 24.9, 14.2 (C1) ppm; **^{11}B NMR** (128 MHz, DMSO-*d*$_6$) δ = 4.14 ppm; **^{19}F NMR** (376 MHz, DMSO-*d*$_6$) δ = -140.94 ppm; **IR** (ATR): 2934, 1713, 1163, 1094, 758, 718, 691 cm^{-1}; **HRMS** (ESI) calc. for $C_{10}H_{15}BF_3O_2^-$ $[M-K]^-$ 235.1123, found 235.1134.

Potassium (3,3-bis(ethoxycarbonyl)-7-(methoxycarbonyl)bicyclo[3.2.0]heptan-6-yl)trifluoroborate (7)

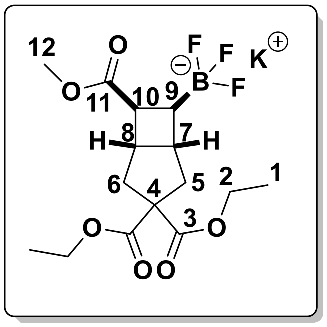

7 was prepared according to a modified procedure of Yoon et al.[8] To a microwave vial was added **4c** (121 mg, 0.28 mmol, 1 equiv) before being sealed and placed under an argon atmosphere. To the flask was added MeCN (1.5 mL) and MeOH (1.5 mL), before the addition of KF (65 mg, 1.1 mmol, 4 equiv) in H_2O (0.25 mL). The reaction mixture was stirred 10 minutes followed by the addition of (*L*)-tartaric acid (84 mg, 0.56 mmol, 2.1 equiv) in THF (1.2 mL). The reaction mixture was then stirred for a further 30 minutes. The reaction mixture was filtered through a sintered funnel and washed with MeCN (3 × 10 mL). The solvent was removed under reduced pressure to yield **7** as a white solid (97 mg, 0.24 mmol, 86%).

^{1}H NMR (400 MHz, DMSO-d_6): δ = 4.14 (q, *J* = 7.1 Hz, 2H, H2), 4.08 (q, *J* = 7.1 Hz, 2H, H2), 3.42 (s, 3H, H12), 2.94 – 2.84 (m, 1H, H8), 2.56 – 2.52 (m, 1H, H10), 2.43 – 2.35 (m, 1H, H7), 2.26 (dd, *J* = 13.3, 8.0 Hz, 1H, H5), 2.17 (dd, *J* = 13.6, 8.4 Hz, 1H, H6), 2.07 (dt, *J* = 13.4, 3.8 Hz, 2H, H5+6), 1.16 (dt, *J* = 15.8, 7.0 Hz, 6H, H1), 1.11 – 1.04 (m, 1H, H9) ppm; **^{13}C NMR** (101 MHz, DMSO-d_6): δ = 175.9 (C11), 171.9 (C3), 171.8 (C3), 63.6 (C4), 60.9 (C2), 60.8 (C2), 50.4 (C12), 42.2 (C10 + C5), 40.2 (C6, obscured by DMSO peak), 38.8 (C8), 37.7 (C7), 13.9 (C1), 13.9 (C1) ppm; **^{11}B NMR** (128 MHz, DMSO-d_6) δ = 8.62 ppm; **^{19}F NMR** (376 MHz, DMSO-d_6) δ = -136.87 ppm; **IR** (ATR): 2980, 1720, 1466, 1441, 1370, 1321, 1298, 1252, 1233, 1216, 1204, 1179, 1136, 1113, 1097, 1062, 1031, 996, 962, 920, 866, 781, 761, 732 cm^{-1}; **HRMS** (ESI) calc. for $C_{15}H_{21}BF_3K_2O_6^+$ [M+K]$^+$ 443.0651, found 443.0681.

References

[1] M. Musiejuk, J. Doroszuk and D. Witt, *RSC Advances* **2018**, *8*, 9718-9722.
[2] A. Bismuto, M. J. Cowley and S. P. Thomas, *Advanced Synthesis & Catalysis* **2021**, *363*, 2382-2385.
[3] J. Janetzko and R. A. Batey, *The Journal of Organic Chemistry* **2014**, *79*, 7415-7424.
[4] Z. Kong, W. Hu and J. P. Morken, *ACS Catalysis* **2023**, *13*, 11522-11527.
[5] Marek Zaidlewicz and Jerzy Melier, *Main Group Metal Chemistry* **2000**, *23*, 765-772.
[6] C. Yang, Y. Gao, S. Bai, C. Jiang and X. Qi, *Journal of the American Chemical Society* **2020**, *142*, 11506-11513.
[7] E. U. Brice, P. G. Eric and D. B. Martin, *Tetrahedron* **2009**, *65*, 3130-3138.
[8] S. O. Scholz, J. B. Kidd, L. Capaldo, N. E. Flikweert, R. M. Littlefield and T. P. Yoon, *Organic Letters* **2021**, *23*, 3496-3501.
[9] S. del Pozo, S. Vera, M. Oiarbide and C. Palomo, *Journal of the American Chemical Society* **2017**, *139*, 15308-15311.
[10] S. P. Pitre, J. C. Scaiano and T. P. Yoon, *ACS Catalysis* **2017**, *7*, 6440-6444.
[11] T. Schlatzer, J. Kriegesmann, H. Schröder, M. Trobe, C. Lembacher-Fadum, S. Santner, A. V. Kravchuk, C. F. W. Becker and R. Breinbauer, *Journal of the American Chemical Society* **2019**, *141*, 14931-14937.
[12] C. E. Tucker, B. Greve, W. Klein and P. Knochel, *Organometallics* **1994**, *13*, 94-101.
[13] H.-M. Huang, Q. He and D. J. Procter, *Synlett* **2020**, *31*, 45-50.
[14] K. Kaczanowska, B. Trzaskowski, A. Peszczyńska, A. Tracz, R. Gawin, T. K. Olszewski and K. Skowerski, *ChemCatChem* **2020**, *12*, 6366-6374.
[15] T. Yumura, T. Nanjo and Y. Takemoto, *ACS Catalysis* **2023**, *13*, 12803-12809.
[16] S. E. Denmark and J. H.-C. Liu, *Journal of the American Chemical Society* **2007**, *129*, 3737-3744.
[17] T. Ishiyama, N. Matsuda, N. Miyaura and A. Suzuki, *Journal of the American Chemical Society* **1993**, *115*, 11018-11019.
[18] F. Ziyanak, M. Kuş and L. Artok, *Advanced Synthesis & Catalysis* **2011**, *353*, 897-902.
[19] J. Teske and B. Plietker, *ACS Catalysis* **2016**, *6*, 7148-7151.
[20] D. Brenna, M. Villa, T. N. Gieshoff, F. Fischer, M. Hapke and A. Jacobi von Wangelin, *Angewandte Chemie International Edition* **2017**, *56*, 8451-8454.
[21] B. Yang and Z. Lu, *The Journal of Organic Chemistry* **2016**, *81*, 7288-7300.
[22] A. López-Rodríguez, G. Domínguez and J. Pérez-Castells, *The Journal of Organic Chemistry* **2019**, *84*, 924-933.
[23] LG Innotek Specifications for Approval. https://lumstatic.com/pF/RJ/zUmB547UfBKXaoGDsQ.pdf (accessed November 2024).
[24] a) K. Teegardin, J. I. Day, J. Chan and J. Weaver, *Organic Process Research & Development* **2016**, *20*, 1156-1163; b) N. A. Romero and D. A. Nicewicz, *Chemical Reviews* **2016**, *116*, 10075-10166; c) T. Nevesely, M. Wienhold, J. J. Molloy and R. Gilmour, *Chemical Reviews* **2022**, *122*, 2650-2694; d) T.-Y. Shang, L.-H. Lu, Z. Cao, Y. Liu, W.-M. He and B. Yu, *Chemical Communications* **2019**, *55*, 5408-5419; e) B. Happ, C. Friebe, A. Winter, M. D. Hager, R. Hoogenboom and U. S. Schubert, *Chemistry – An Asian Journal* **2009**, *4*, 154-163.
[25] E. A. Martynova, V. A. Voloshkin, S. G. Guillet, F. Bru, M. Beliš, K. Van Hecke, C. S. J. Cazin and S. P. Nolan, *Chemical Science* **2022**, *13*, 6852-6857.
[26] J. He, Q. Shao, Q. Wu and J.-Q. Yu, *Journal of the American Chemical Society* **2017**, *139*, 3344-3347.
[27] Y. Liu, D. Ni and M. K. Brown, *Journal of the American Chemical Society* **2022**, *144*, 18790-18796.

NMR Spectra

^{1}H NMR of S1 (400 MHz, CD_3OD)

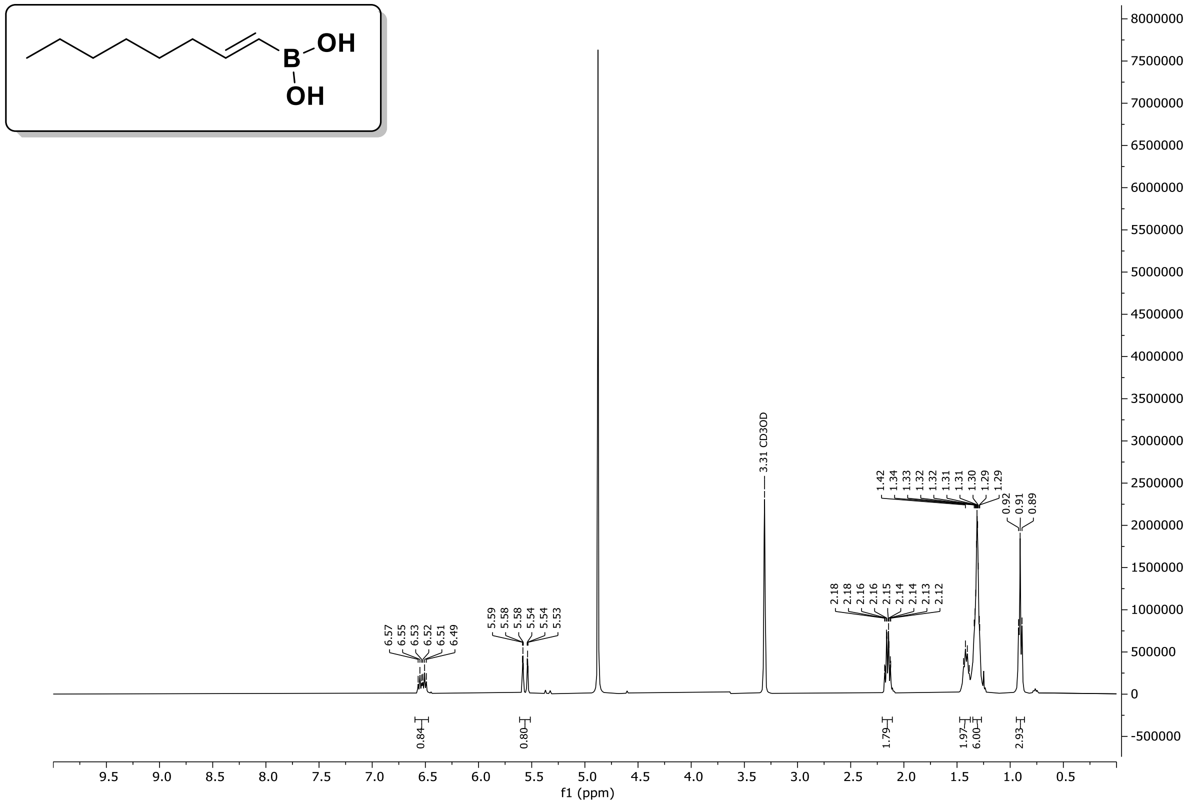

^{1}H NMR of *E*-1a (400 MHz, $CDCl_3$)

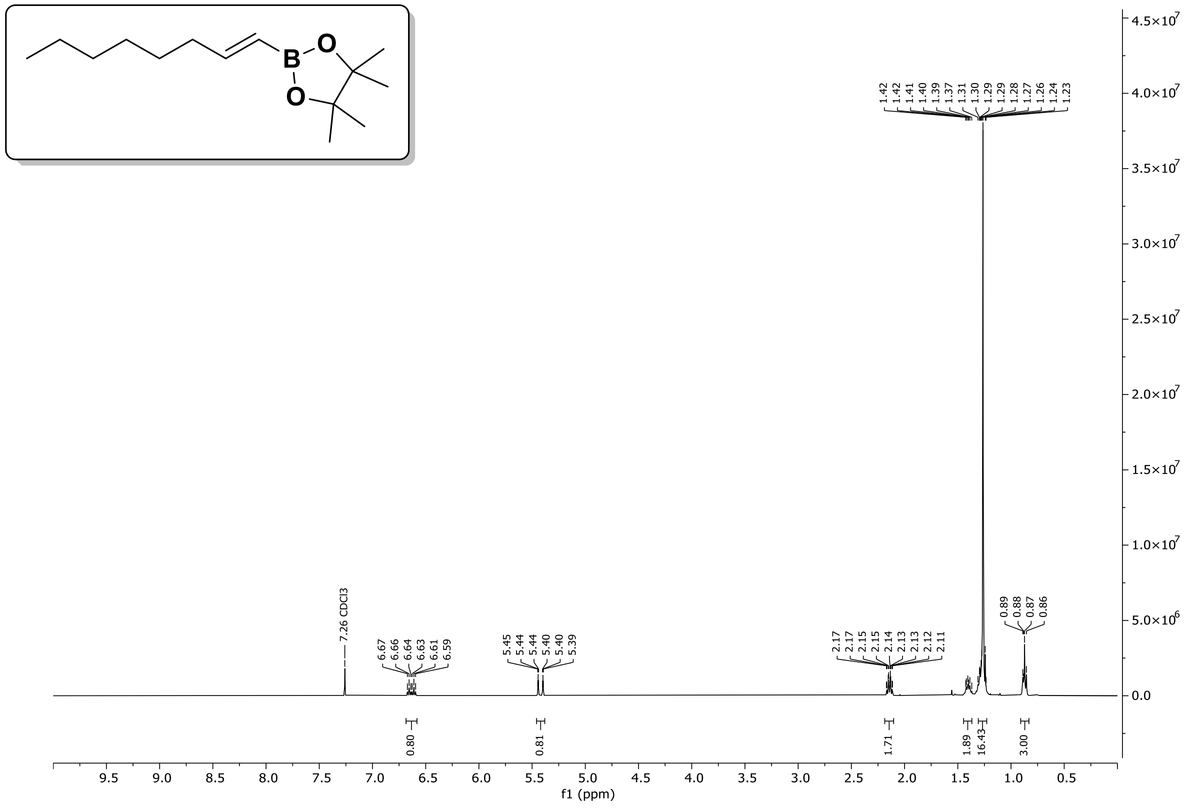

^{1}H NMR of S2 (400 MHz, $CDCl_3$)

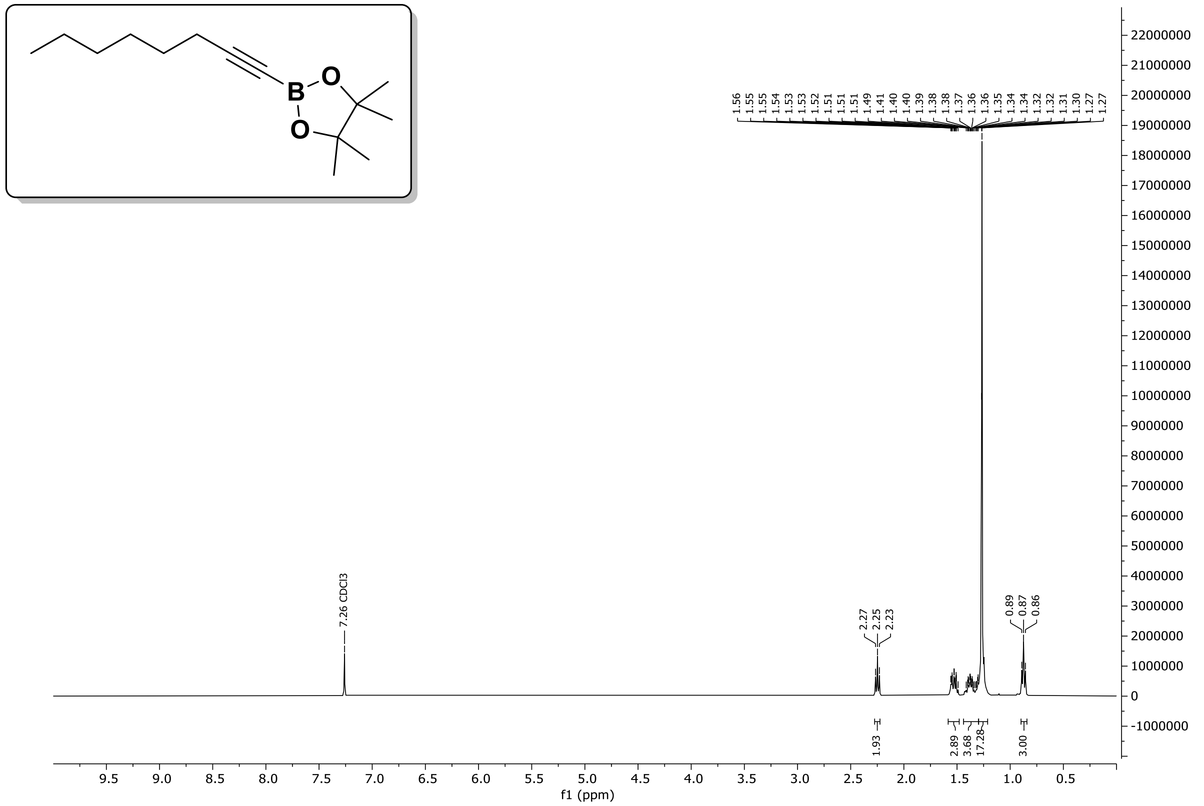

^{1}H NMR of *Z*-1a (400 MHz, $CDCl_3$)

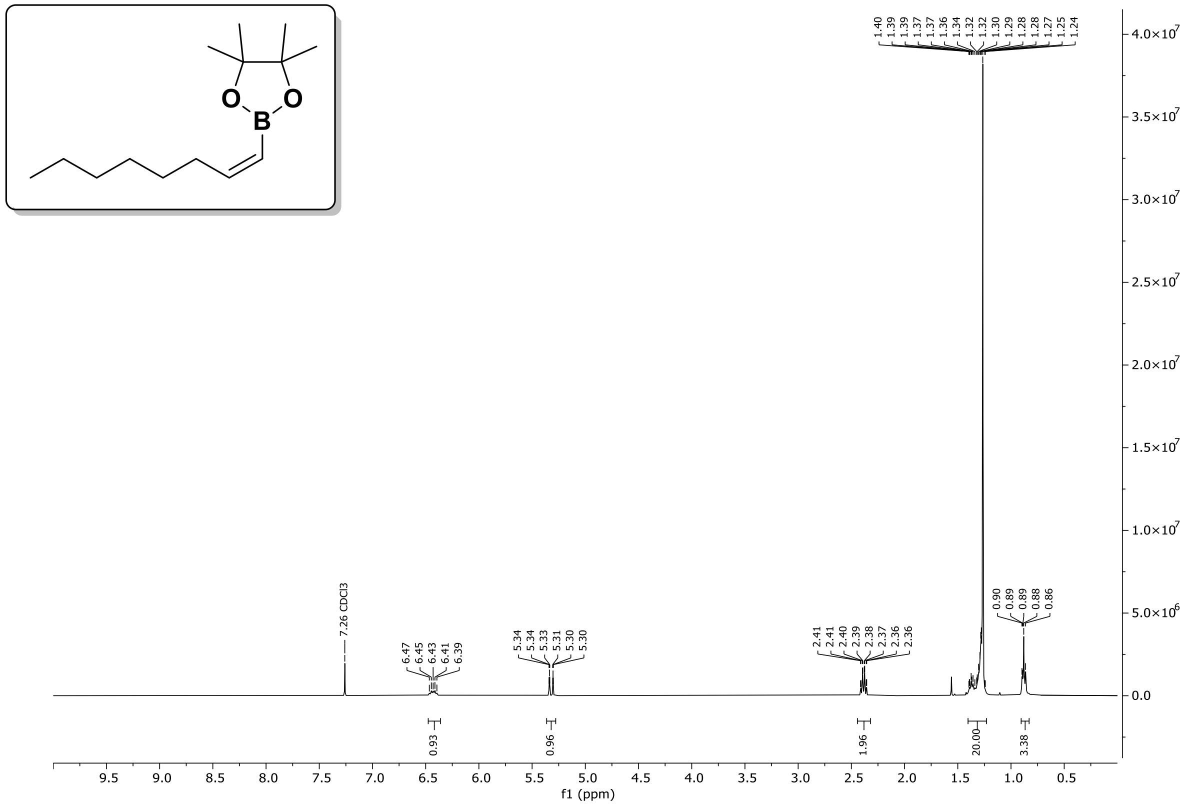

^{1}H NMR of *E*-1b (400 MHz, $CDCl_3$)

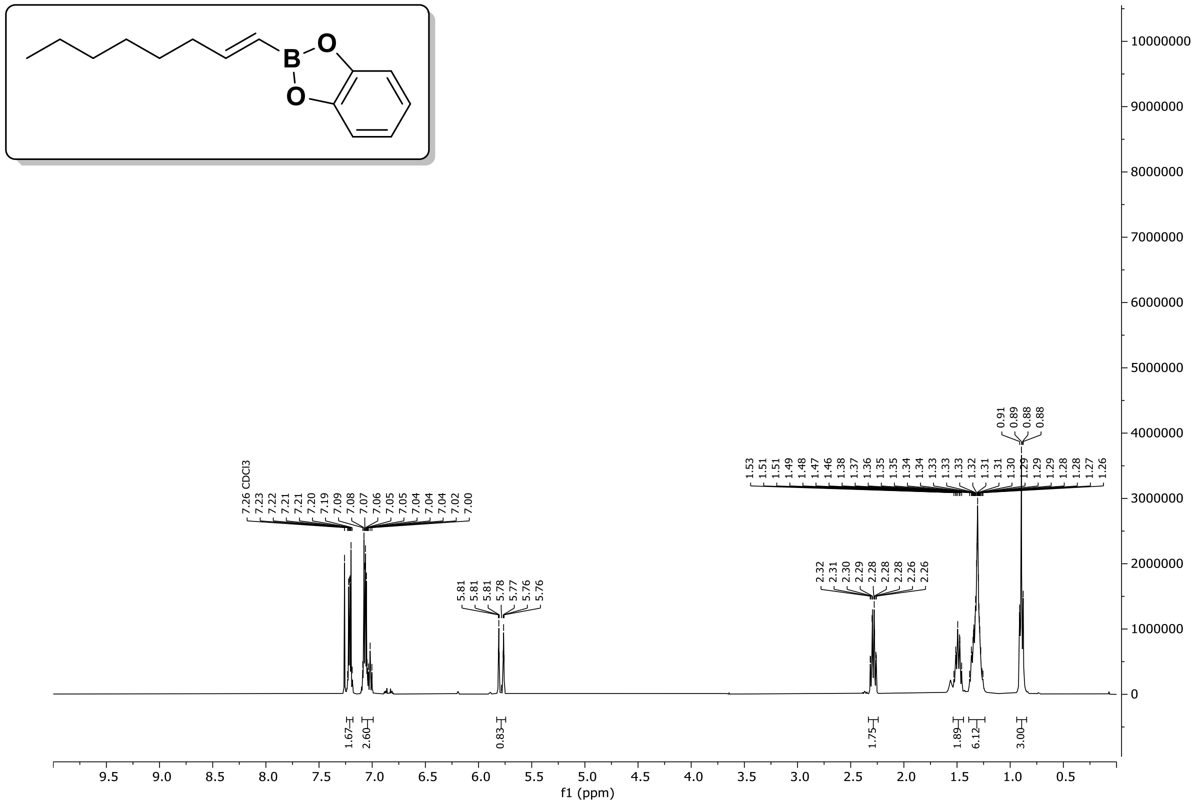

^{1}H NMR of *E*-1c (400 MHz, $CDCl_3$)

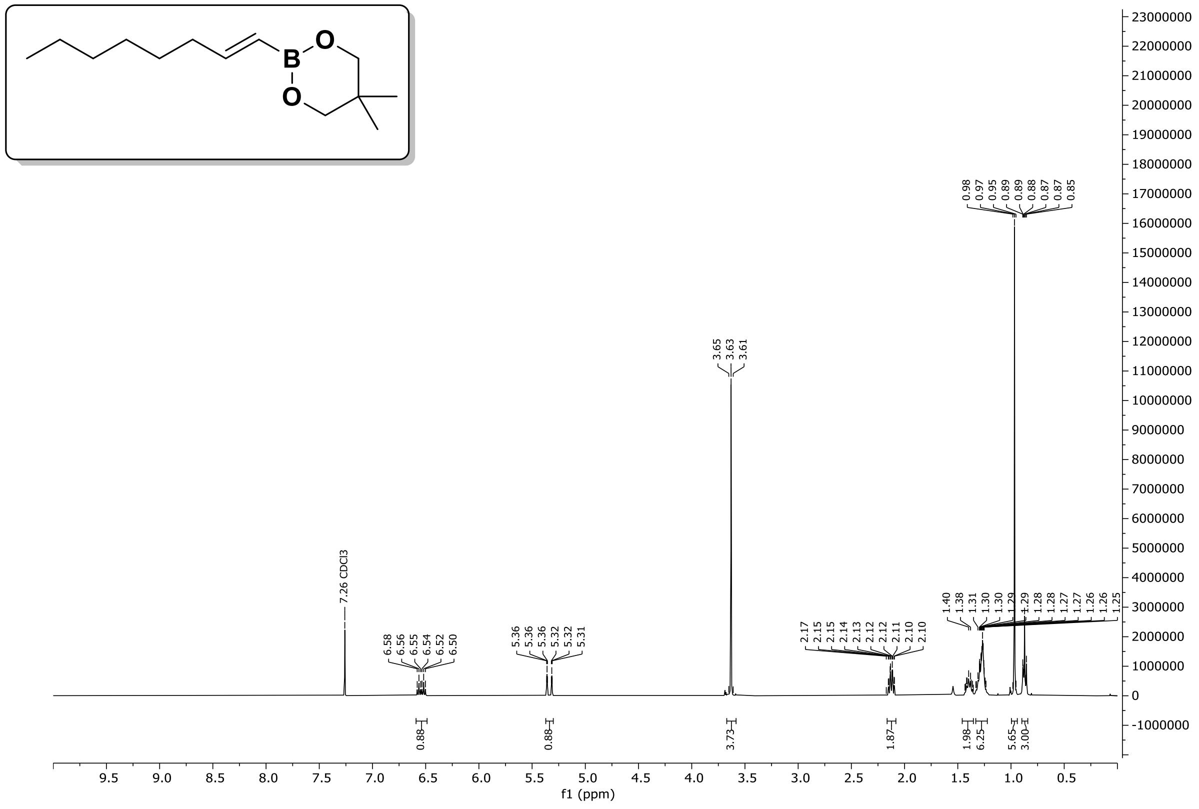

^{1}H NMR of *E*-1d (400 MHz, $CDCl_3$)

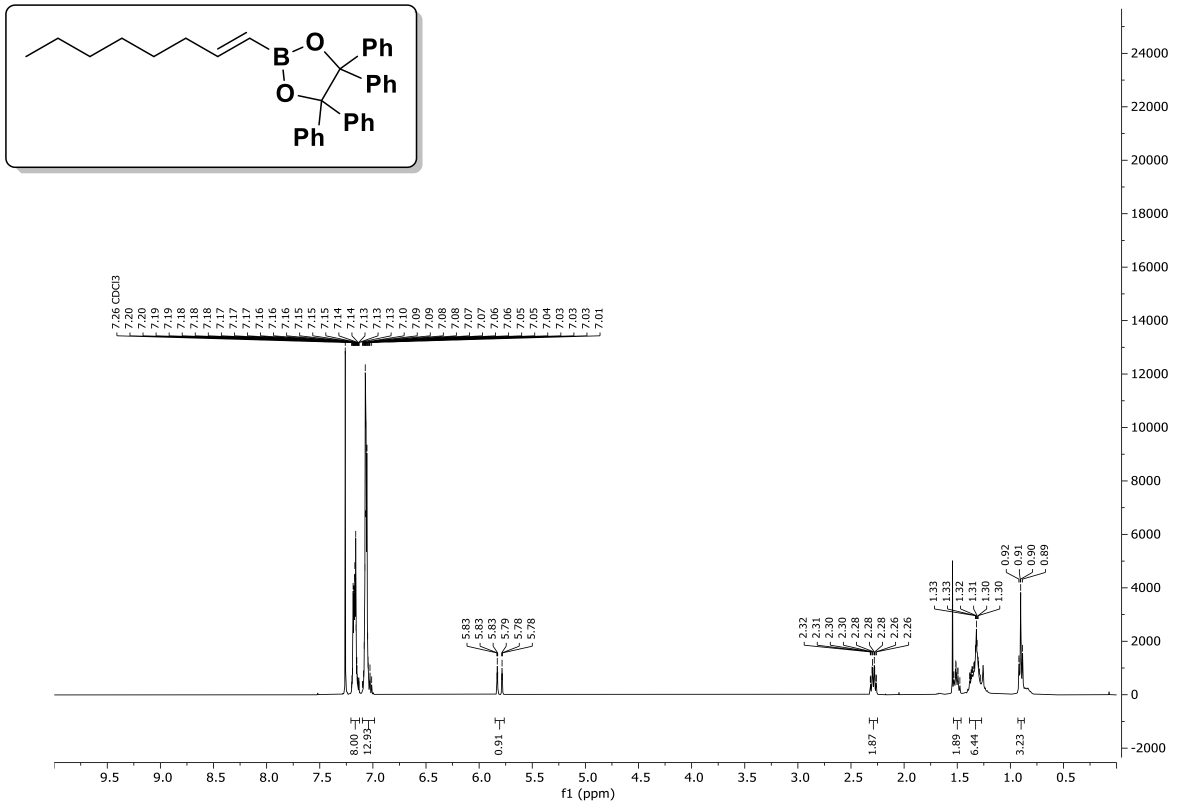

^{13}C NMR of *E*-1d (100 MHz, $CDCl_3$)

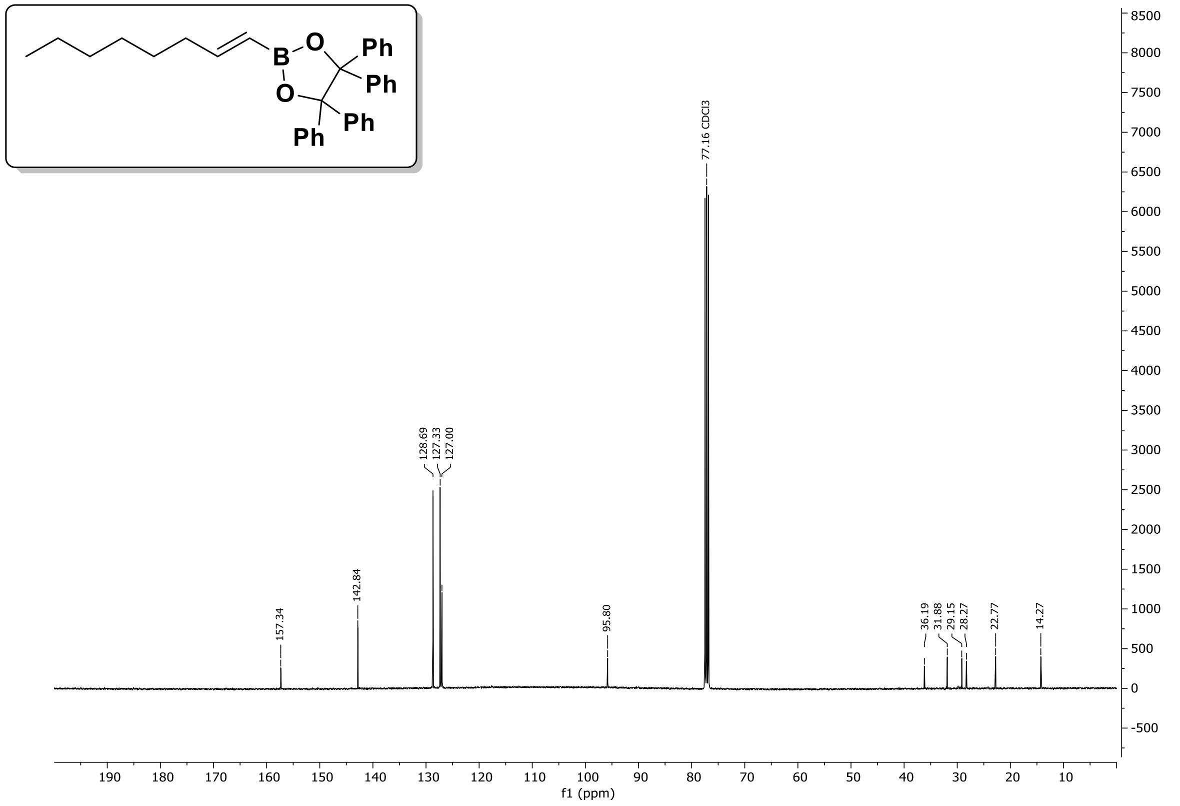

^{1}H NMR of *E*-1e (400 MHz, acetone-*d*$_6$)

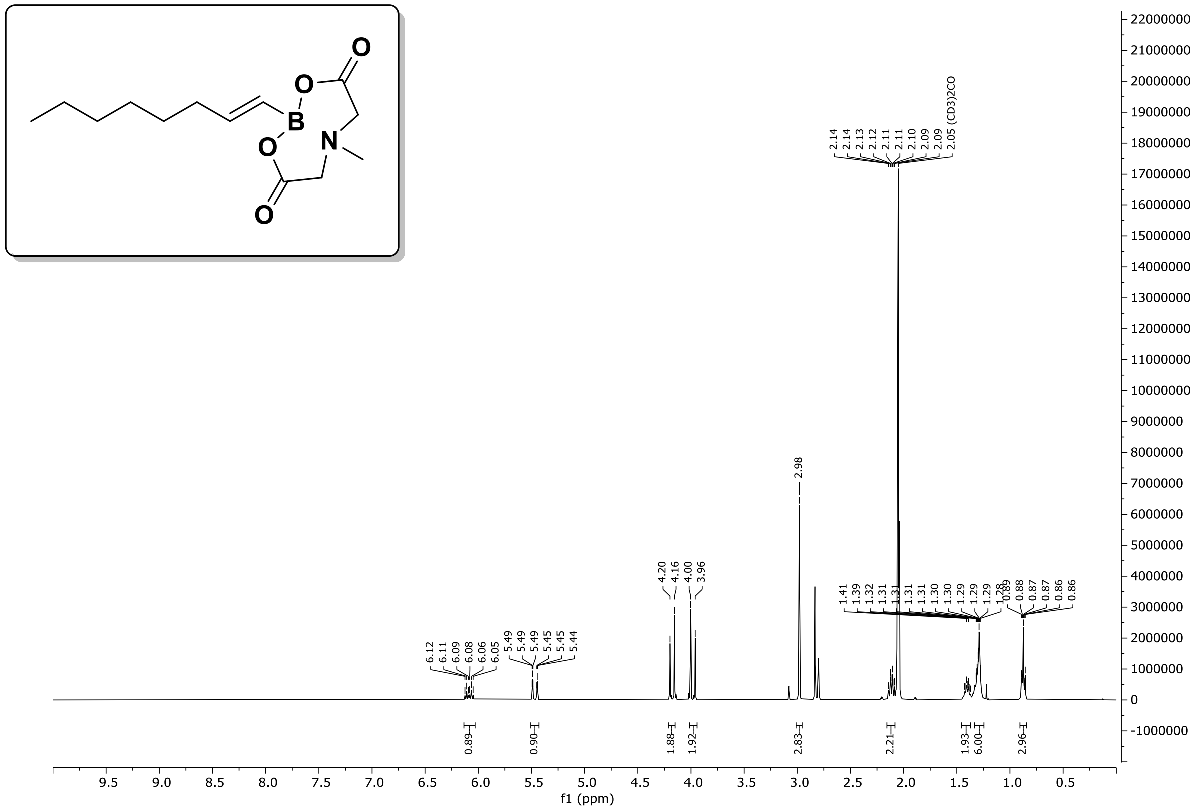

^{1}H NMR of *E*-1f (400 MHz, $CDCl_3$)

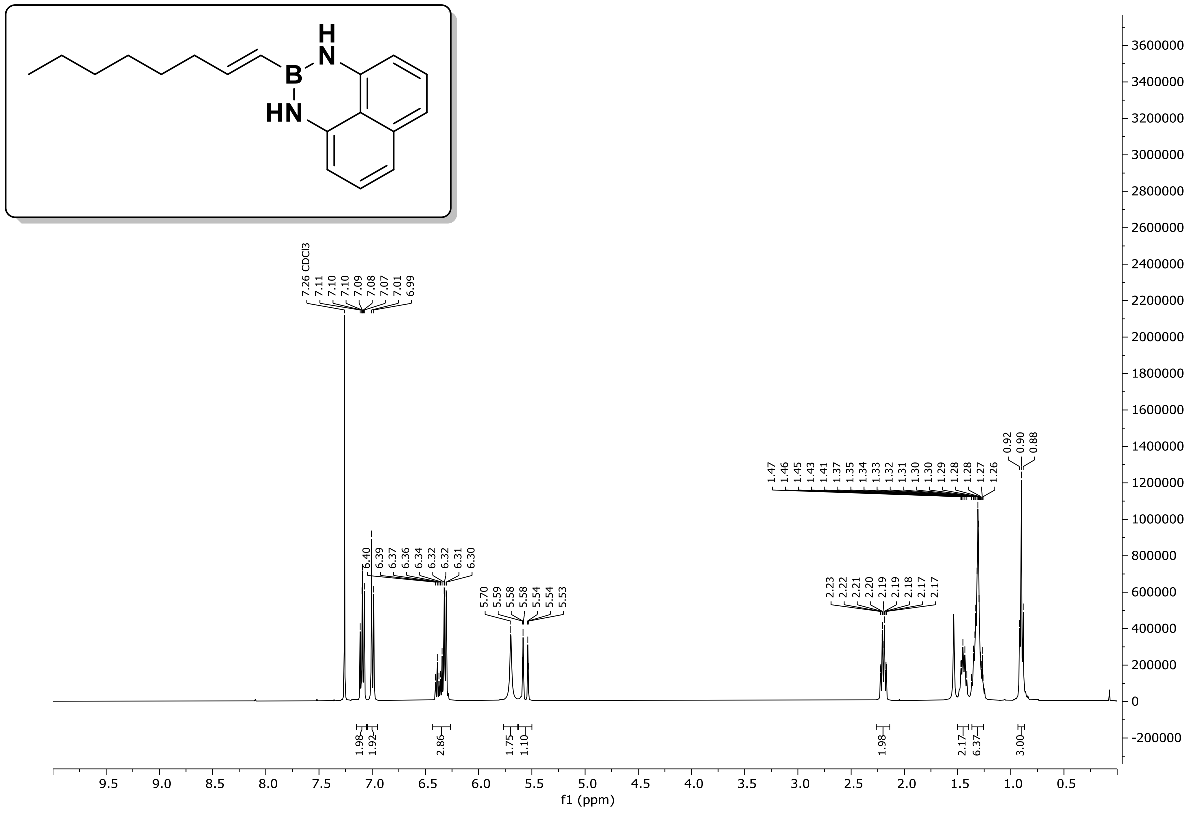

^{1}H NMR of S3 (400 MHz, $CDCl_3$)

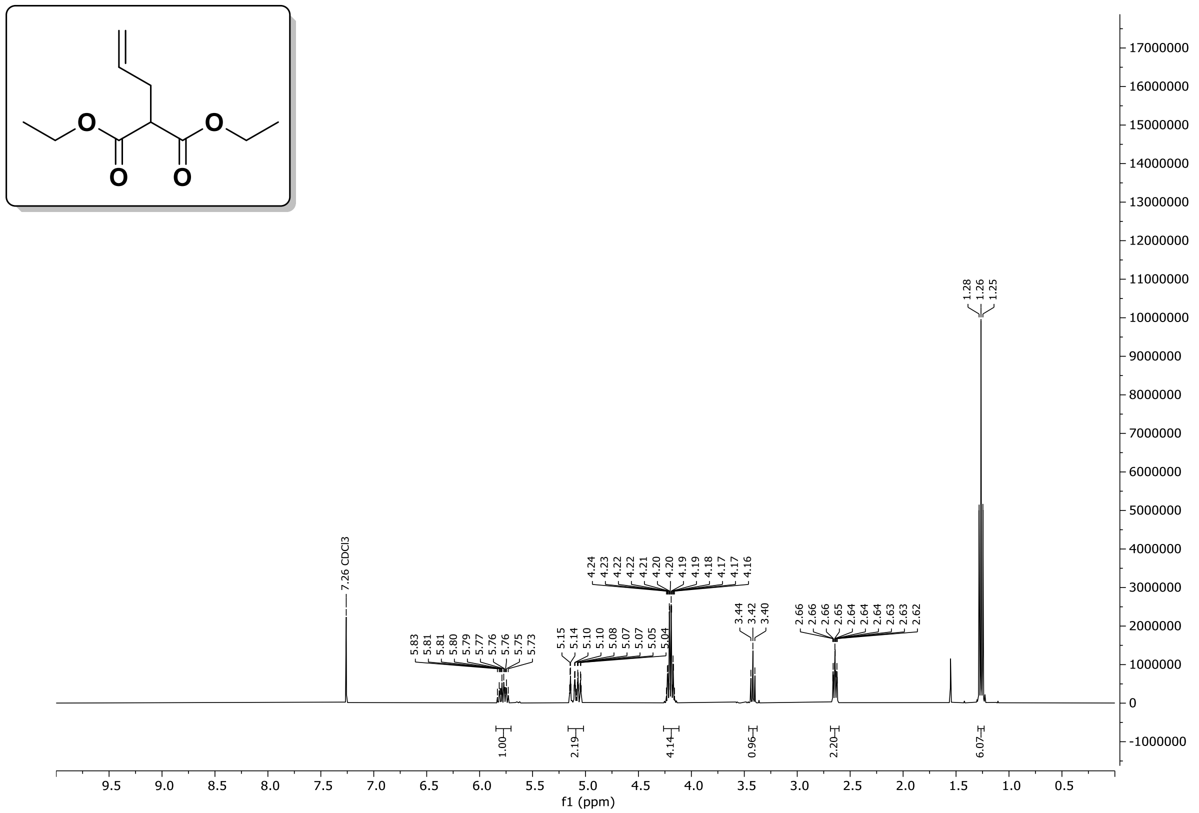

^{1}H NMR of S4 (400 MHz, $CDCl_3$)

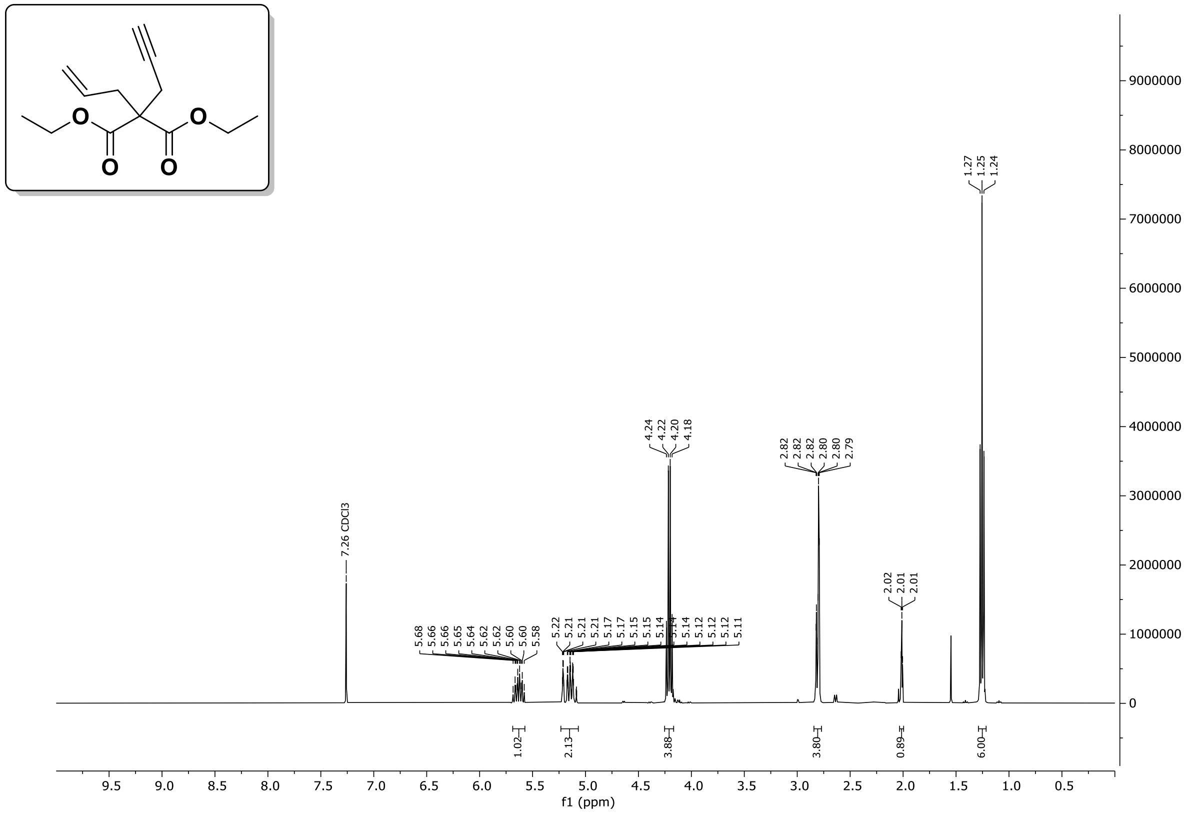

^{1}H NMR of 3a (400 MHz, $CDCl_3$)

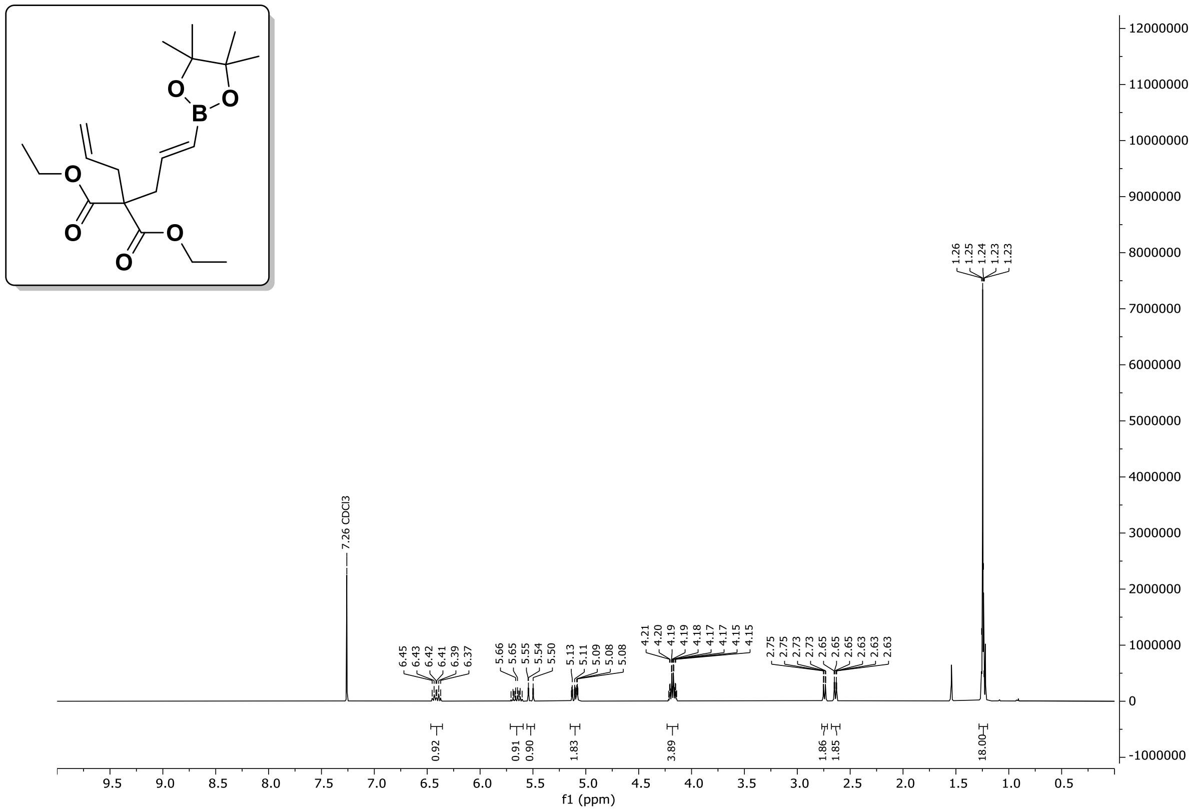

^{13}C NMR of 3a (100 MHz, $CDCl_3$)

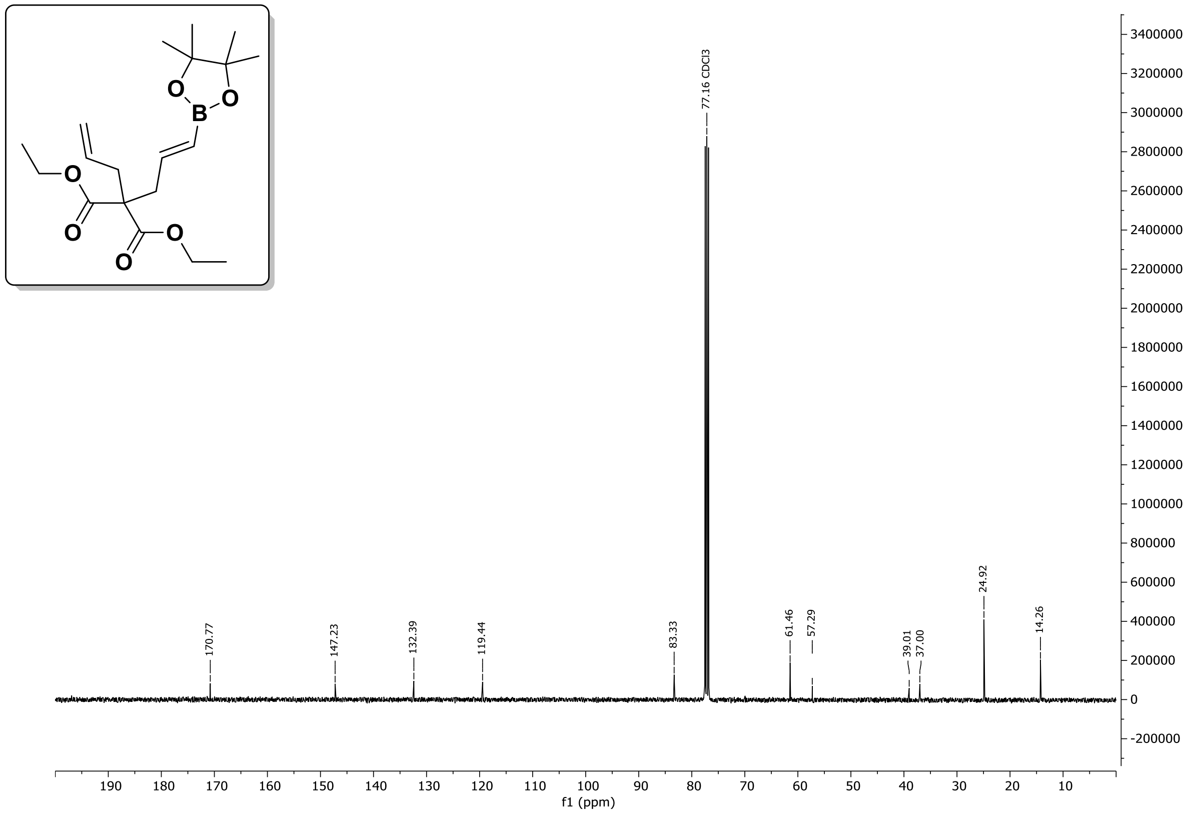

^{1}H NMR of S5 (400 MHz, $CDCl_3$)

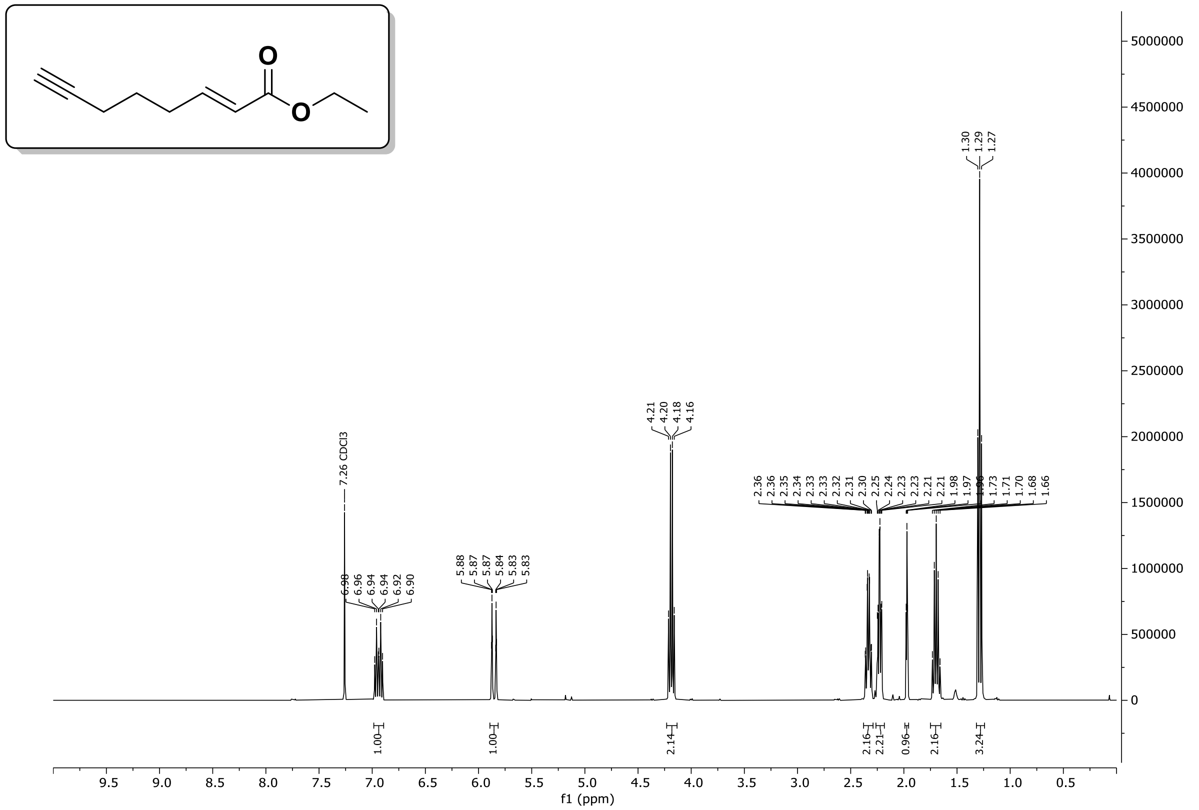

^{1}H NMR of 3b (400 MHz, $CDCl_3$)

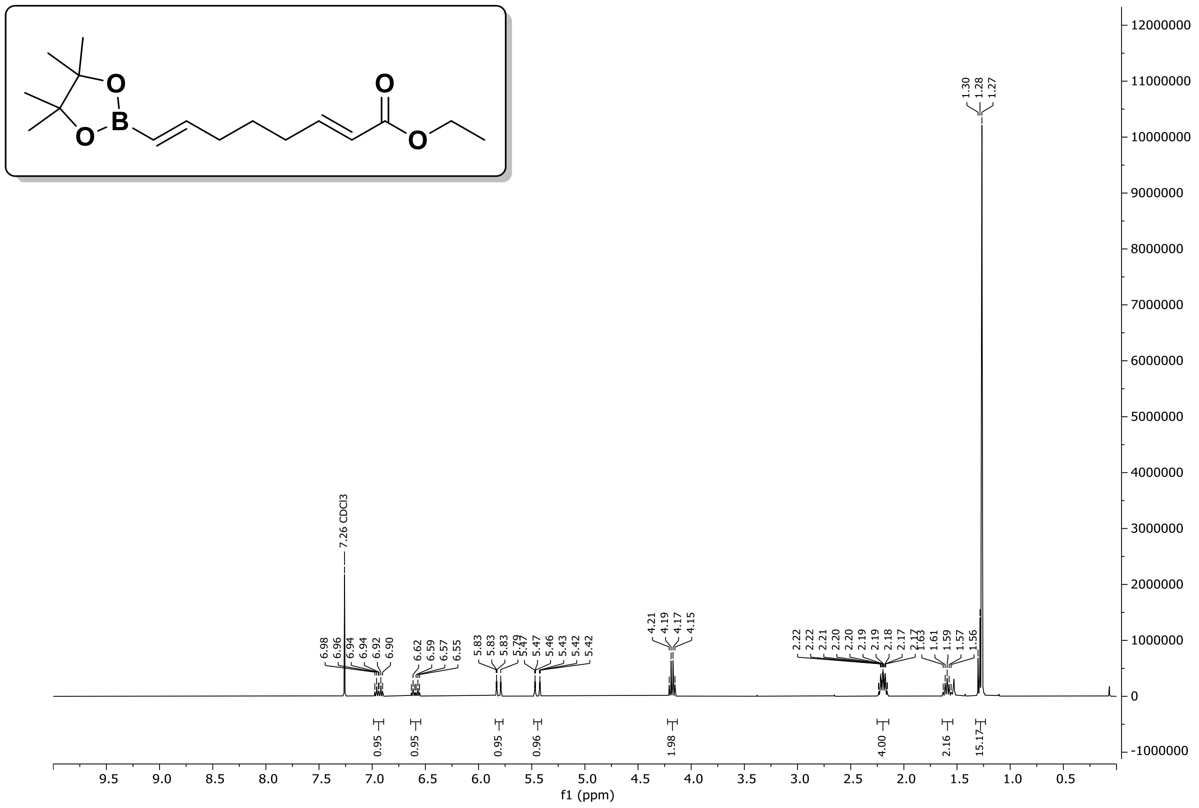

^{1}H NMR of S8 (400 MHz, $CDCl_3$)

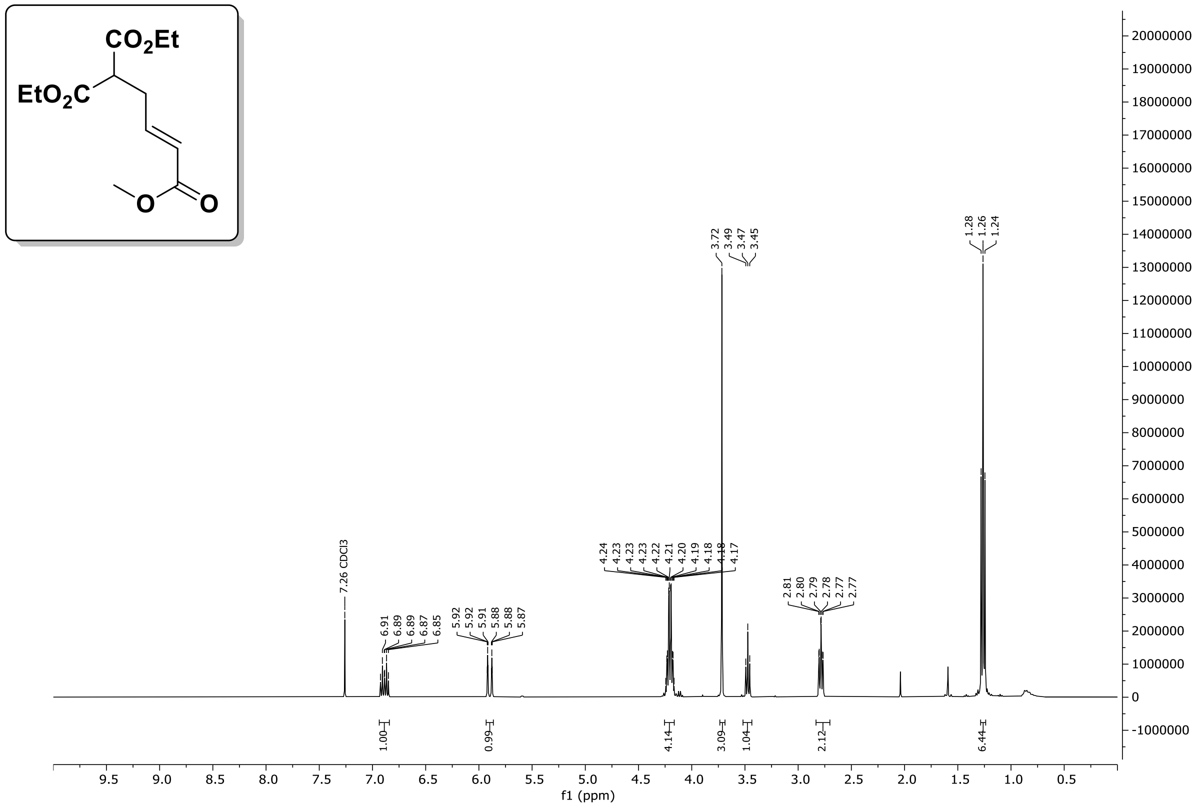

^{1}H NMR of S9 (400 MHz, $CDCl_3$)

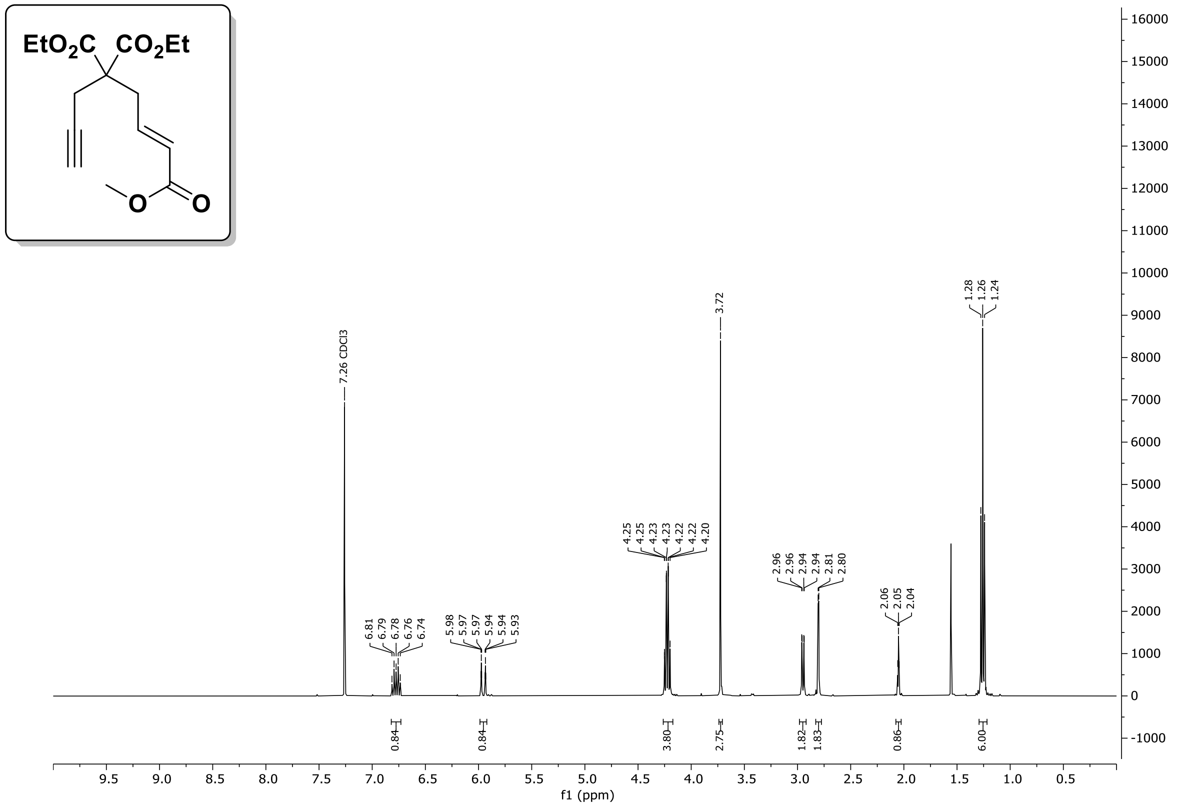

^{1}H NMR of 3c (400 MHz, $CDCl_3$)

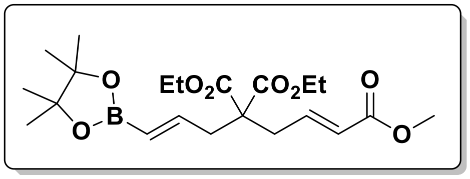

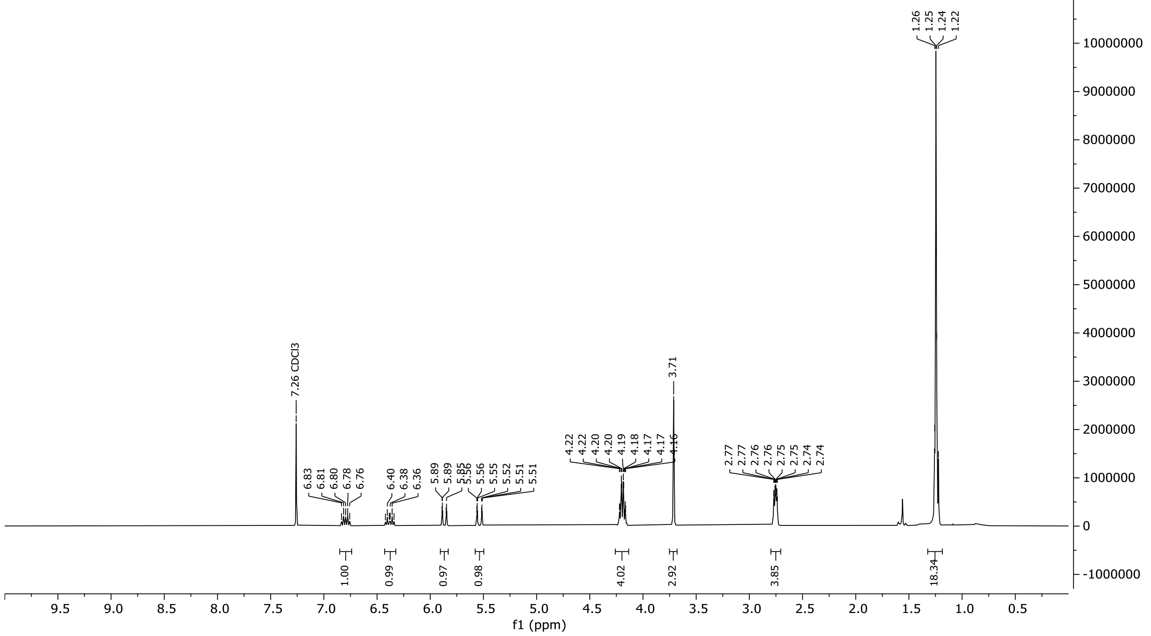

^{13}C NMR of 3c (100 MHz, $CDCl_3$)

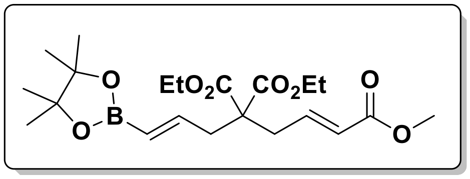

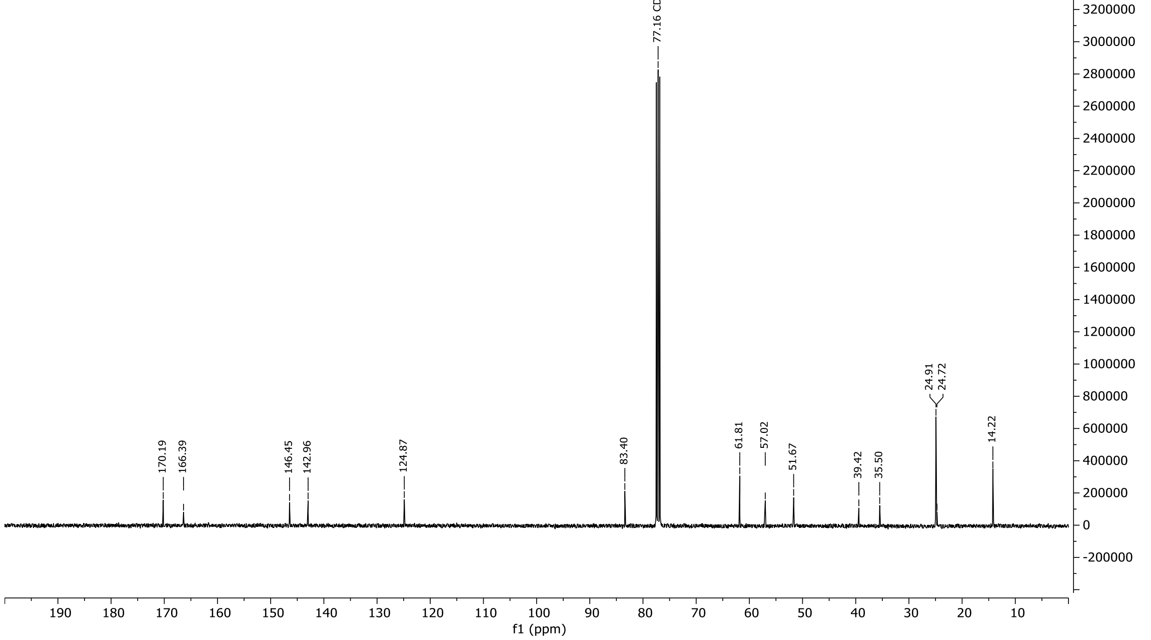

^{1}H NMR of 3h (400 MHz, $CDCl_3$)

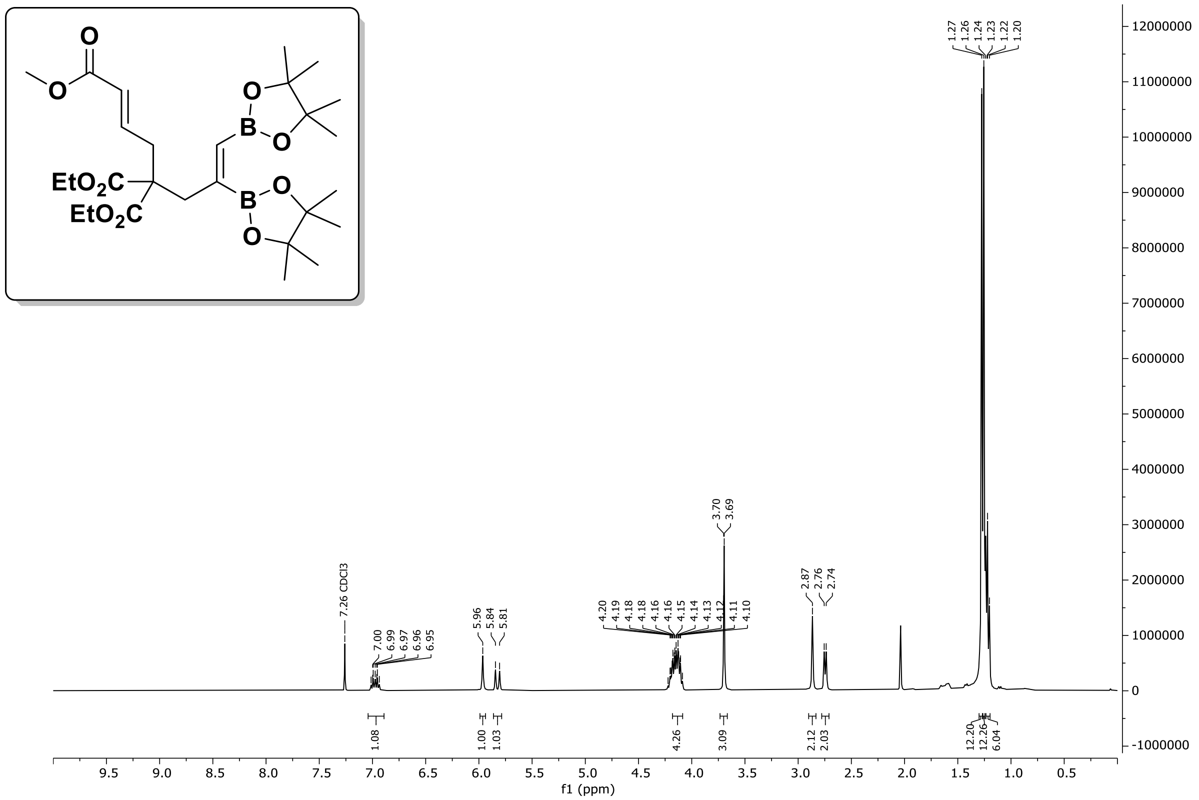

^{13}C NMR of 3h (100 MHz, $CDCl_3$)

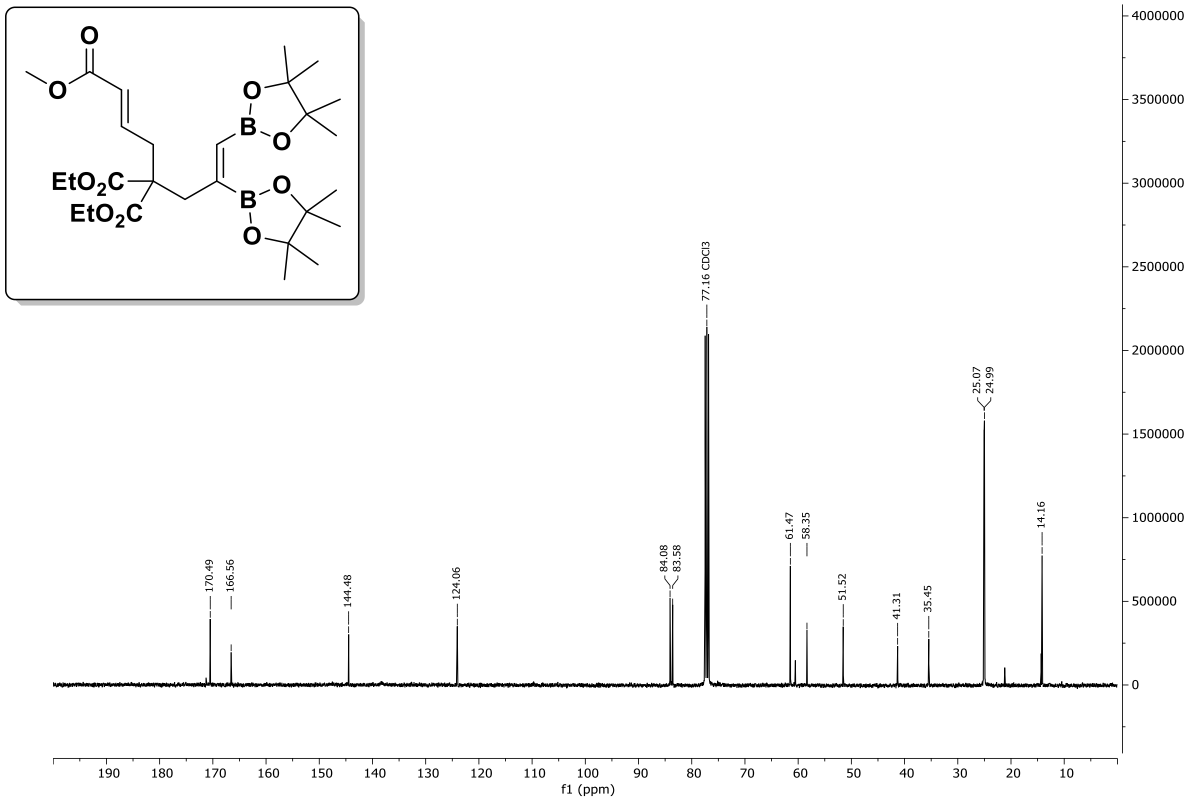

^{1}H NMR of S10 (400 MHz, $CDCl_3$)

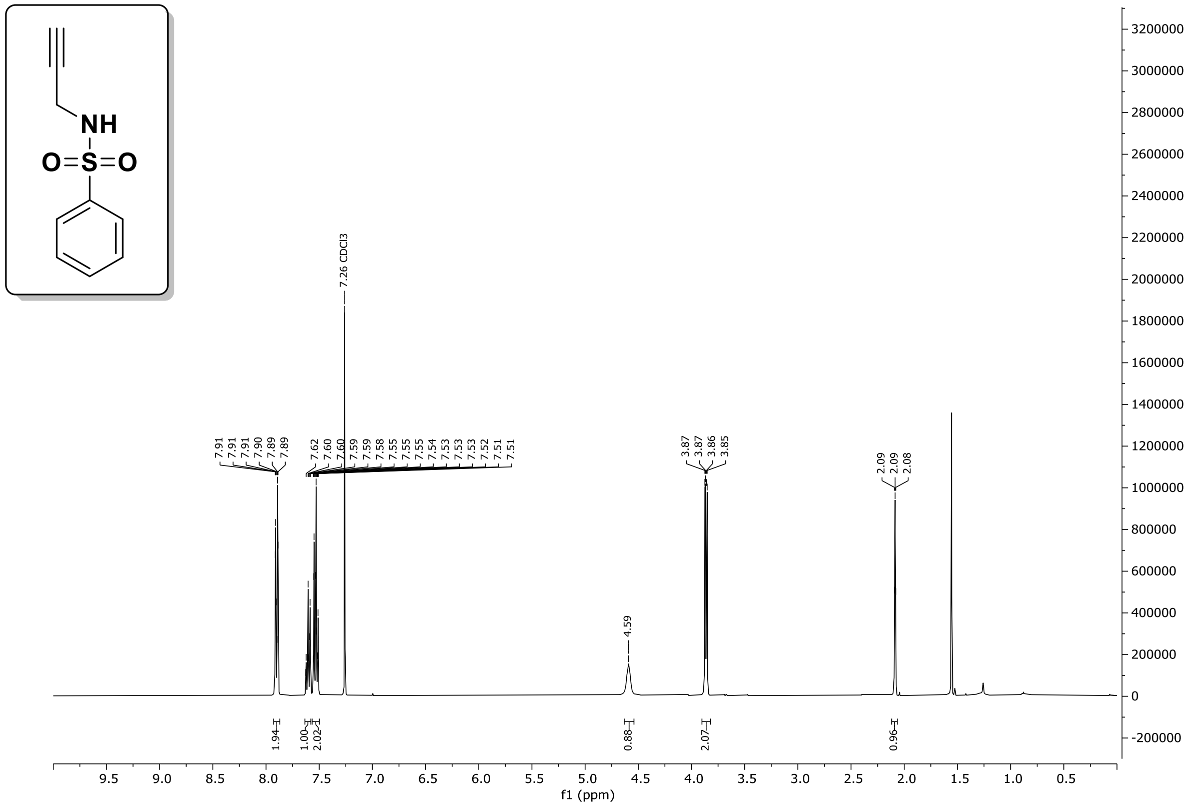

^{1}H NMR of S11 (400 MHz, $CDCl_3$)

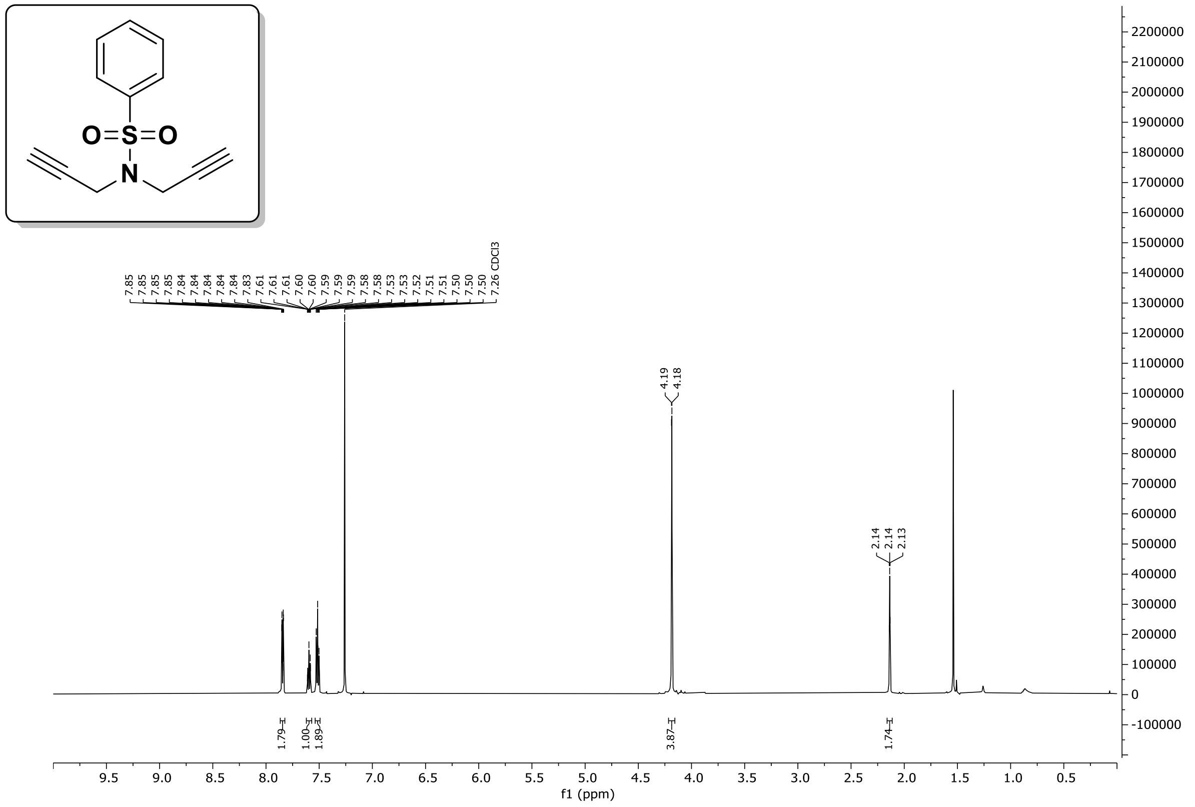

^{13}C NMR of S11 (100 MHz, $CDCl_3$)

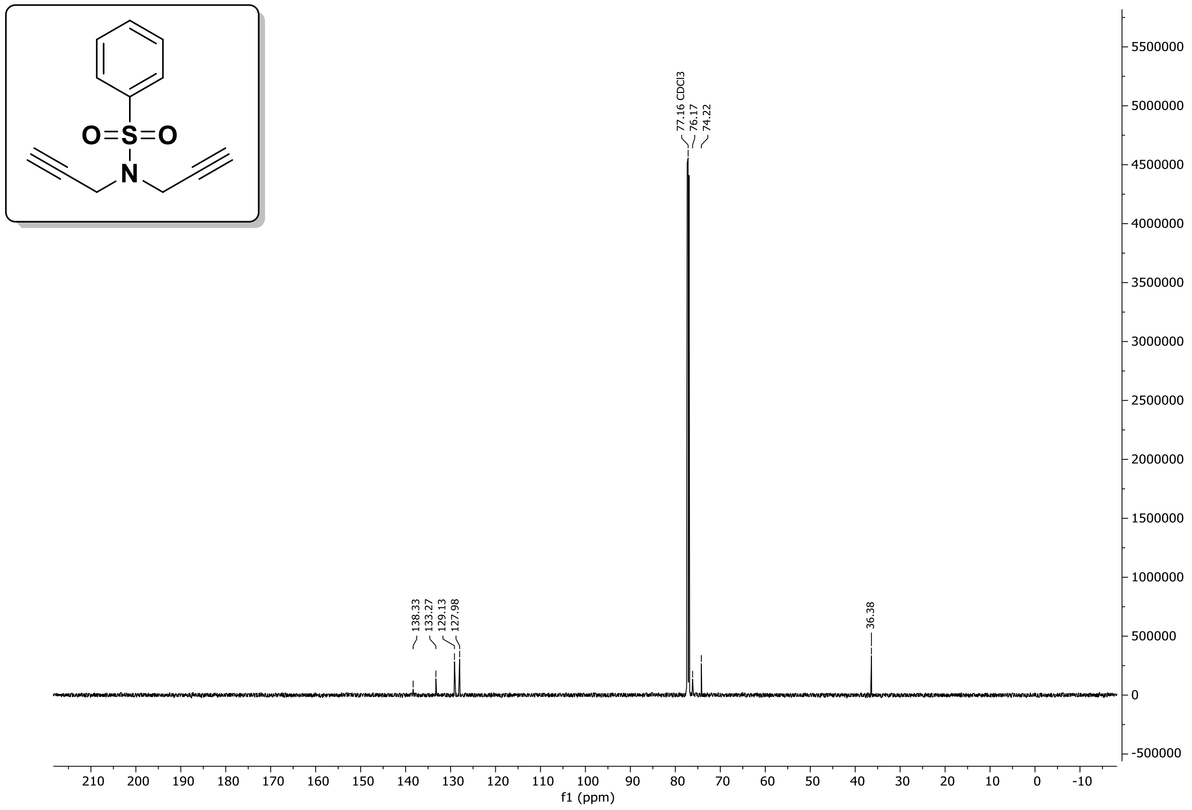

^{1}H NMR of S12 (400 MHz, $CDCl_3$)

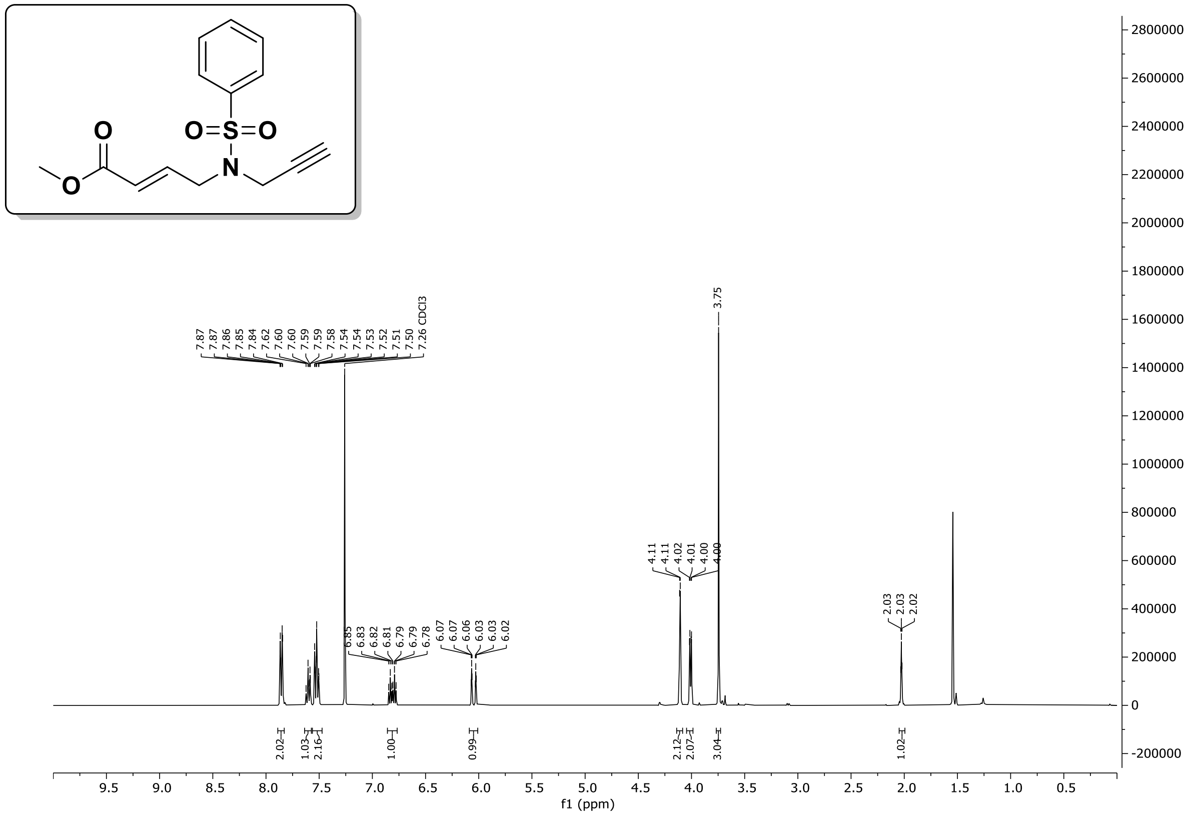

^{13}C NMR of S12 (100 MHz, $CDCl_3$)

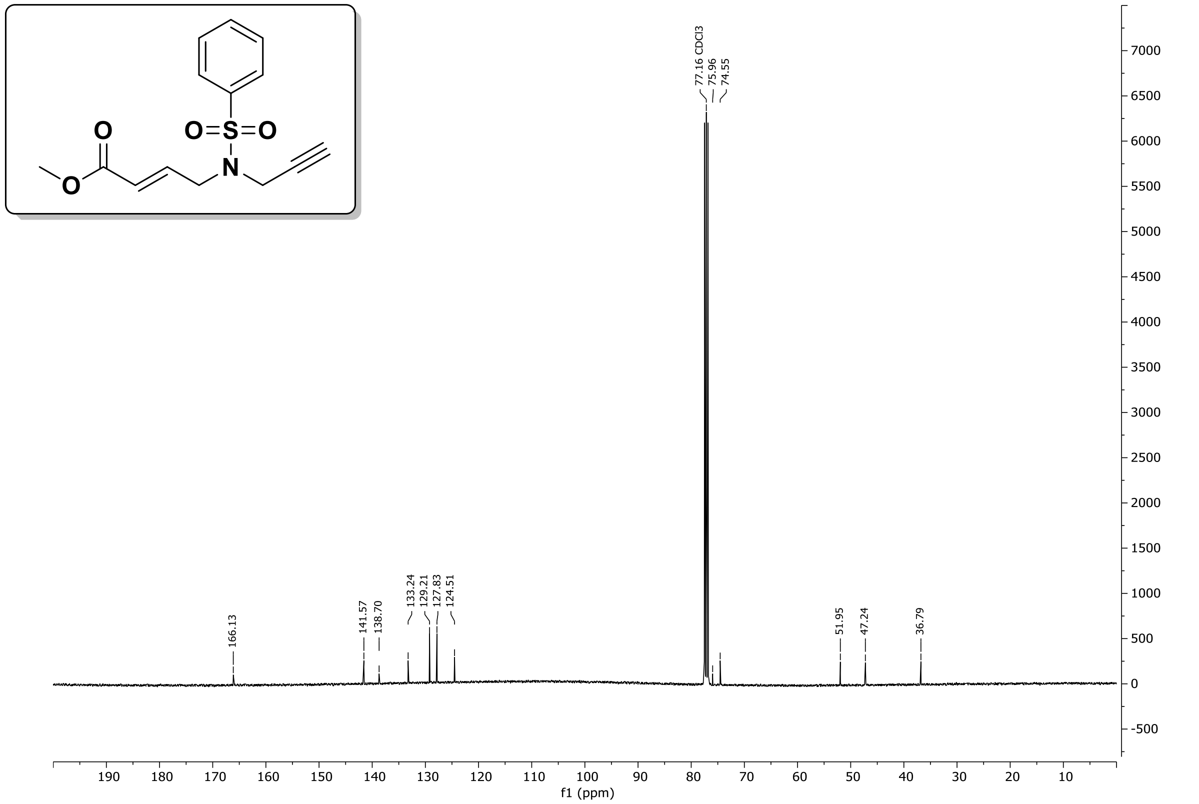

^{1}H NMR of 3d (400 MHz, $CDCl_3$)

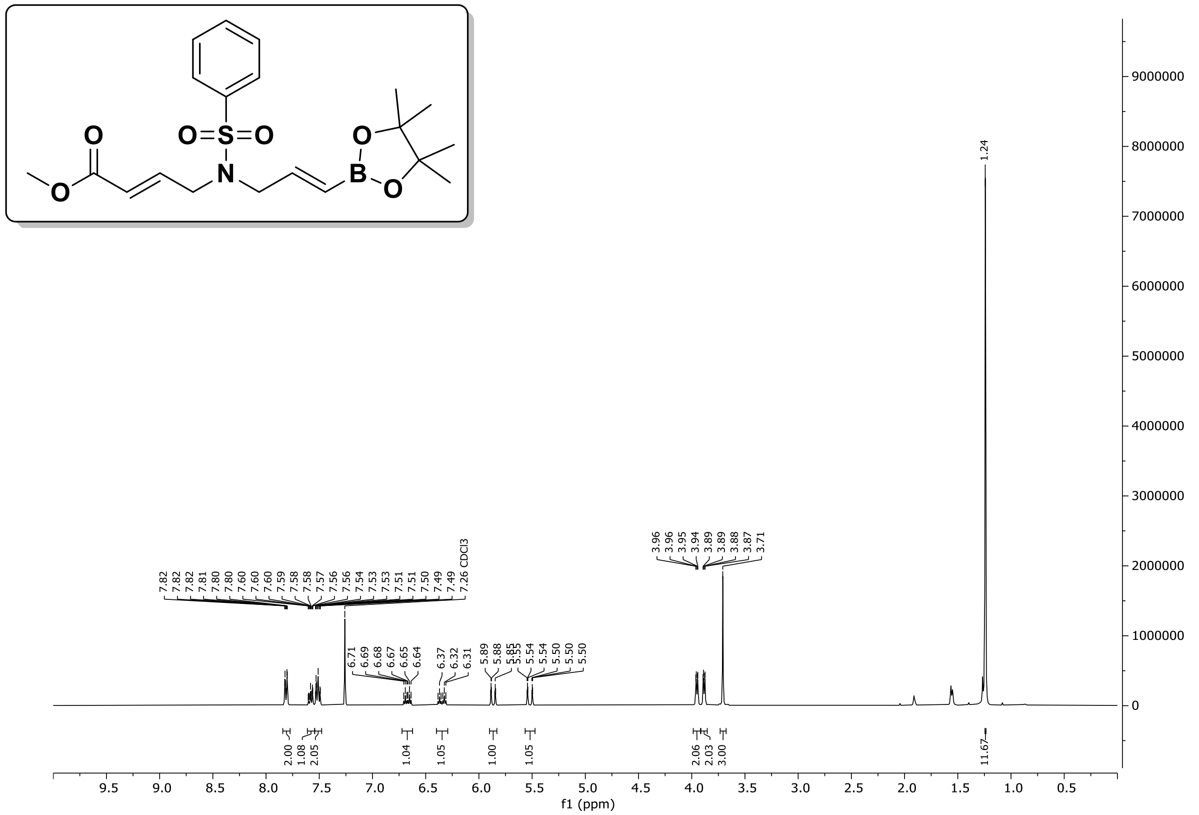

^{13}C NMR of 3d (100 MHz, $CDCl_3$)

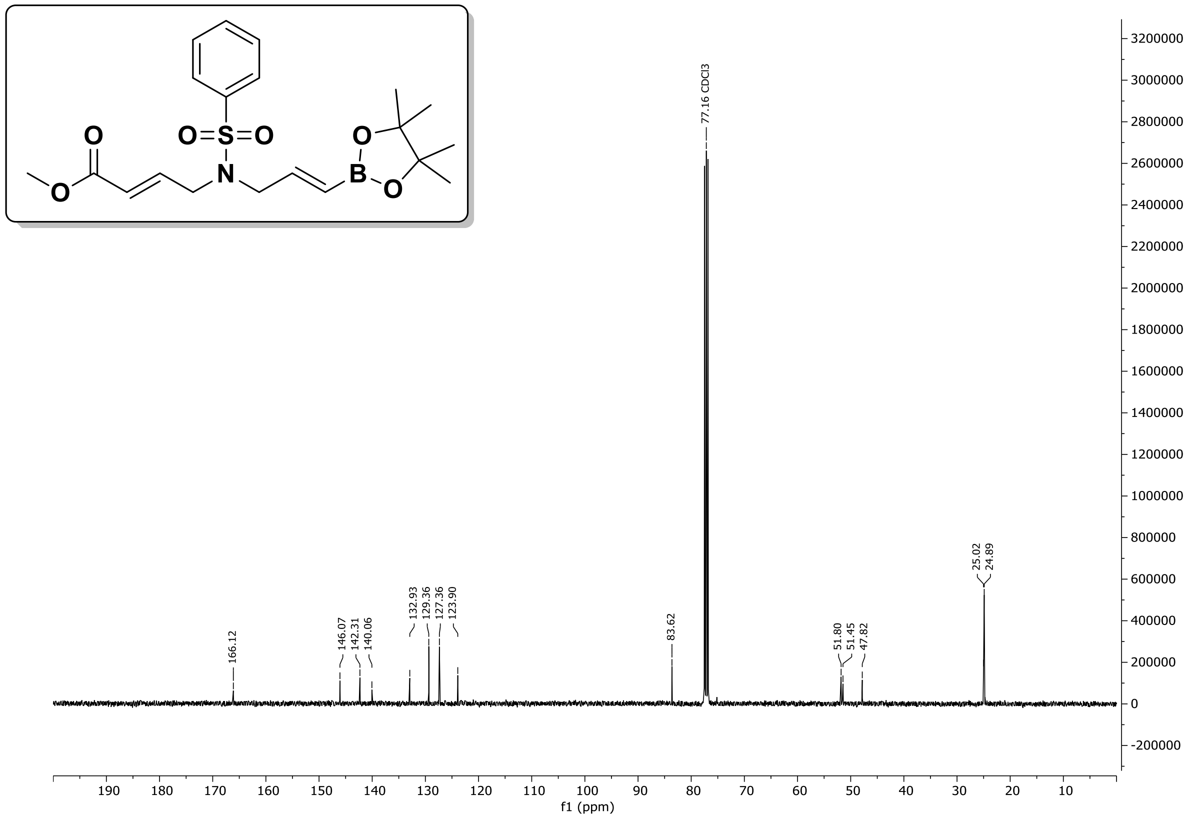

^{1}H NMR of 3e (400 MHz, $CDCl_3$)

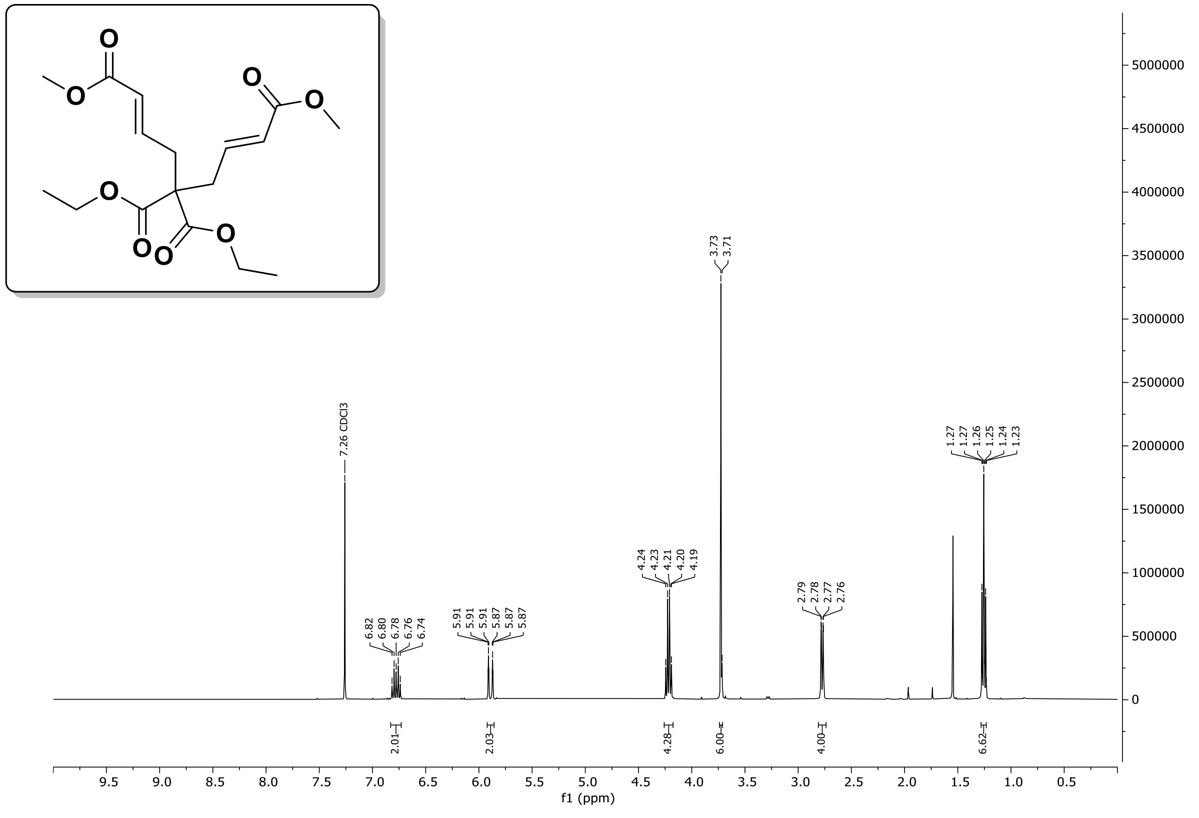

^{1}H NMR of S13 (400 MHz, $CDCl_3$)

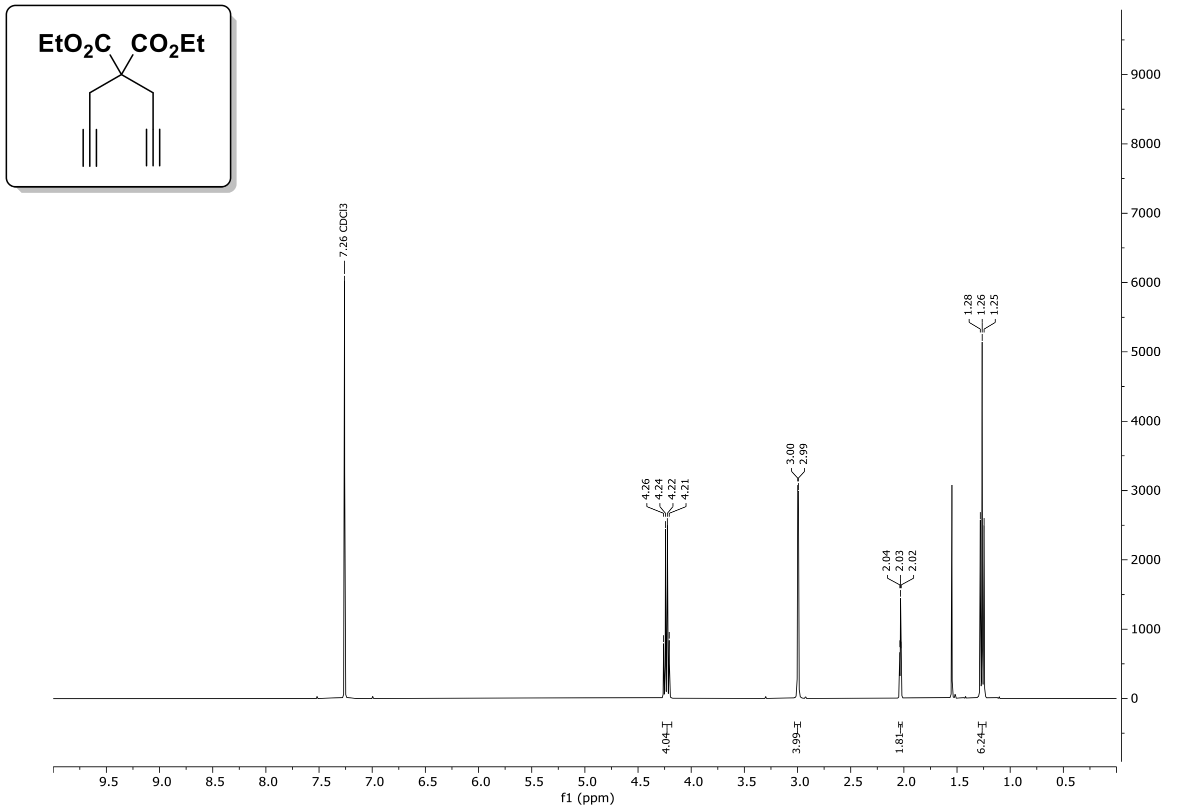

^{1}H NMR of 3f (400 MHz, $CDCl_3$)

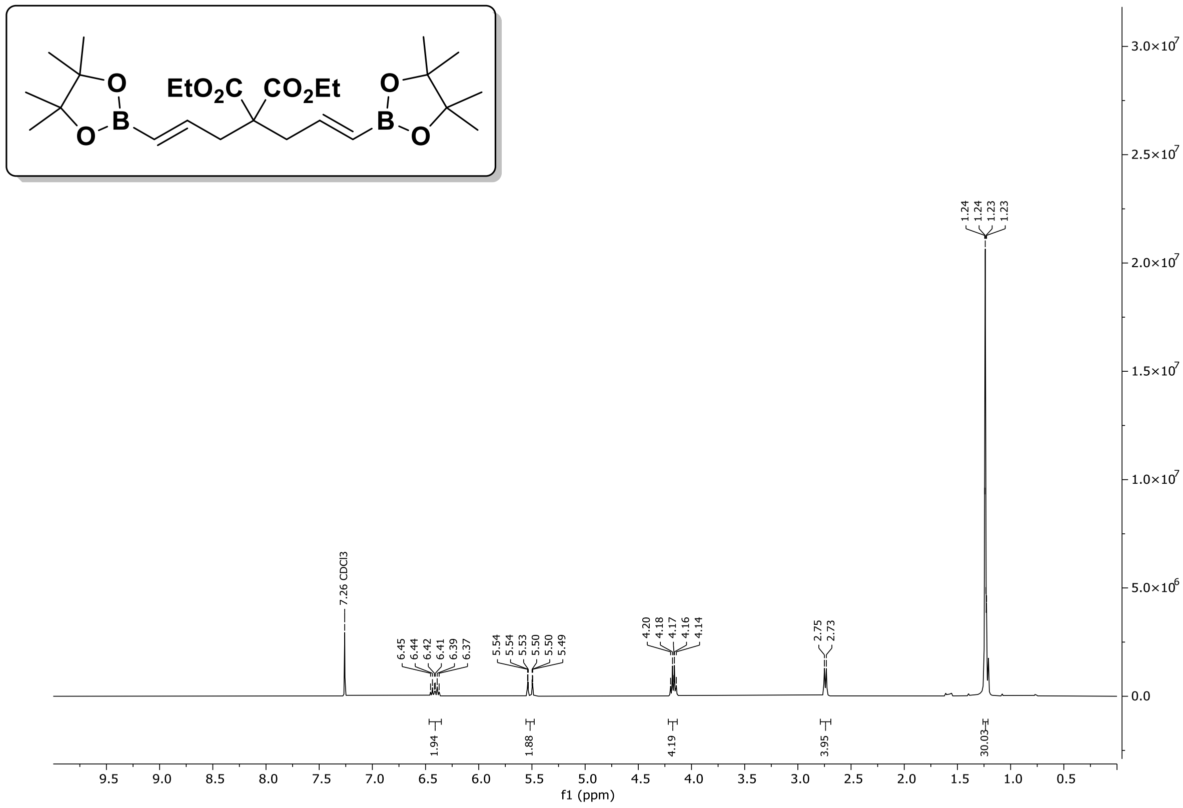

^{13}C NMR of 3f (100 MHz, CDCl$_3$)

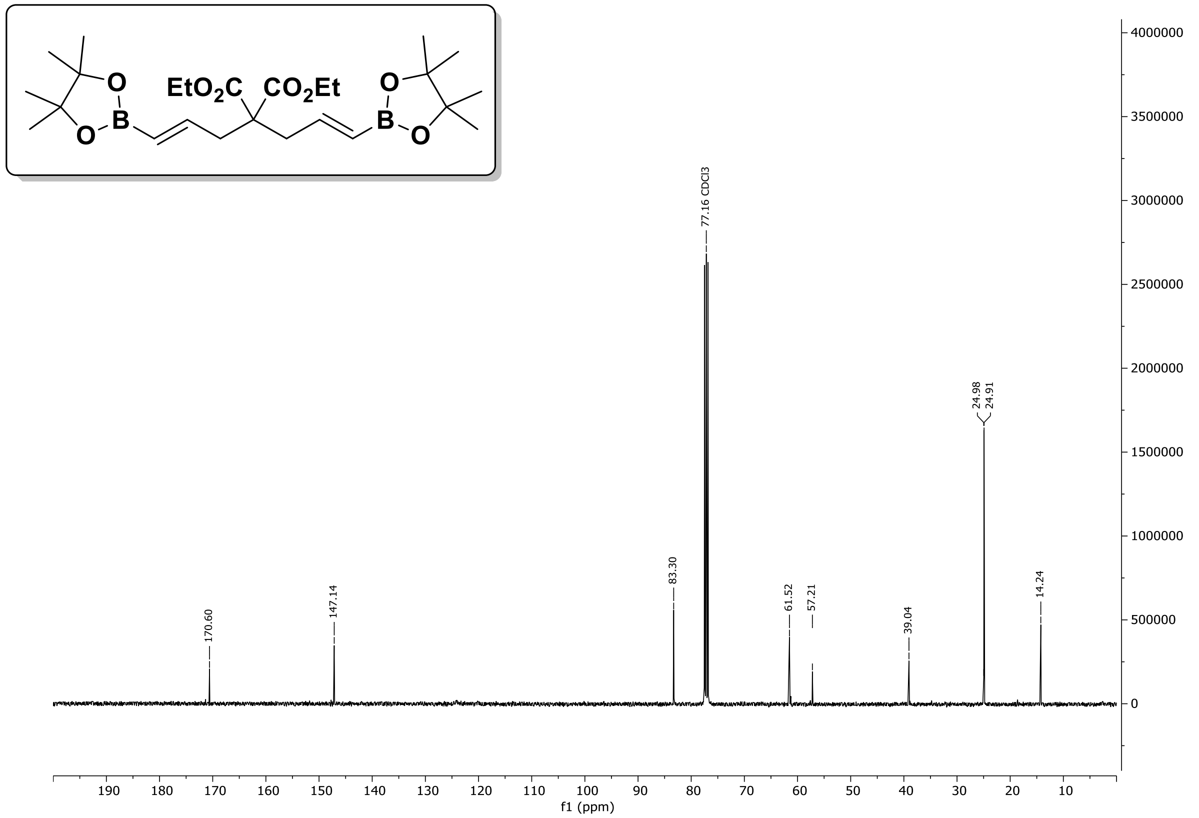

^{1}H NMR of 3g (400 MHz, CDCl$_3$)

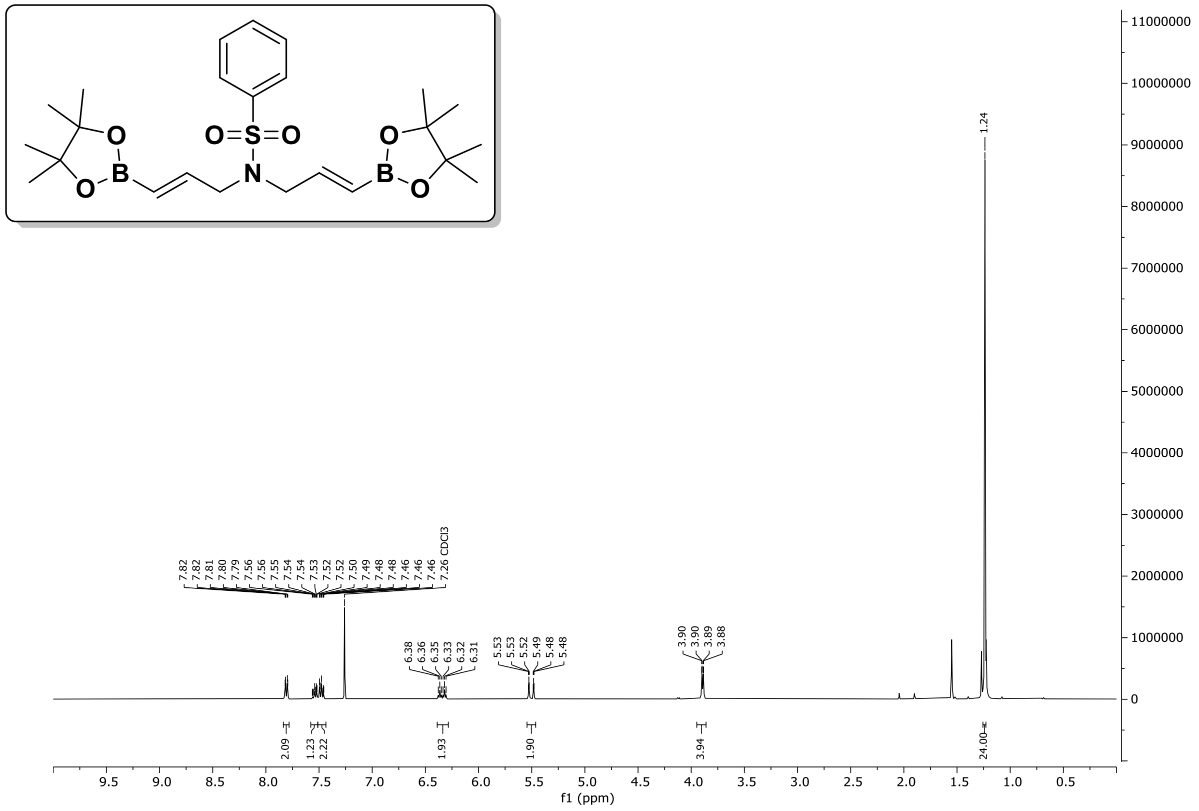

^{13}C NMR of 3g (100 MHz, $CDCl_3$)

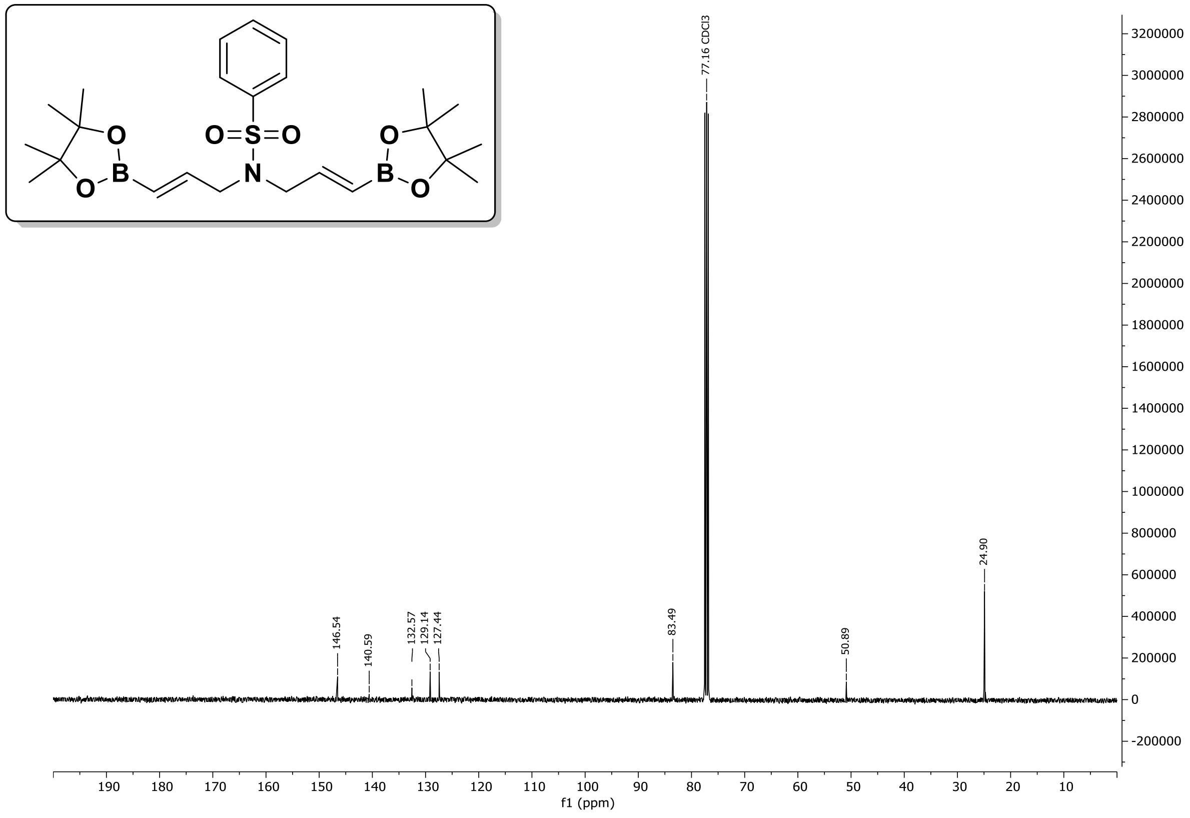

^{1}H NMR of S14 (400 MHz, $CDCl_3$)

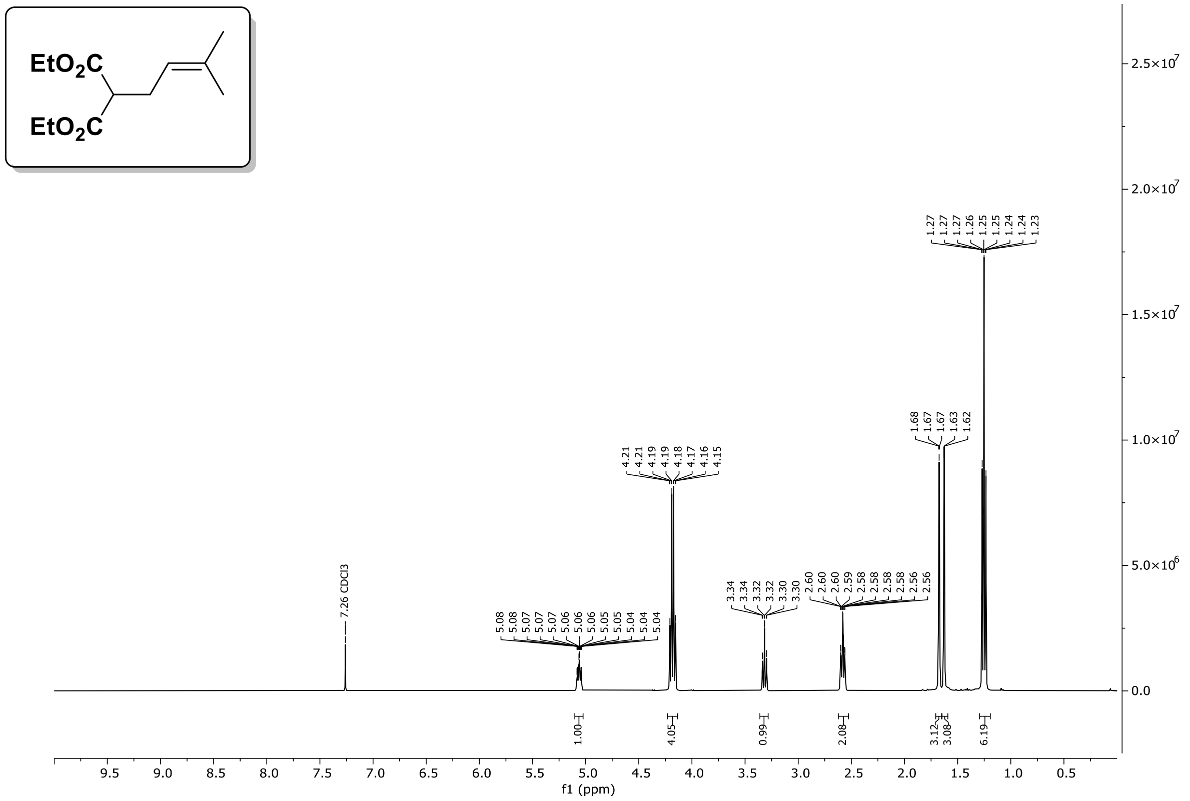

^{1}H NMR of S15 (400 MHz, $CDCl_3$)

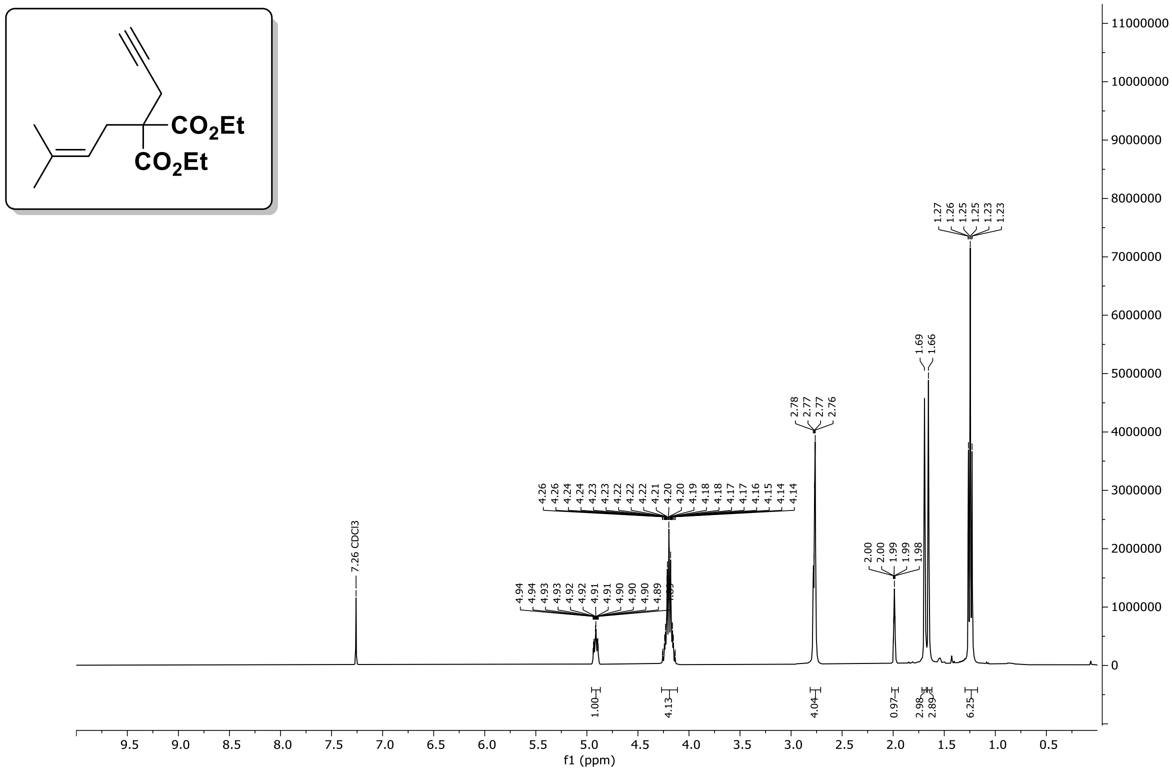

^{1}H NMR of S16 (400 MHz, $CDCl_3$)

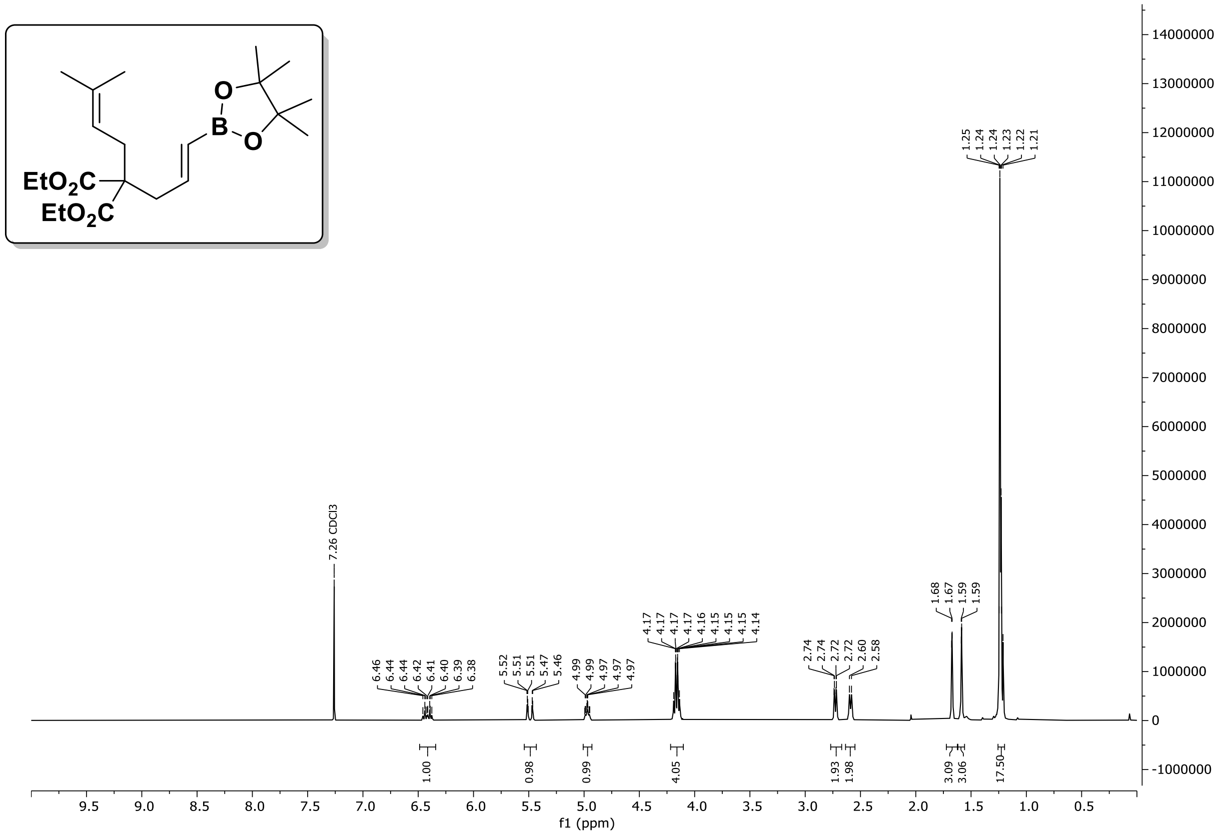

^{13}C NMR of S16 (100 MHz, $CDCl_3$)

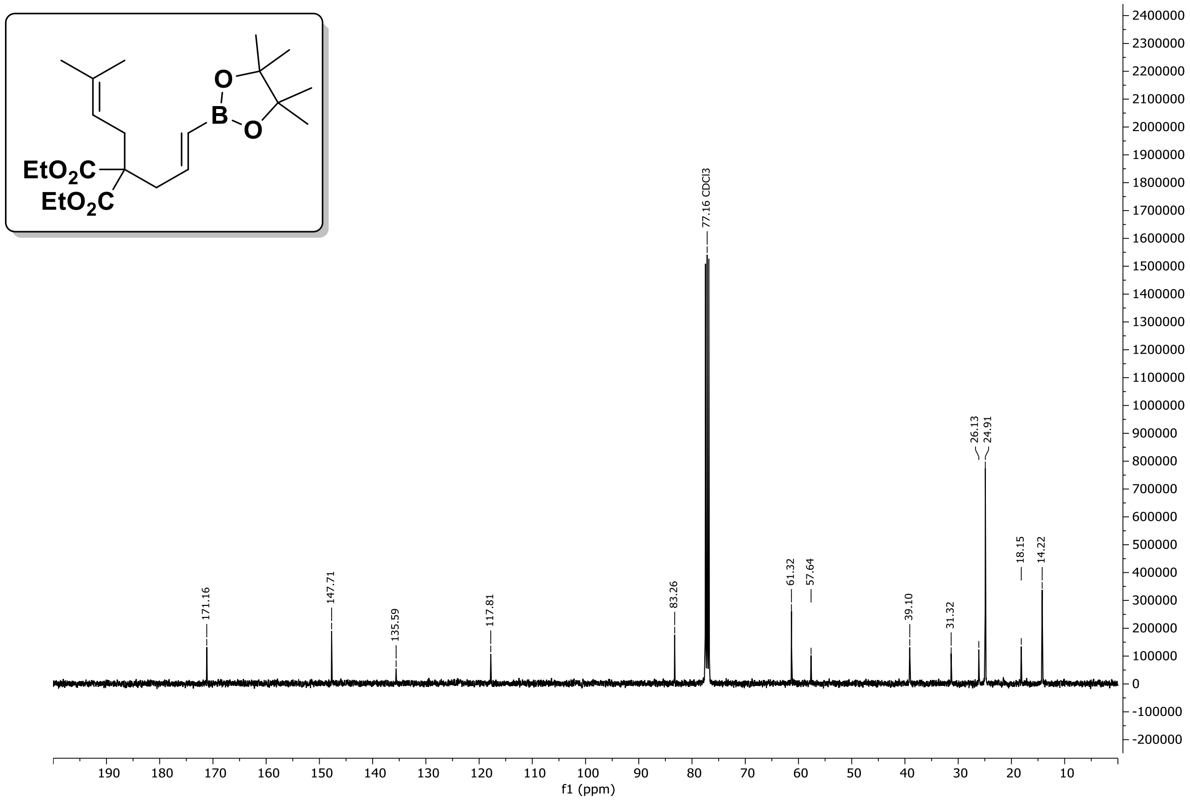

^{1}H NMR of 4b (400 MHz, $CDCl_3$)

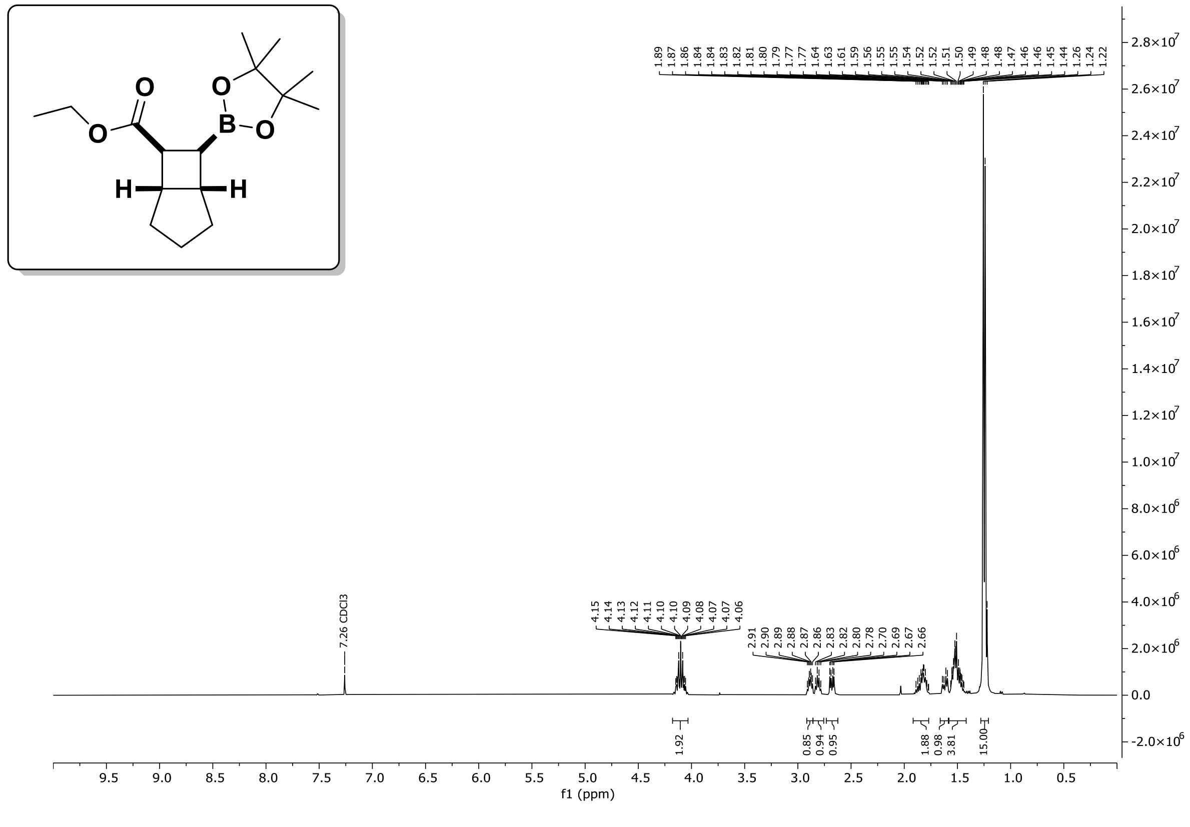

^{13}C NMR of 4b (100 MHz, CDCl$_3$)

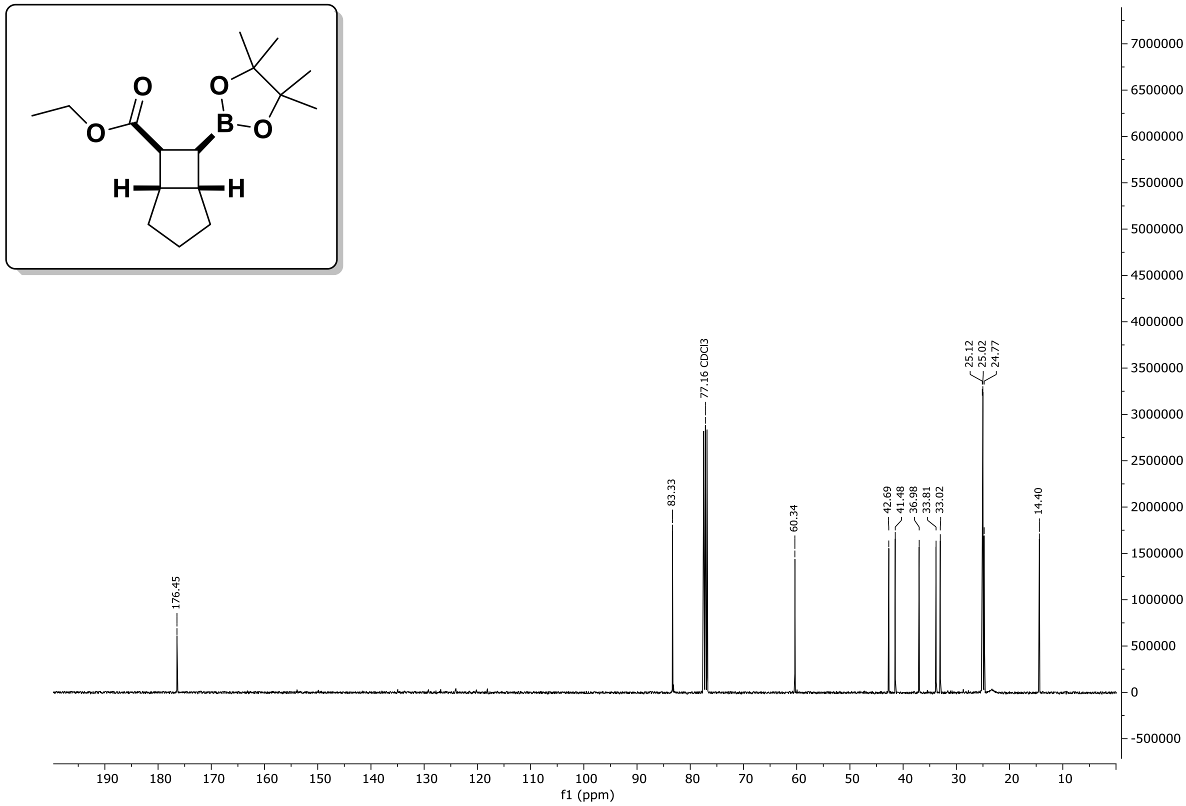

^{1}H NMR of 4c (400 MHz, CDCl$_3$)

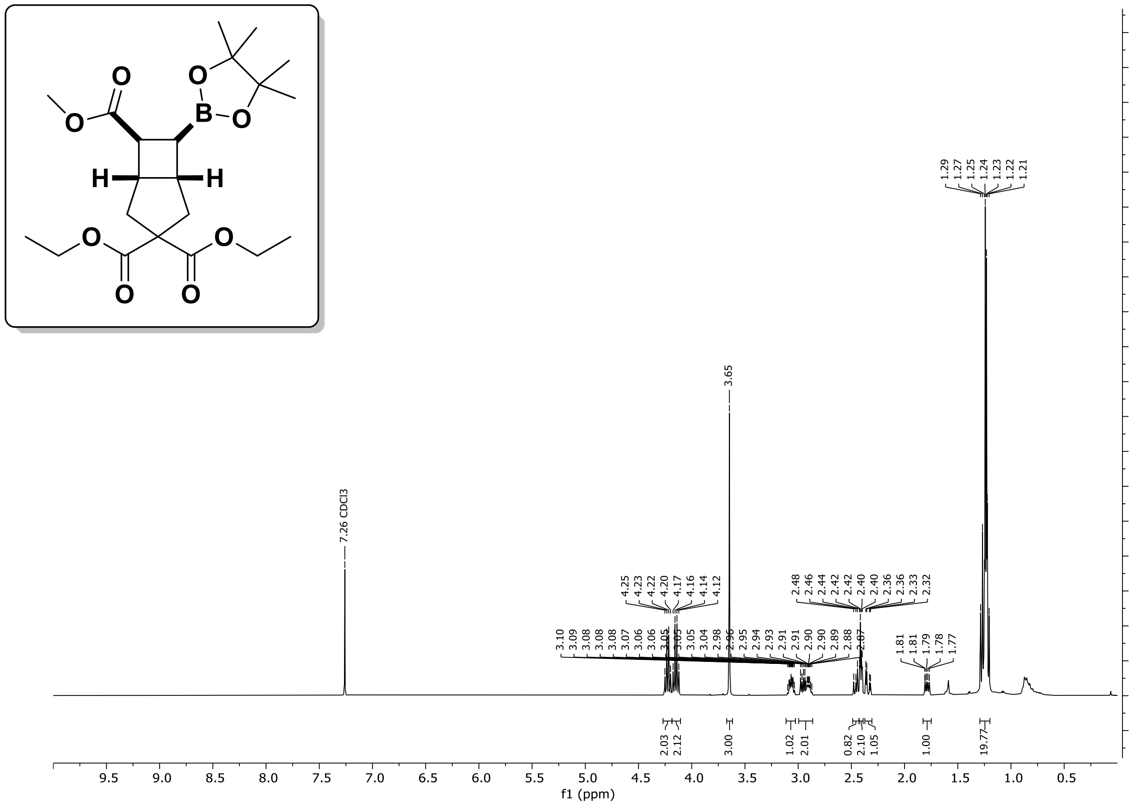

^{13}C NMR of 4c (100 MHz, $CDCl_3$)

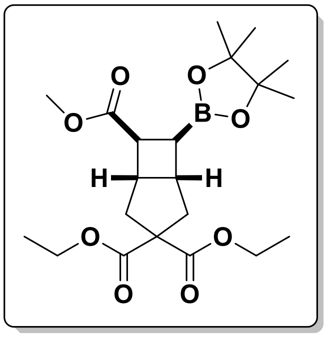

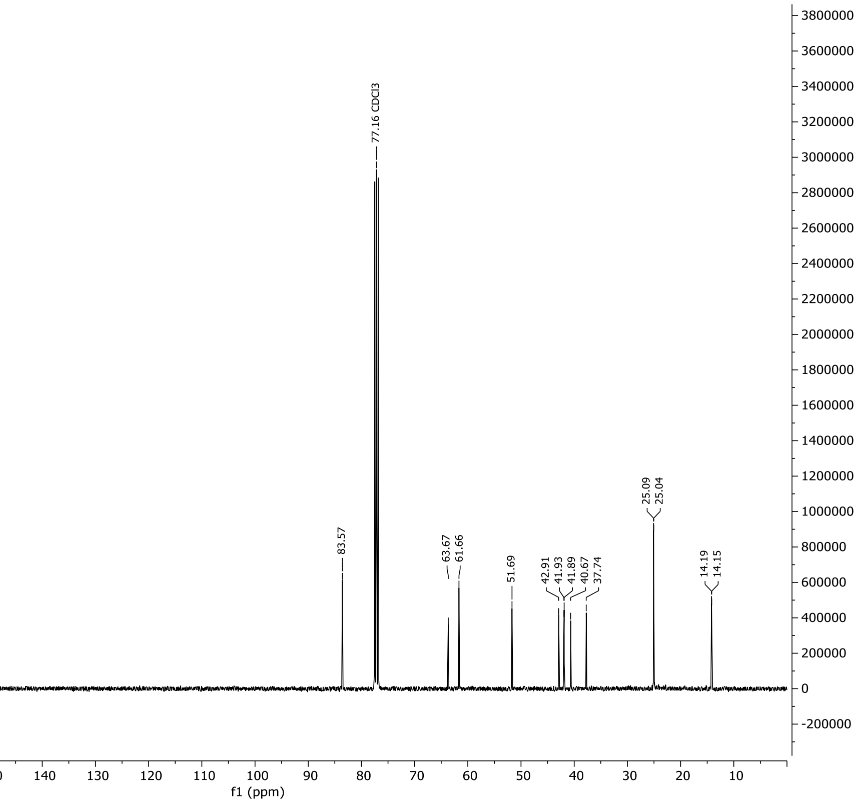

NOESY of 4c:

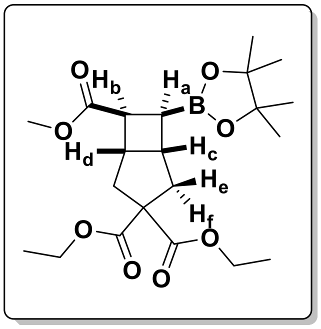

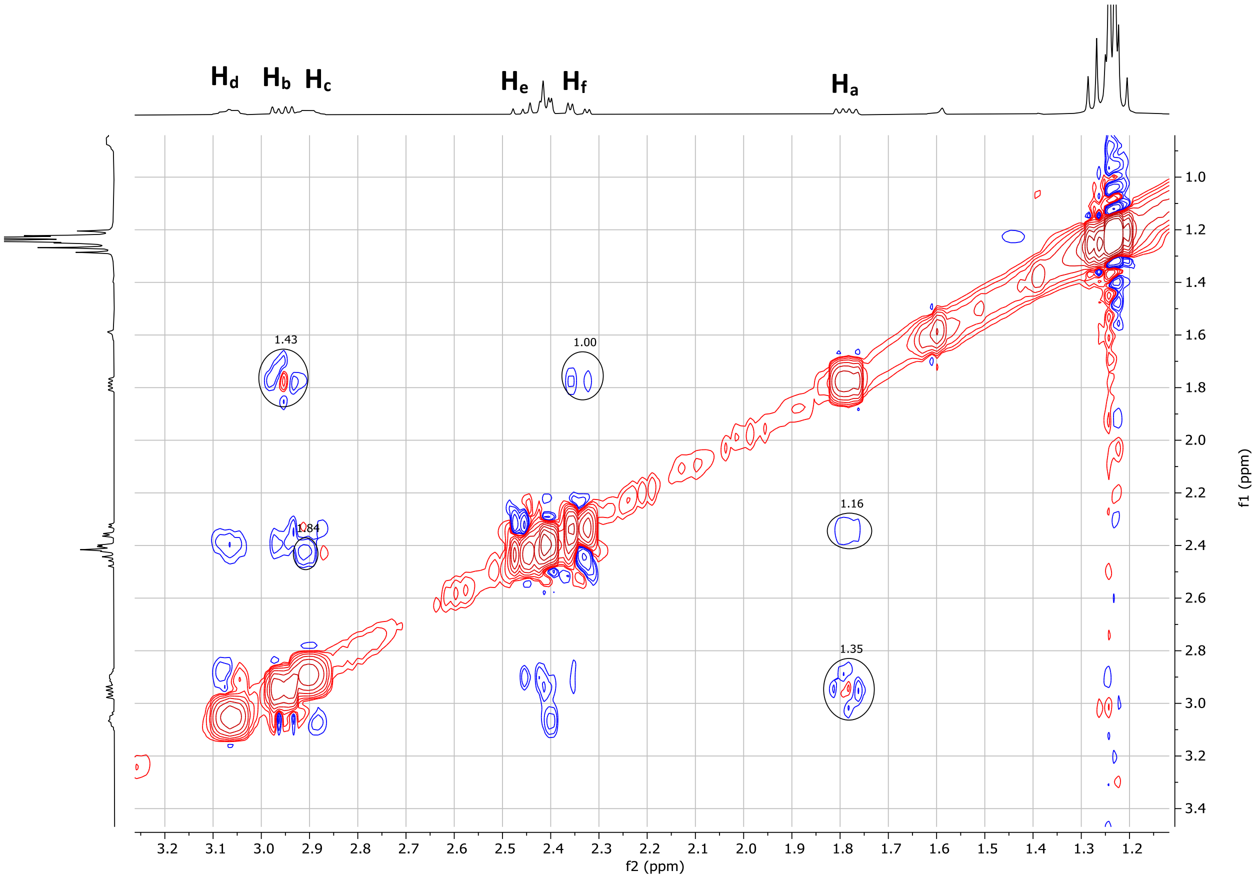

H$_a$ shows nOe with **H$_f$** and **H$_b$**, and no nOe with **H$_c$**, **H$_d$** or **H$_e$**.

H$_b$ shows nOe with **H$_a$**, and no nOe with **H$_c$** and **H$_d$**.

H$_e$ shows nOe with **H$_c$**, and no nOe with **H$_a$**, or **H$_b$**.

H$_f$ shows no nOe with **H$_c$** or **H$_d$**.

^{1}H NMR of 4d (400 MHz, $CDCl_3$)

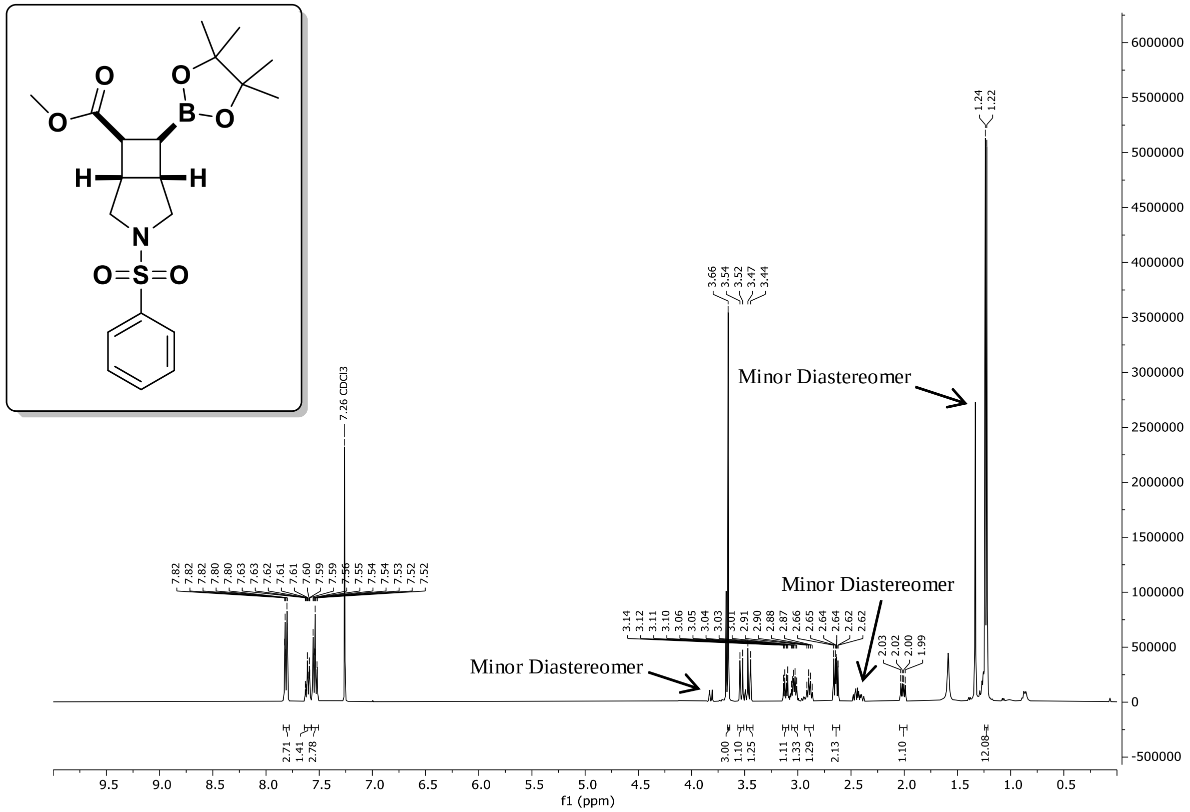

^{13}C NMR of 4d (100 MHz, $CDCl_3$)

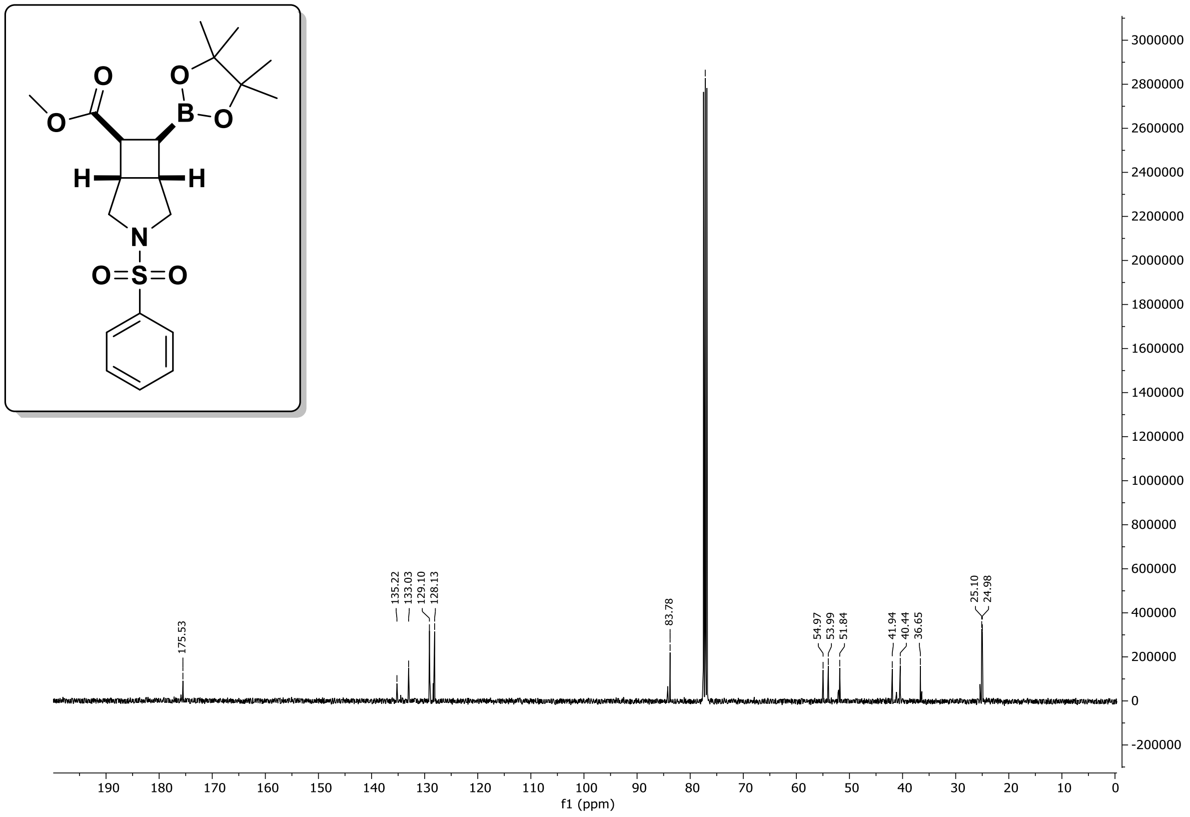

NOESY of 4d:

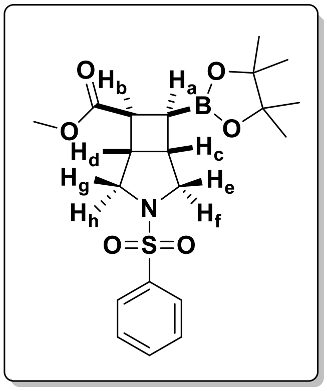

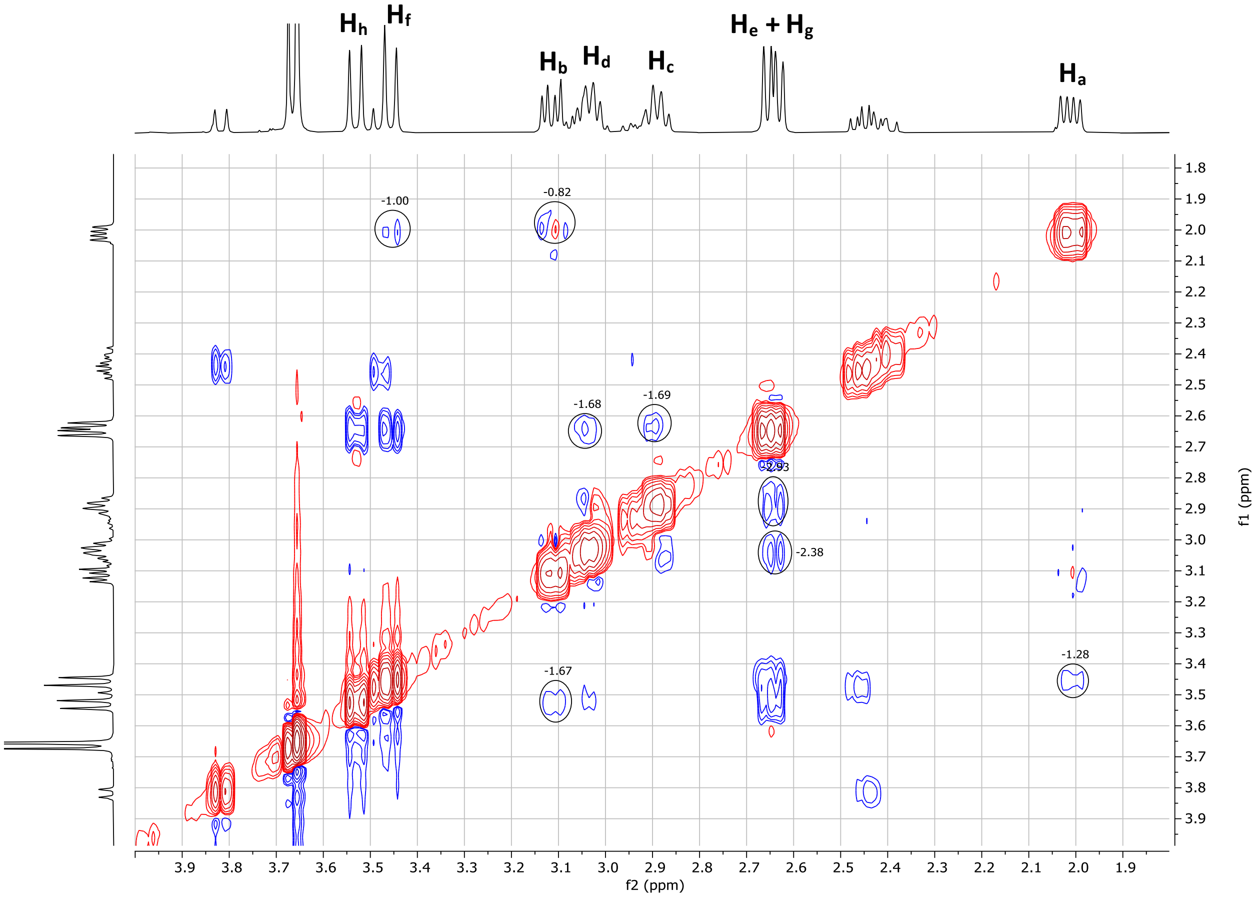

H_a shows nOe with **H_f** and **H_b**. **H_a** shows no nOe with **H_c**, **H_d**, **H_e**, or **H_g**.

H_b shows nOe with **H_a** and **H_h**, and no nOe with **H_c**, **H_d**, **H_e**, or **H_g**.

H_e and **H_g** show nOe with **H_c** and **H_d**, and no nOe with **H_a** or **H_b**.

H_f shows nOe with **H_e**, **H_g**, and **H_a**, and no nOe with **H_b**, **H_c** or **H_d**.

H_h shows nOe with **H_e**, **H_g**, and **H_b**, and no nOe with **H_c**, **H_d**, **H_a**.

^{1}H NMR of 4e (400 MHz, $CDCl_3$)

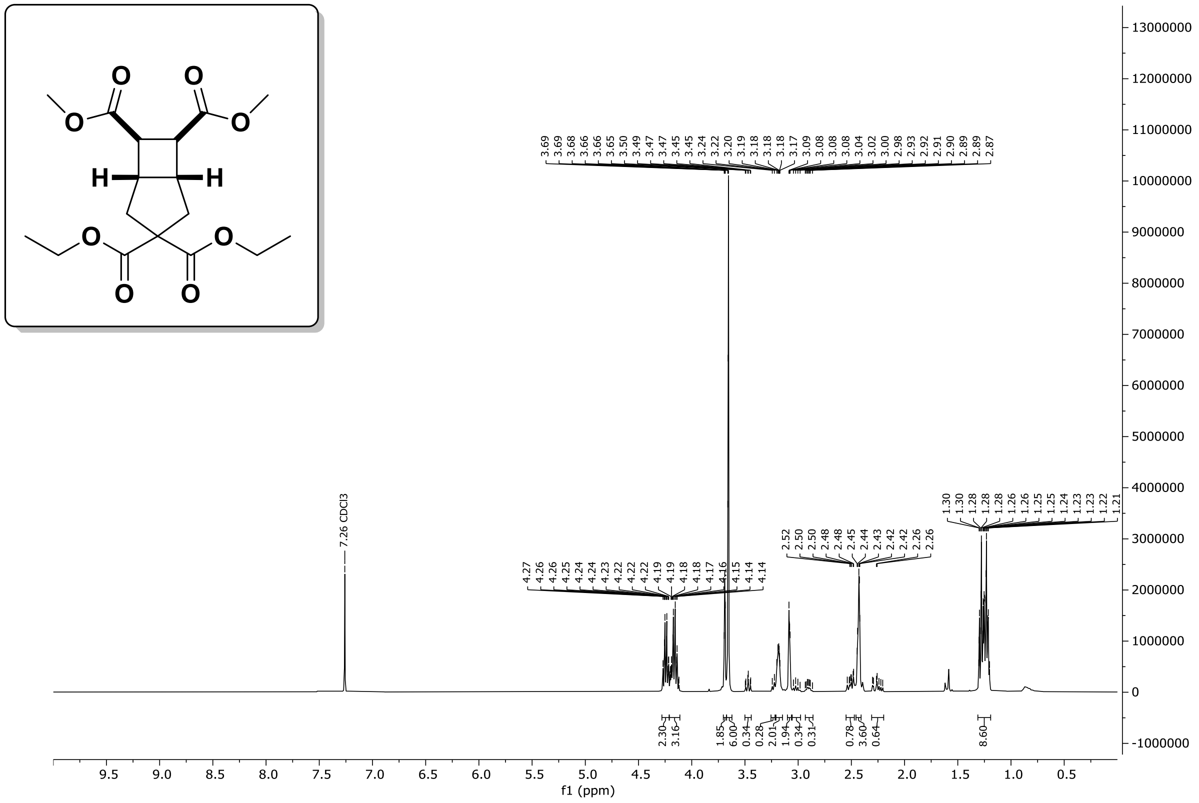

^{13}C NMR of 4e (100 MHz, $CDCl_3$)

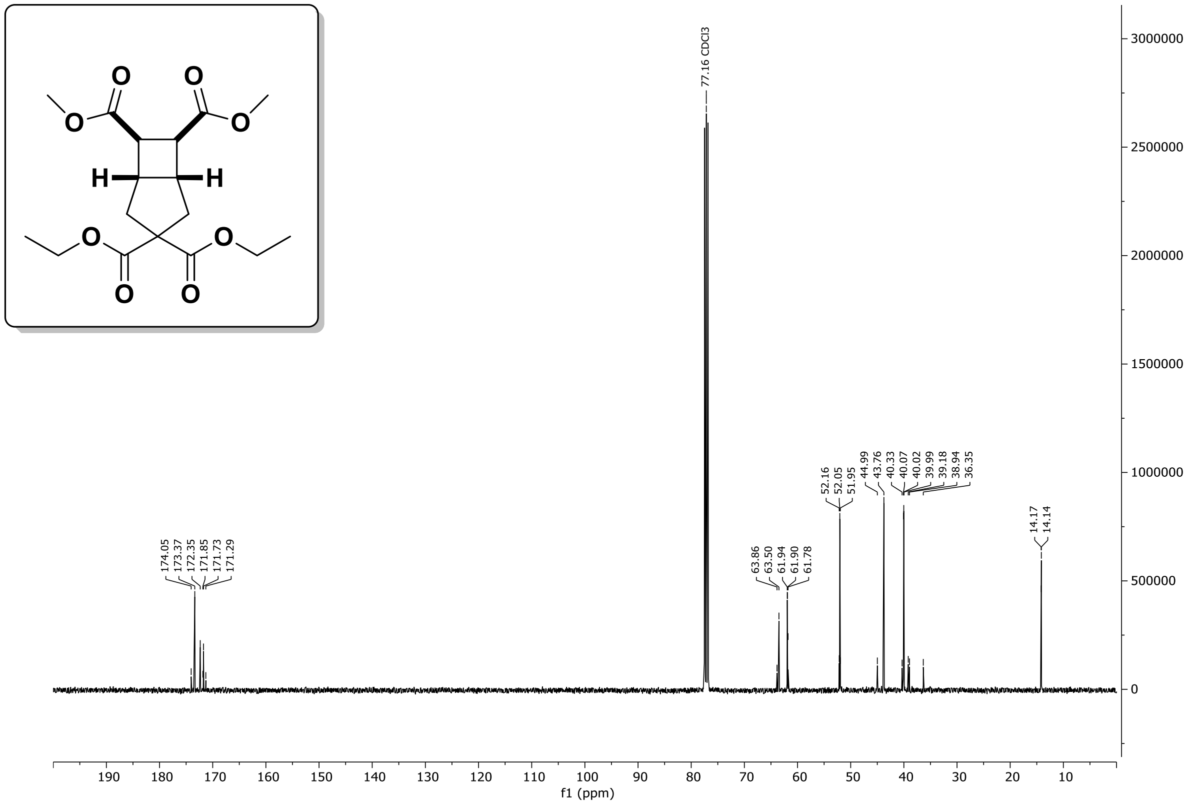

^{1}H NMR of S17 (400 MHz, $CDCl_3$)

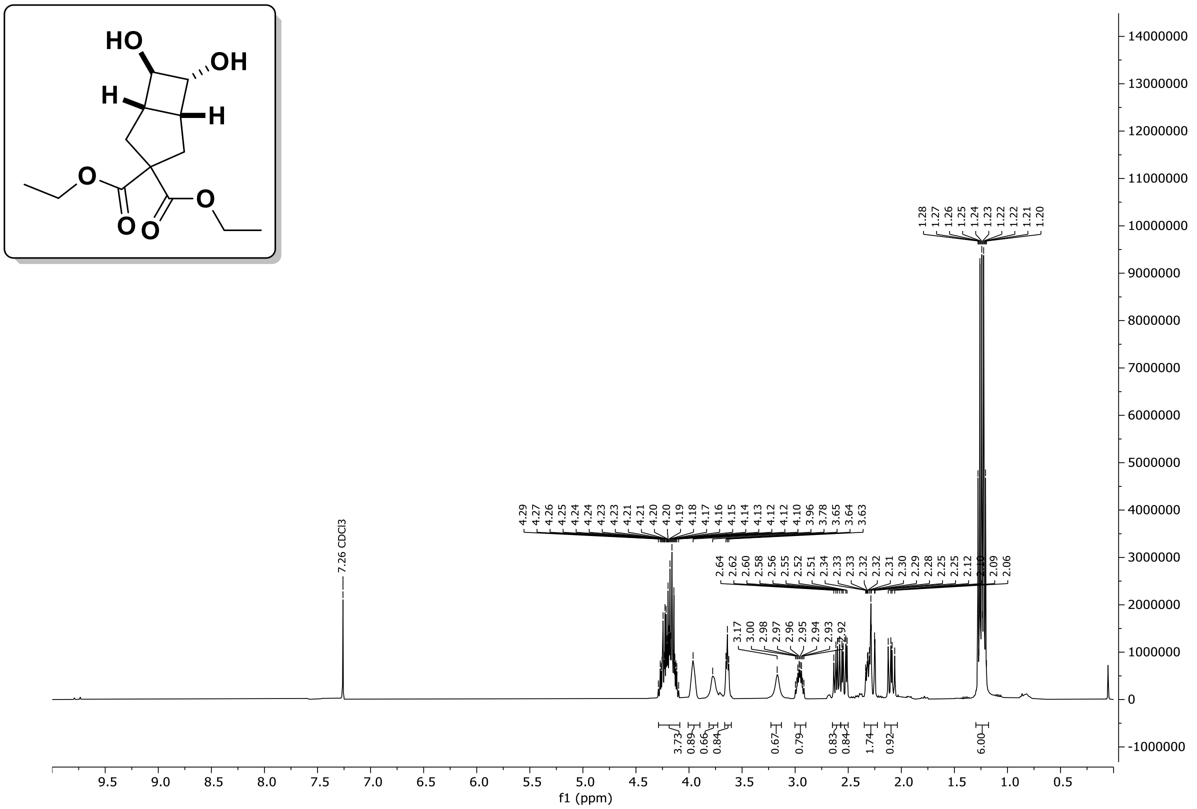

^{13}C NMR of S17 (100 MHz, $CDCl_3$)

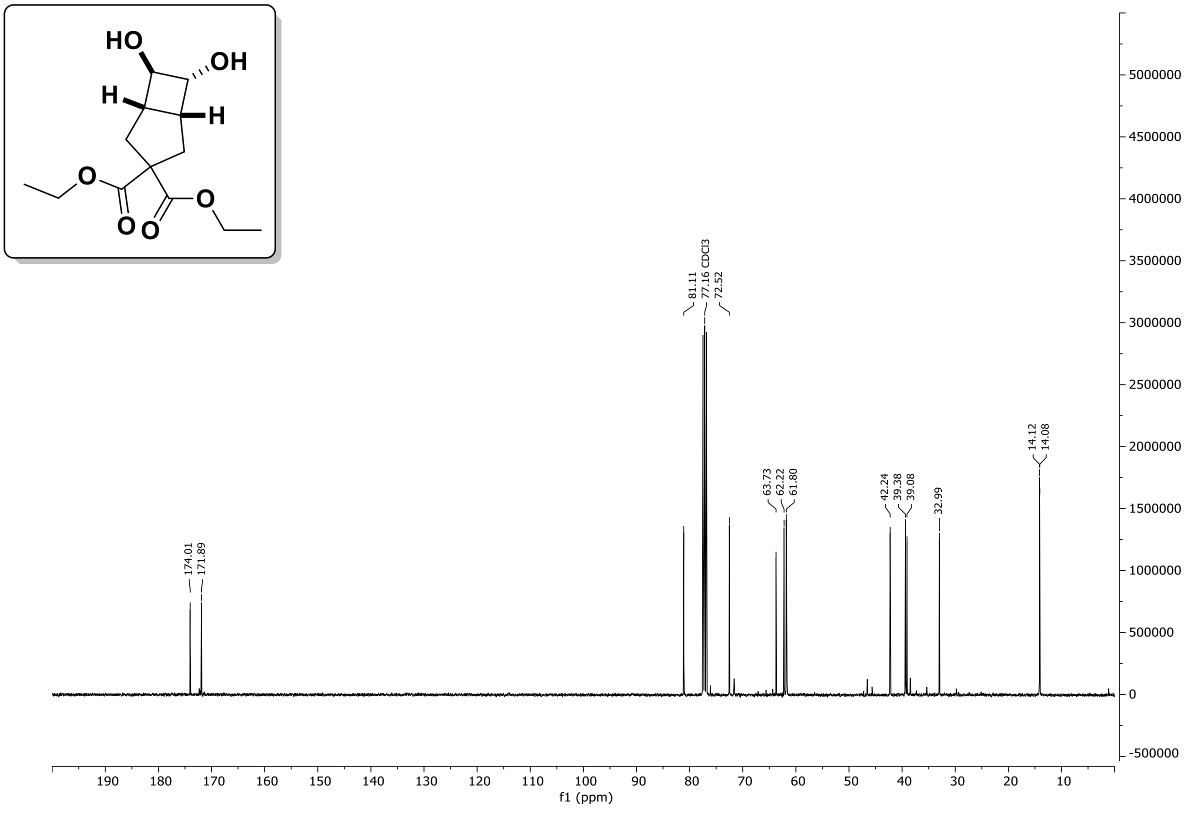

NOESY of S17:

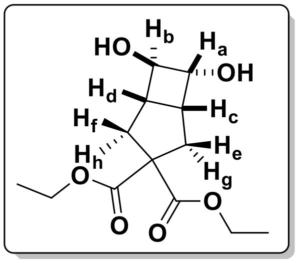

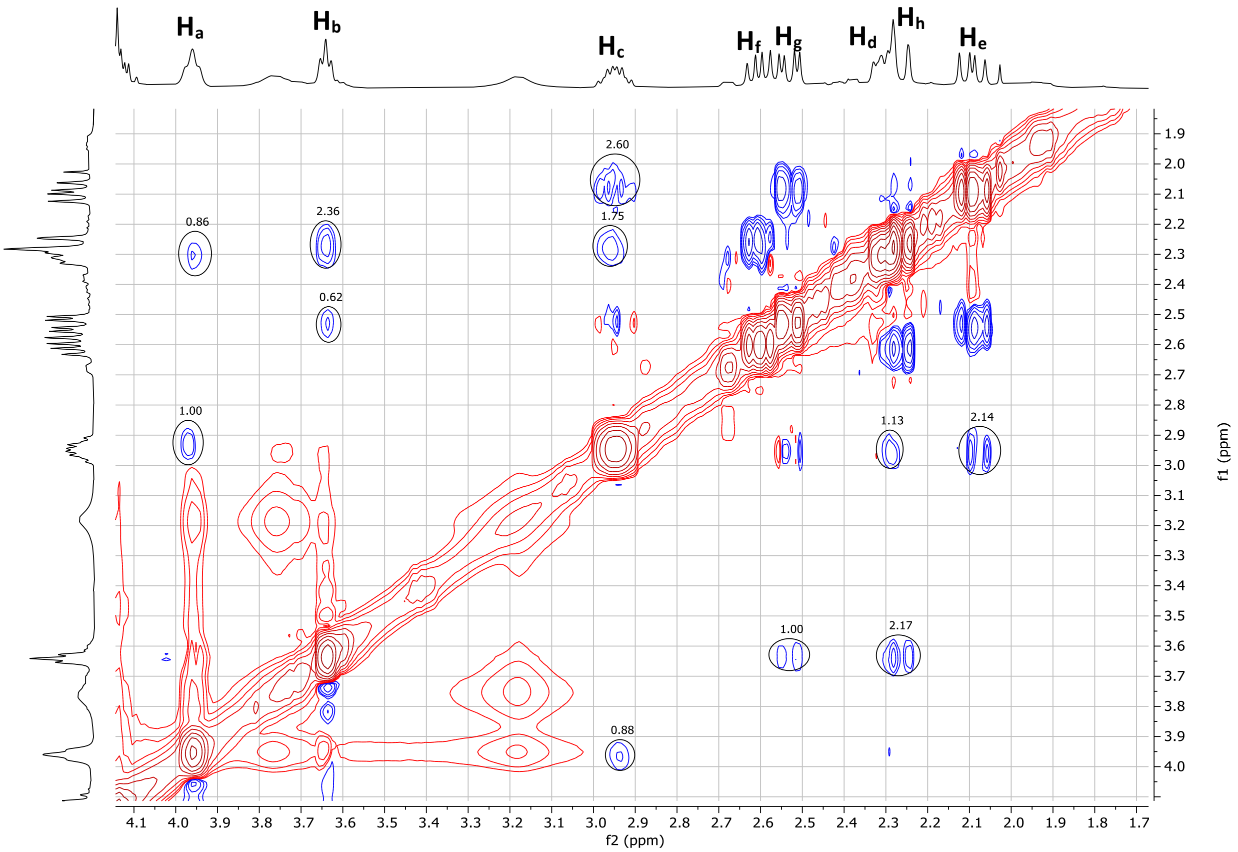

Ha shows nOe with **Hc** and **Hd**, and no nOe with **Hg** or **Hh**.

Hb shows nOe with **Hh** and **Hg**, and no nOe with **Hc** or **Hd**.

Hc shows nOe with **Ha**, **Hd**, and **He**, and no nOe with **Hb**.

^{1}H NMR of S18-*anti* (400 MHz, MeOD)

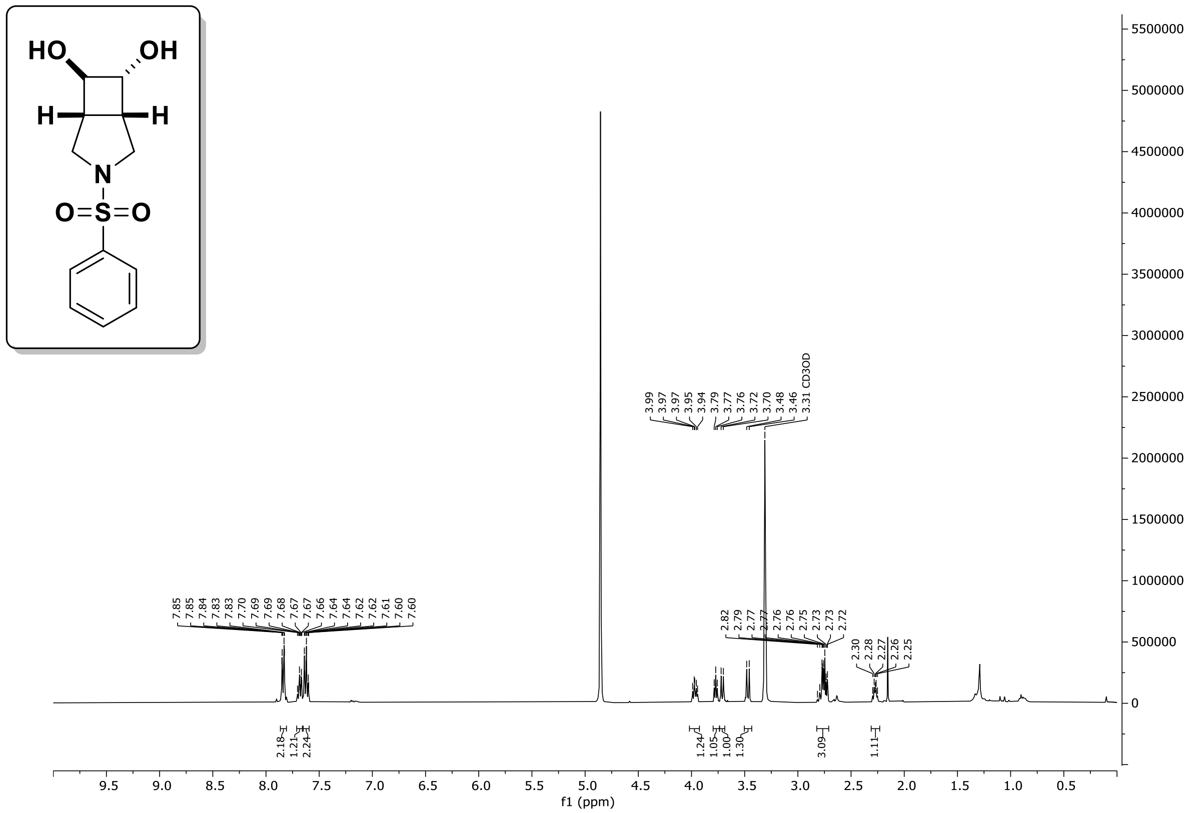

^{13}C NMR of S18-*anti* (100 MHz, MeOD)

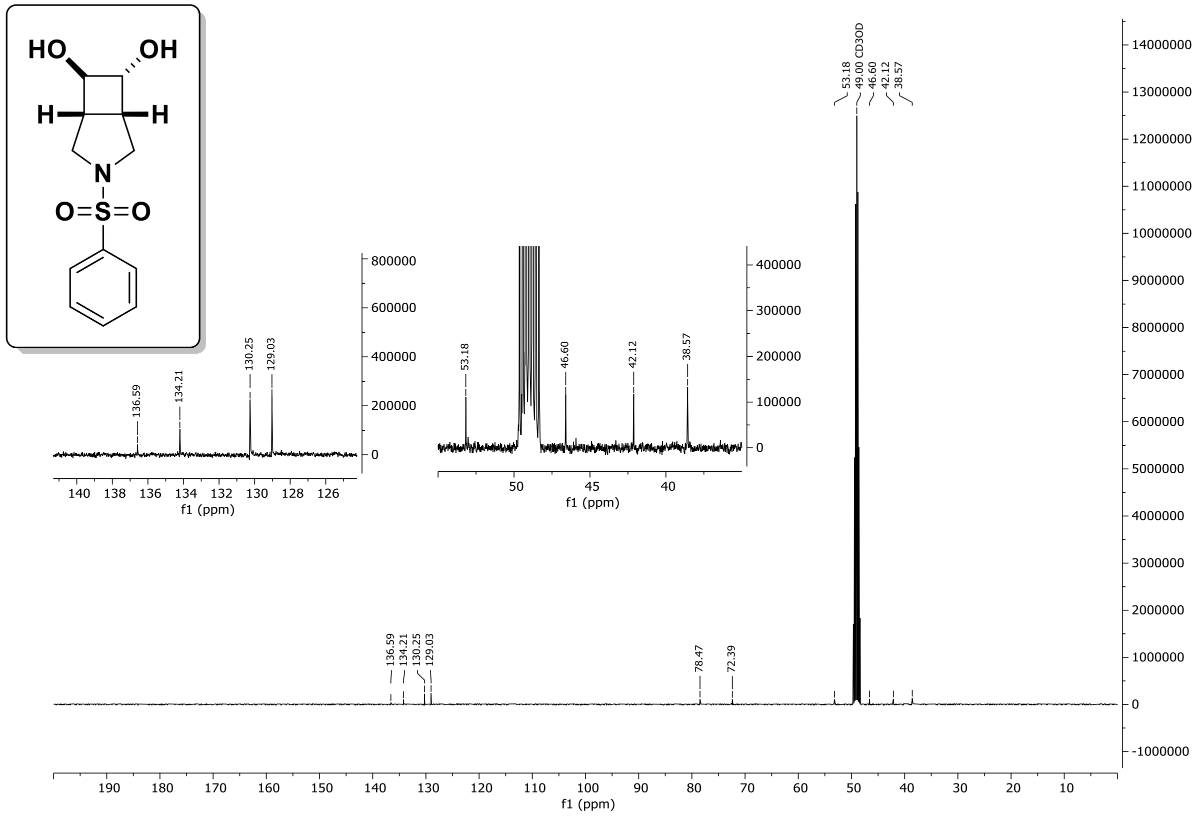

NOESY of S18-*anti*:

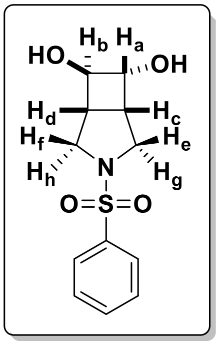

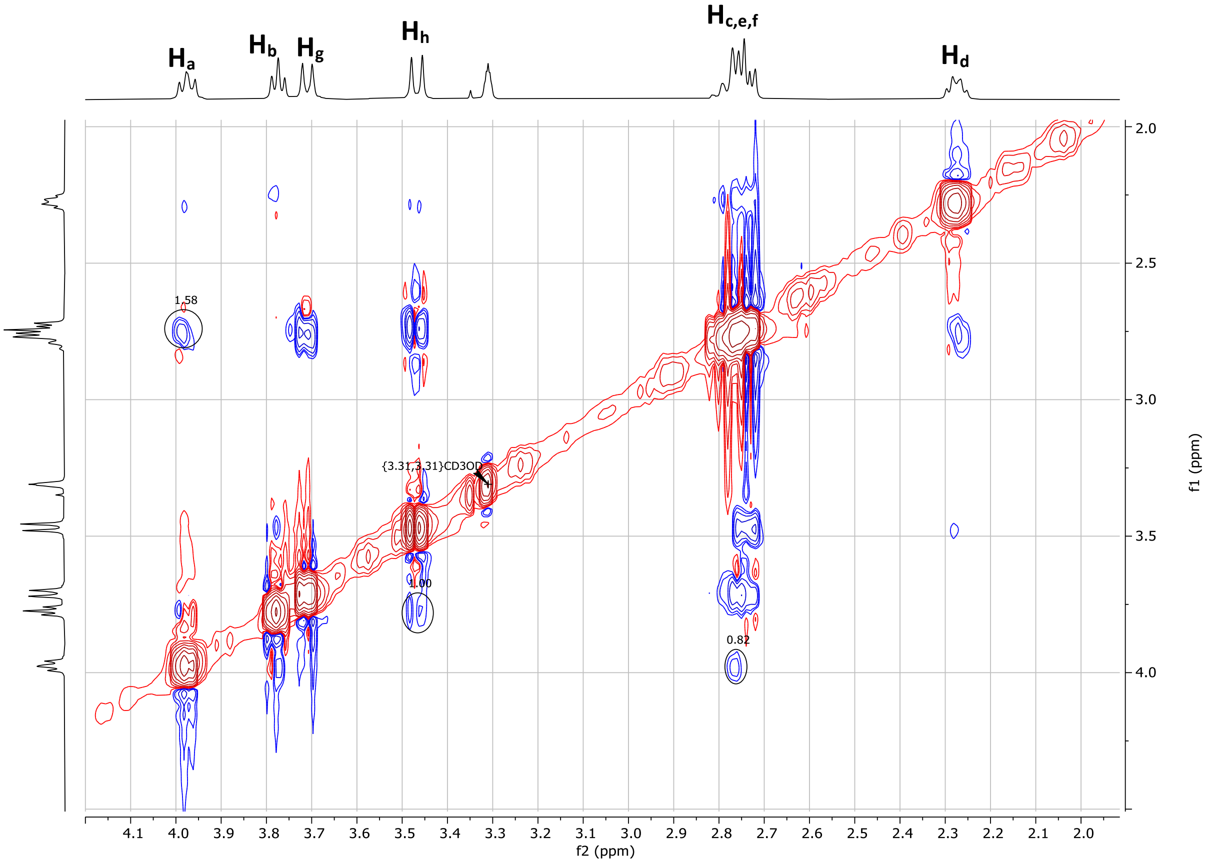

H_a shows nOe with one or more of **H_c**, **H_e**, or **H_f**, and no nOe with **H_g** or **H_h**.

H_b shows nOe with **H_h**, and no nOe with **H_c**, **H_e** or **H_f**.

^{1}H NMR of S18-*syn* (400 MHz, MeOD)

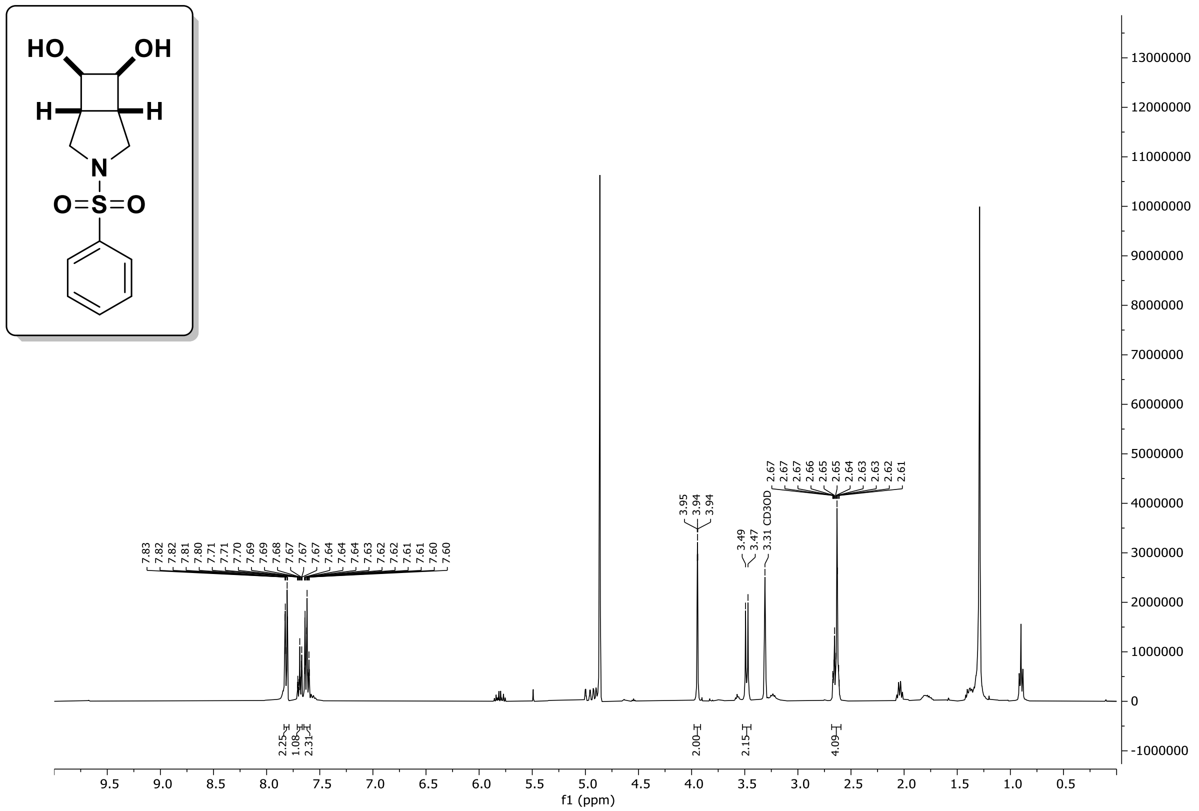

^{13}C NMR of S18-*syn* (100 MHz, MeOD)

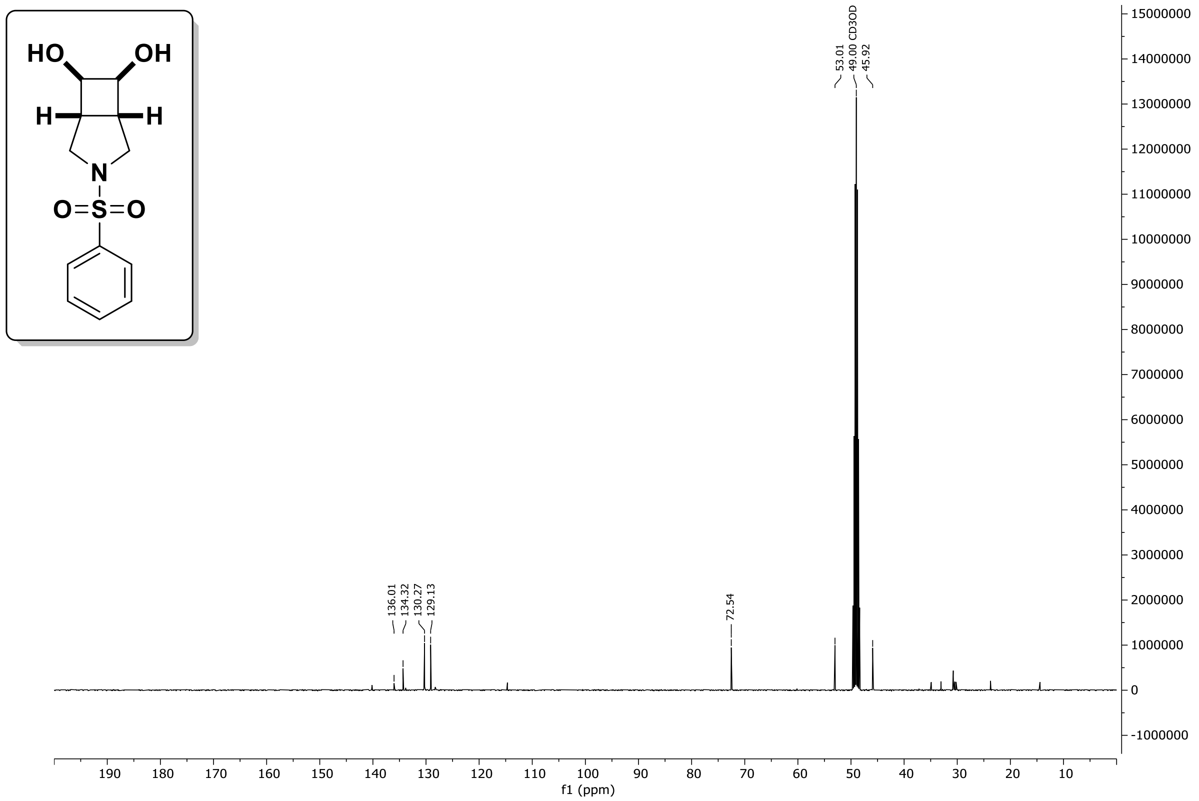

NOESY of S18-*syn*:

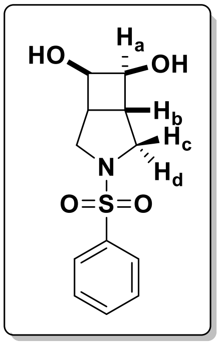

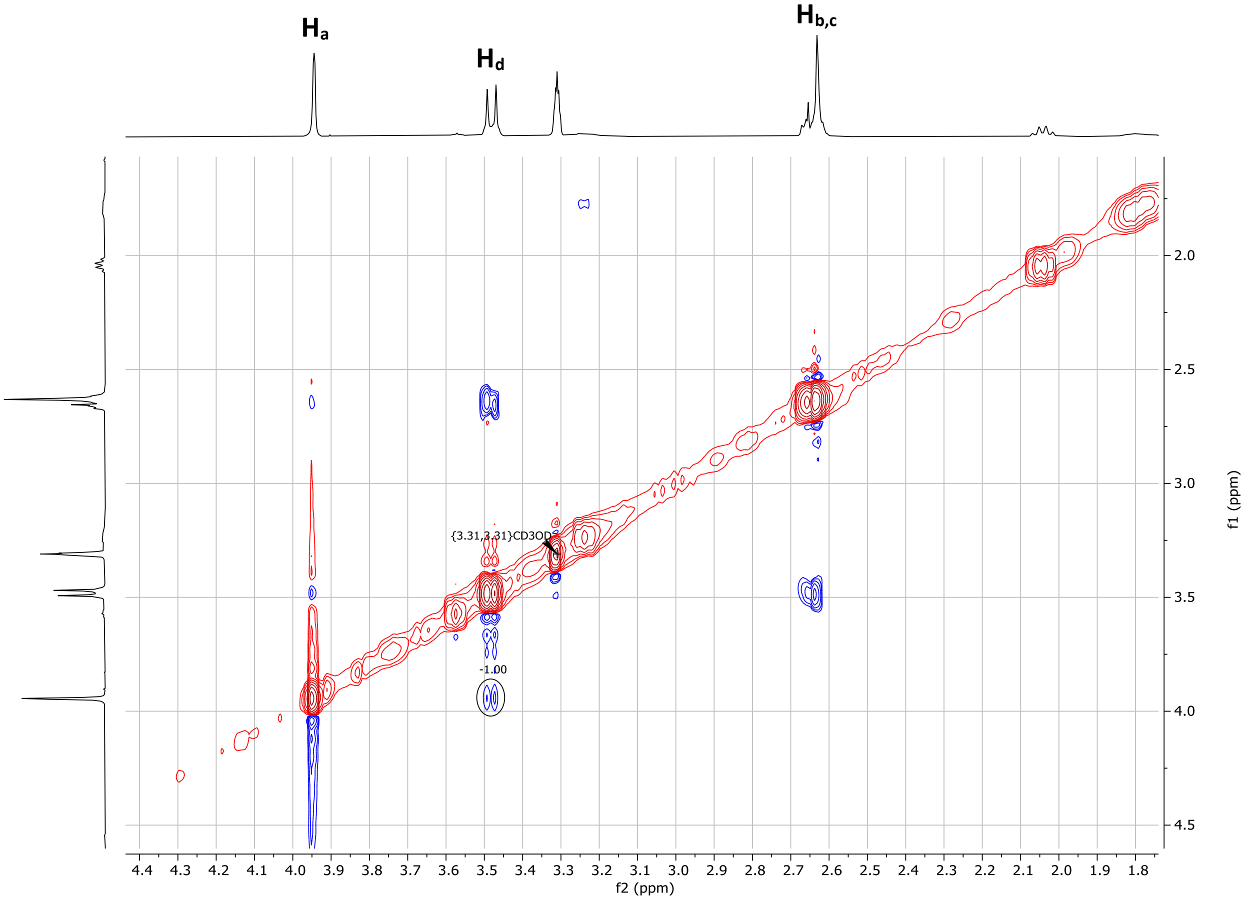

H$_a$ shows nOe with **H**$_d$, and no nOe with **H**$_b$ or **H**$_c$.

^{1}H NMR of 4h (400 MHz, CDCl$_3$)

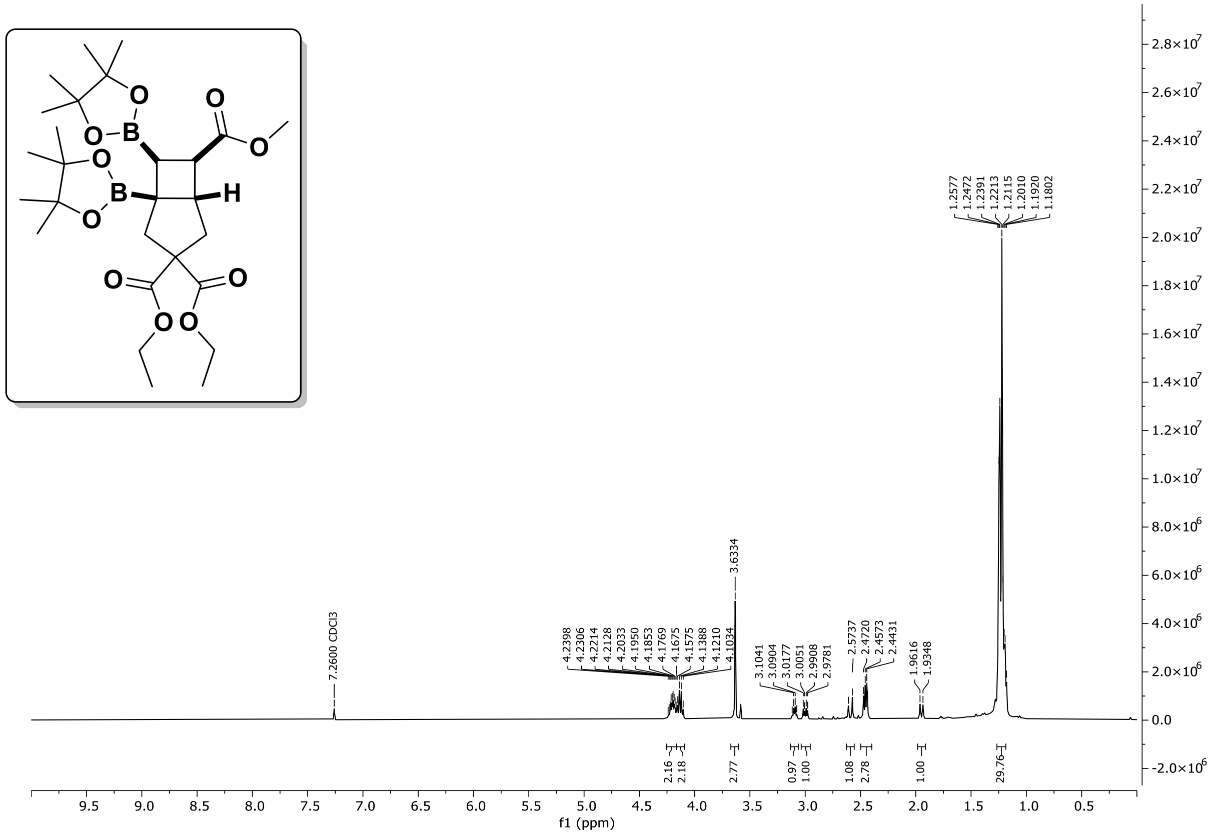

^{13}C NMR of 4h (100 MHz, CDCl$_3$)

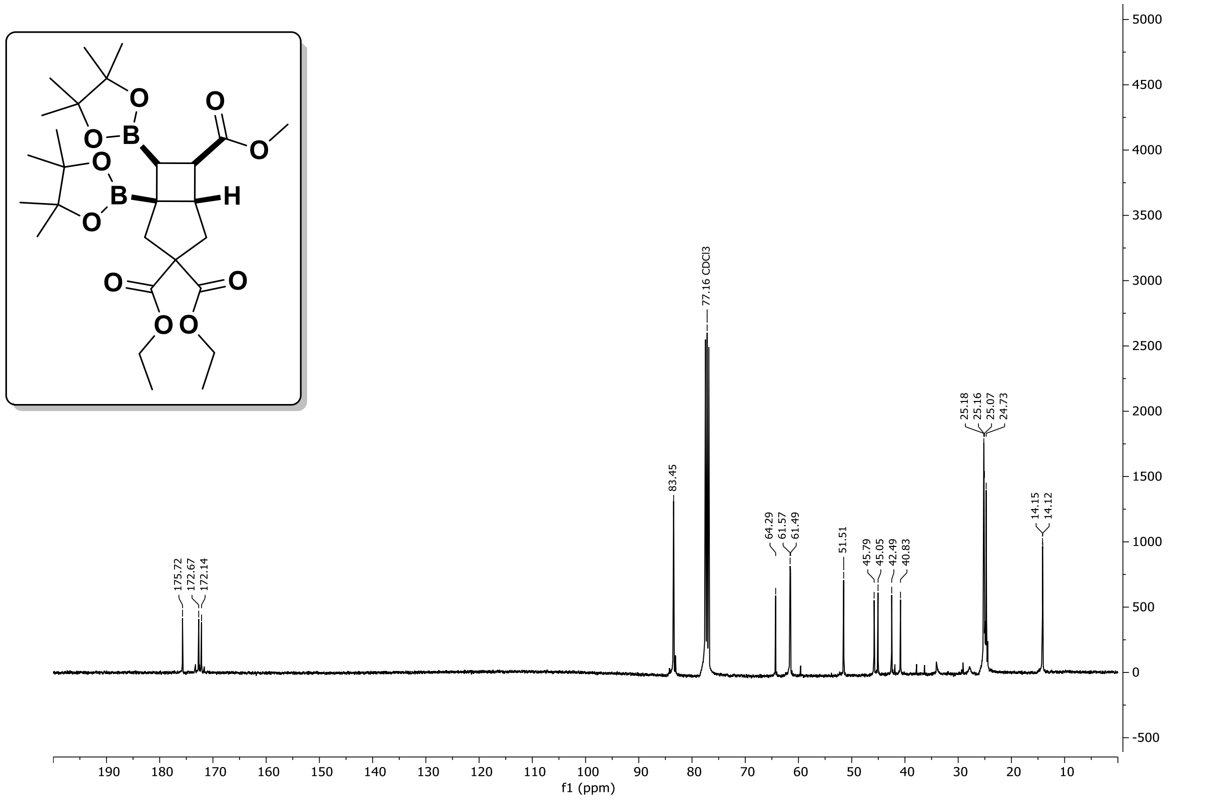

NOESY of 4h:

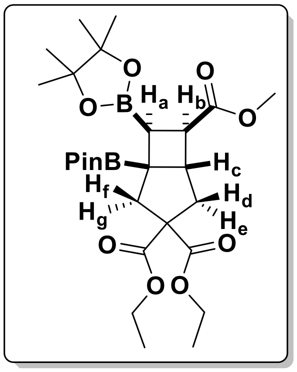

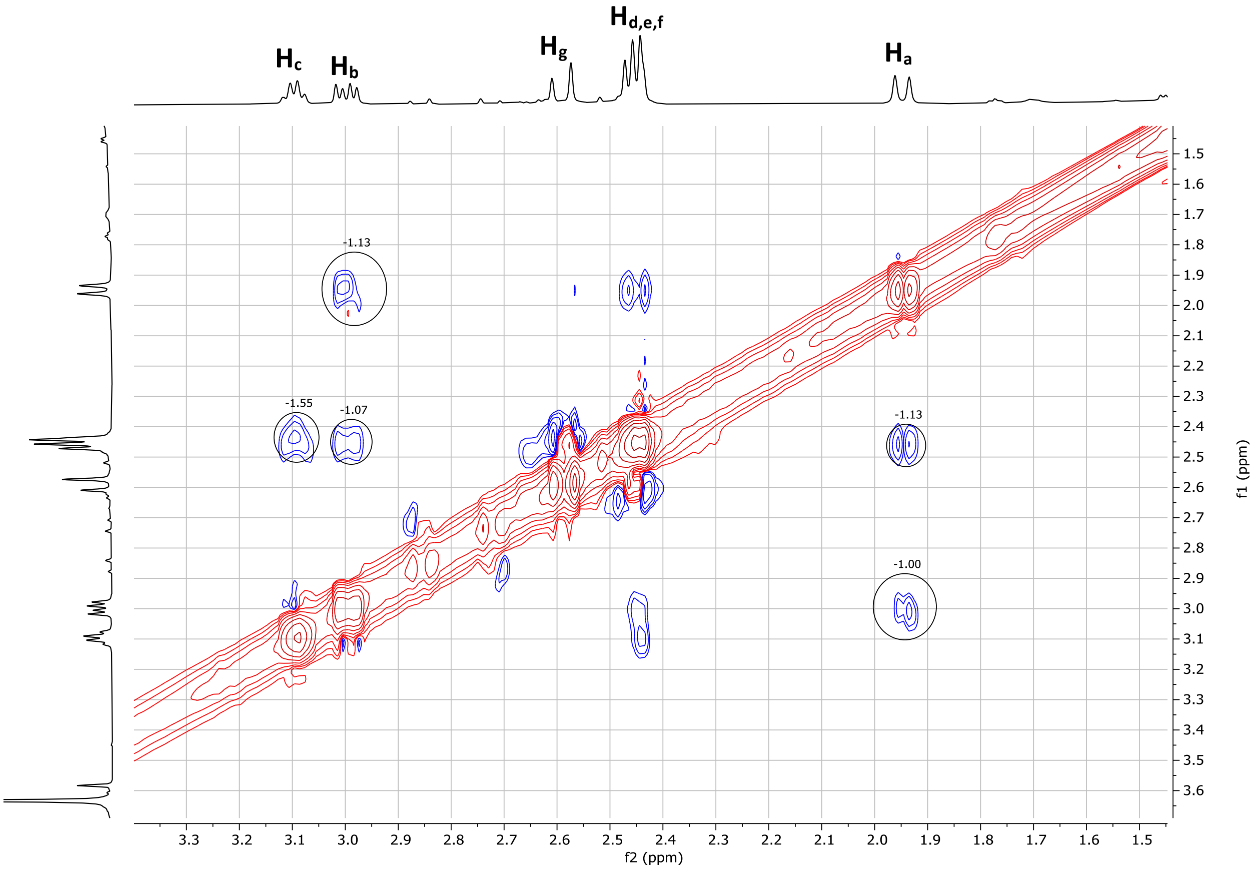

$\mathbf{H_a}$ shows nOe with $\mathbf{H_b}$, and no nOe with $\mathbf{H_c}$.

$\mathbf{H_b}$ shows nOe with $\mathbf{H_a}$, and no nOe with $\mathbf{H_c}$.

^{1}H NMR of 5 (400 MHz, $CDCl_3$)

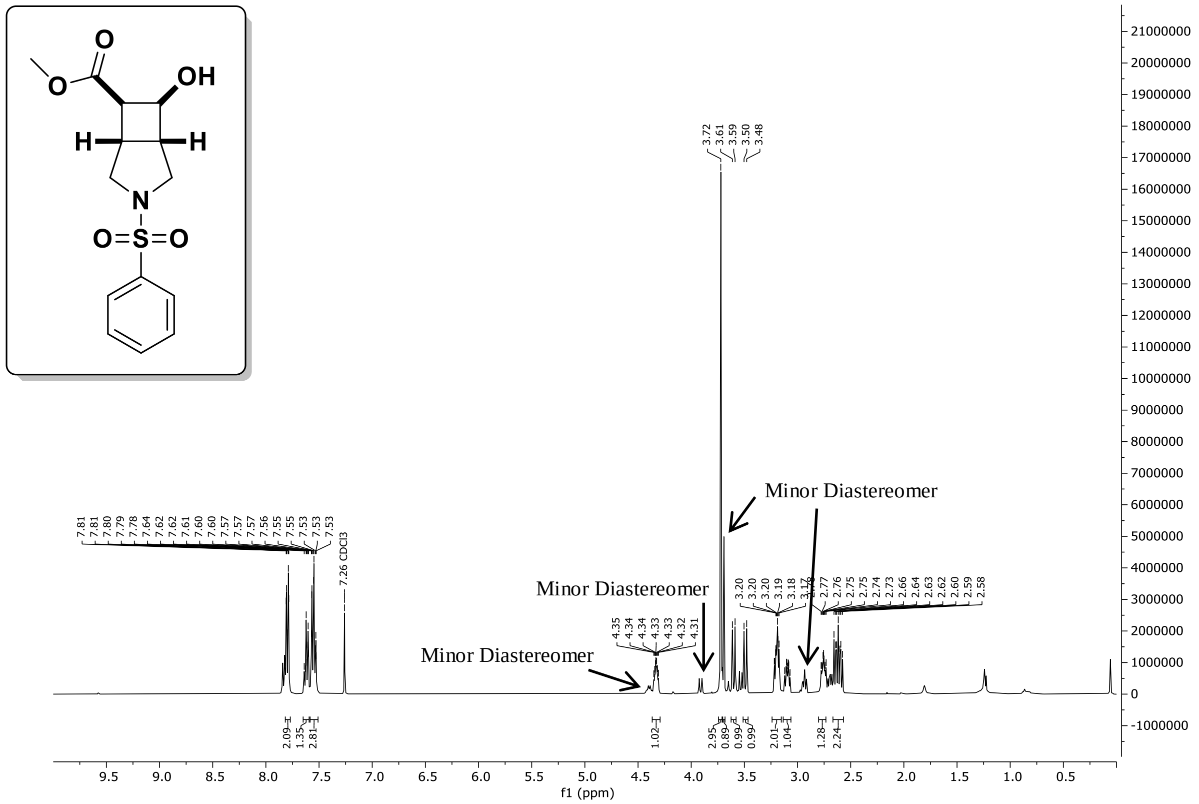

^{13}C NMR of 5 (100 MHz, $CDCl_3$)

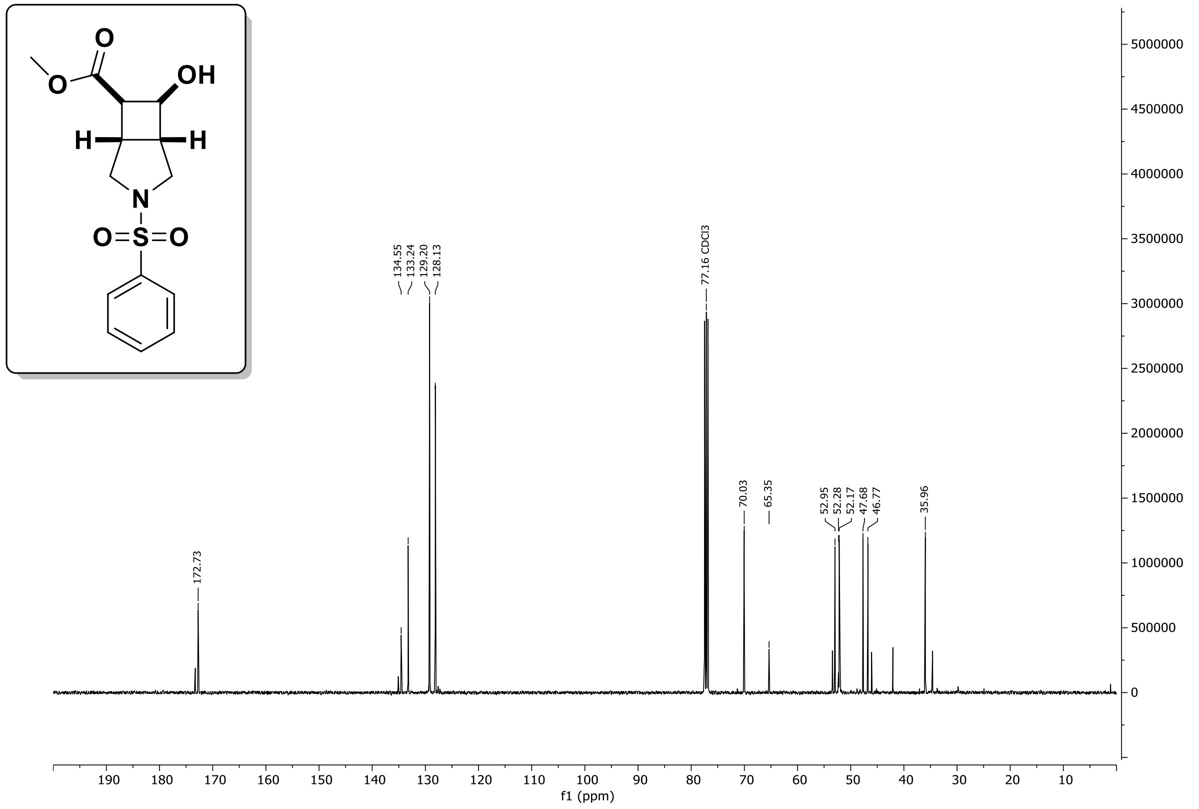

^{1}H NMR of 6 (400 MHz, DMSO-d_6)

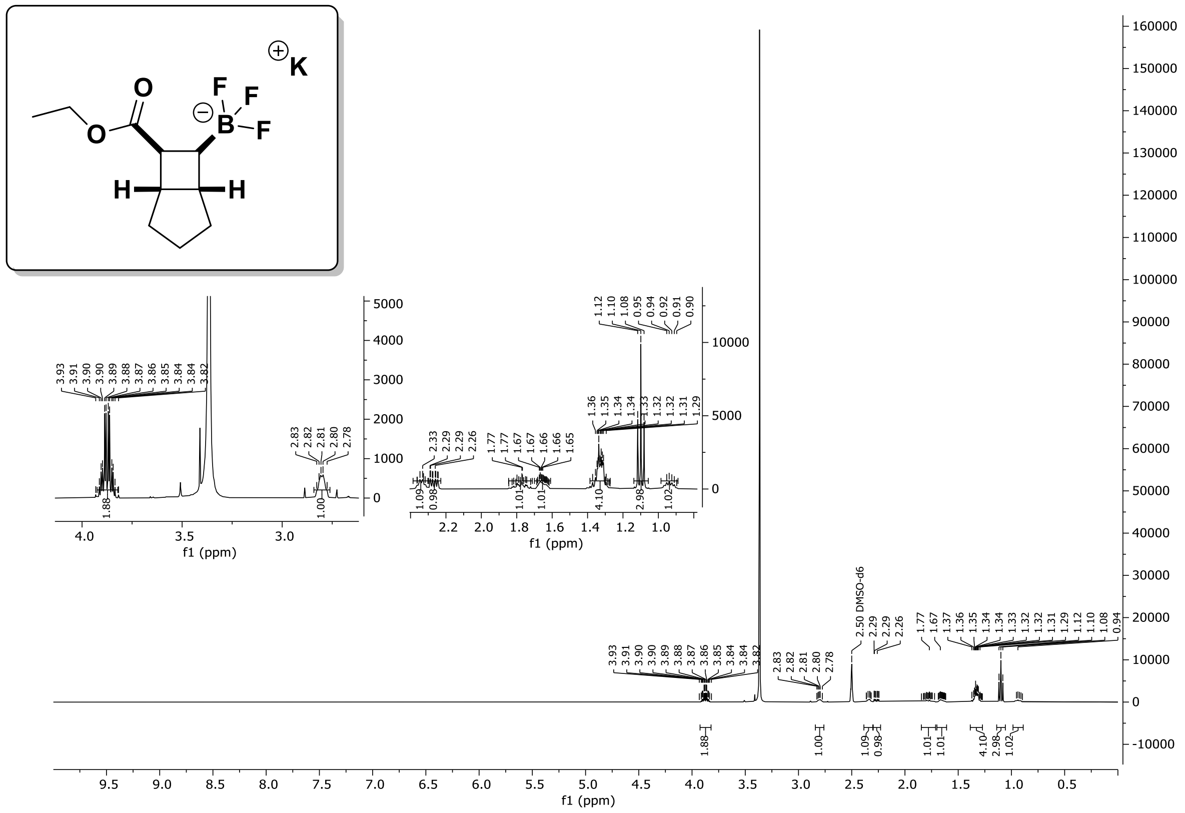

^{13}C NMR of 6 (100 MHz, DMSO-d_6)

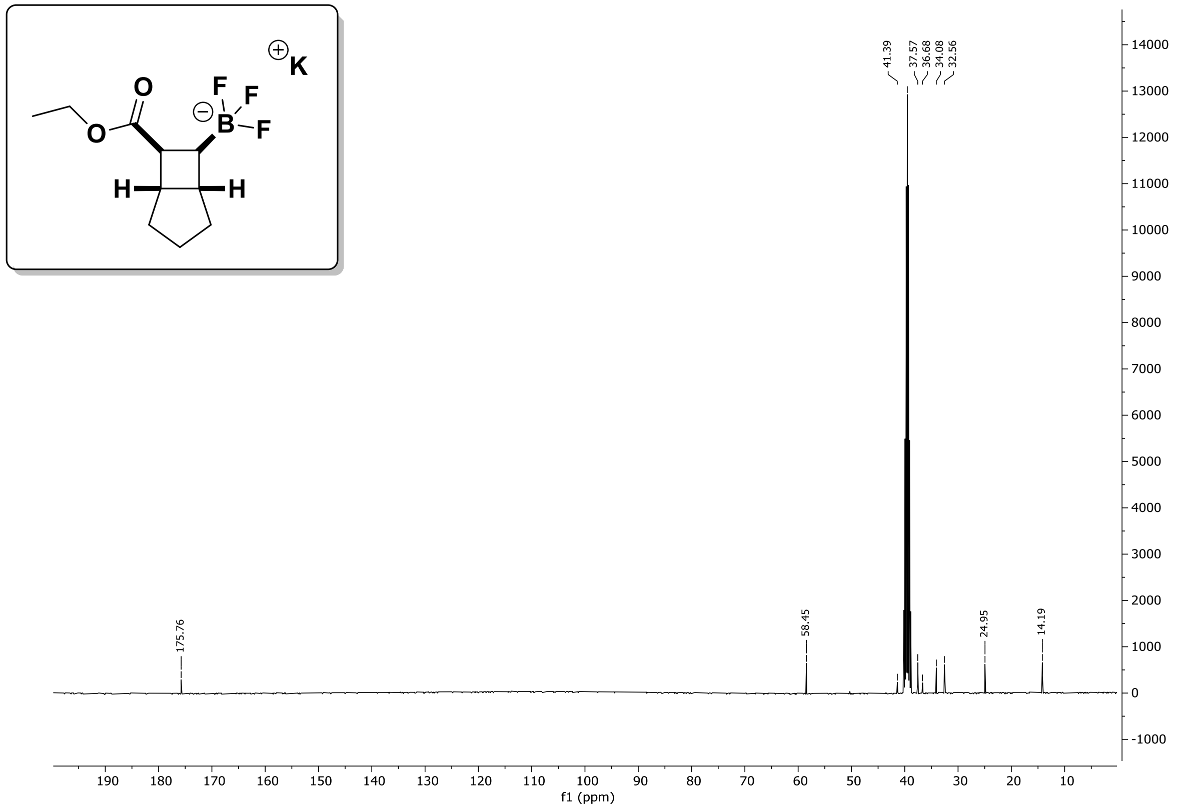

^{19}F NMR of 6 (376 MHz, DMSO-d_6)

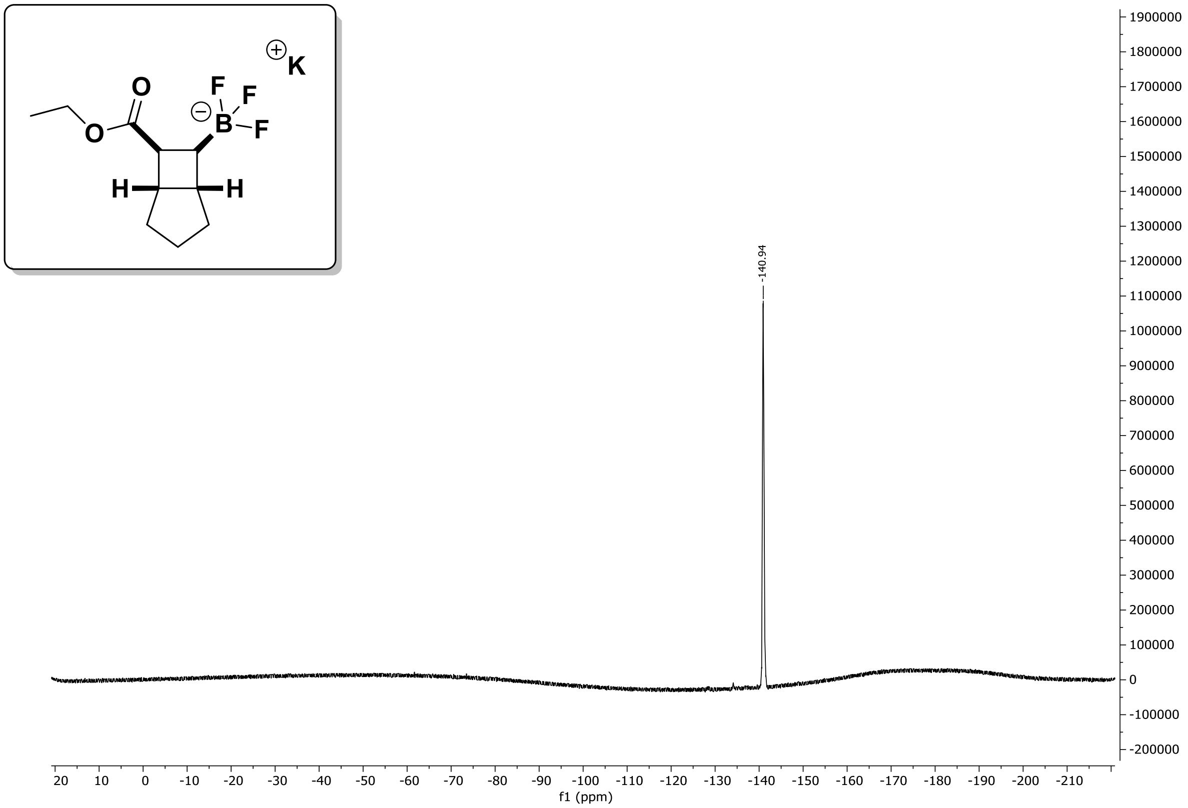

^{1}H NMR of 7 (400 MHz, DMSO-d_6)

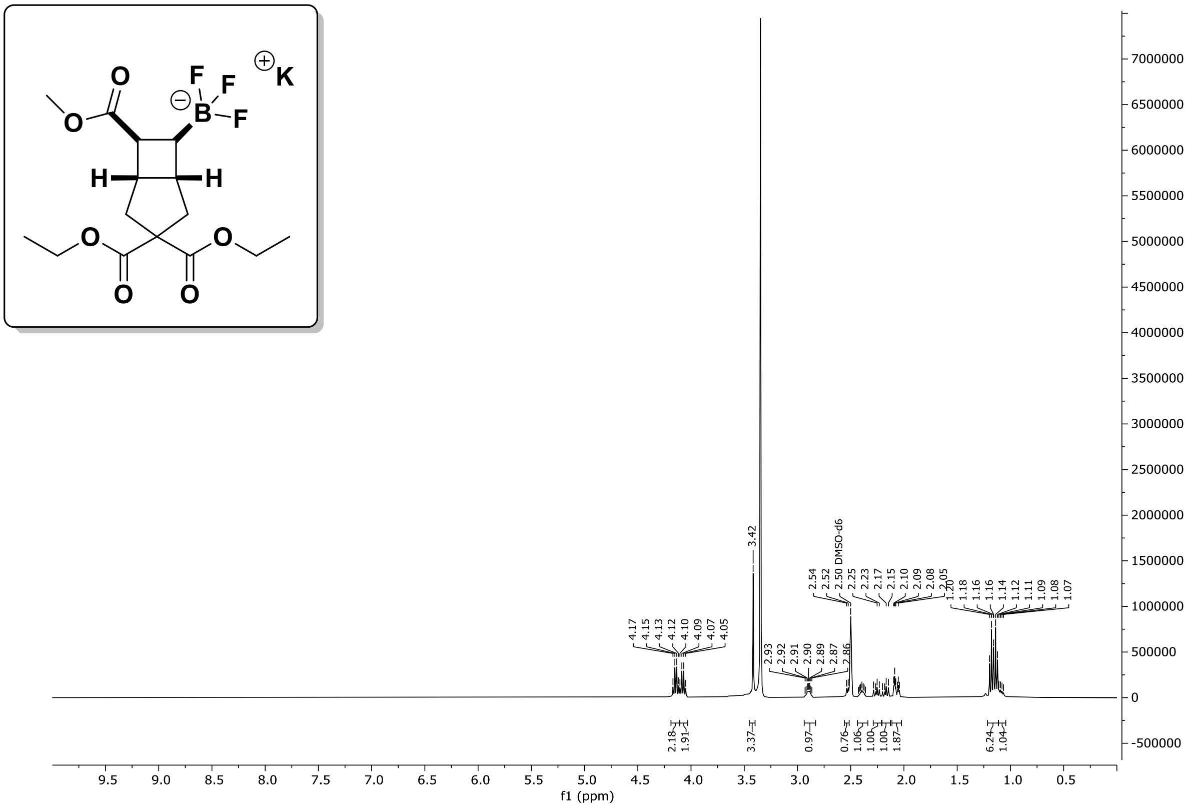

^{13}C NMR of 7 (100 MHz, DMSO-d_6)

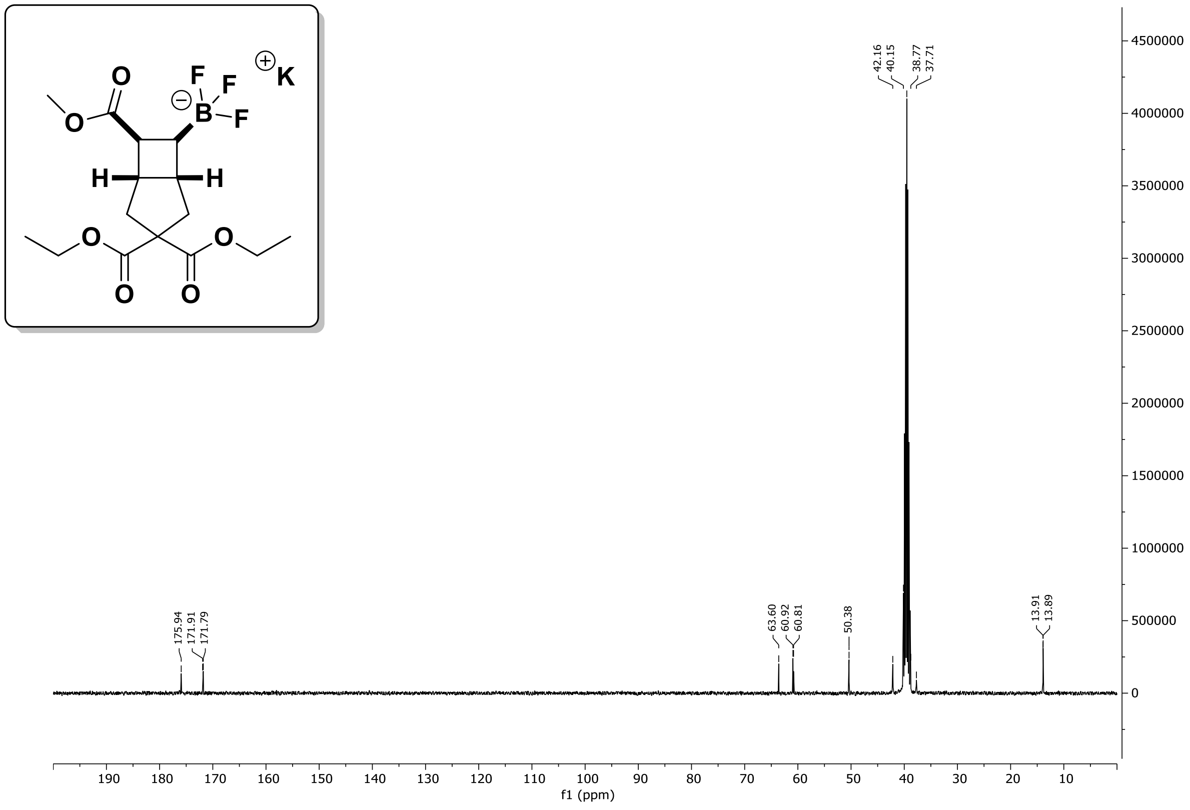

^{19}F NMR of 7 (376 MHz, DMSO-d_6)

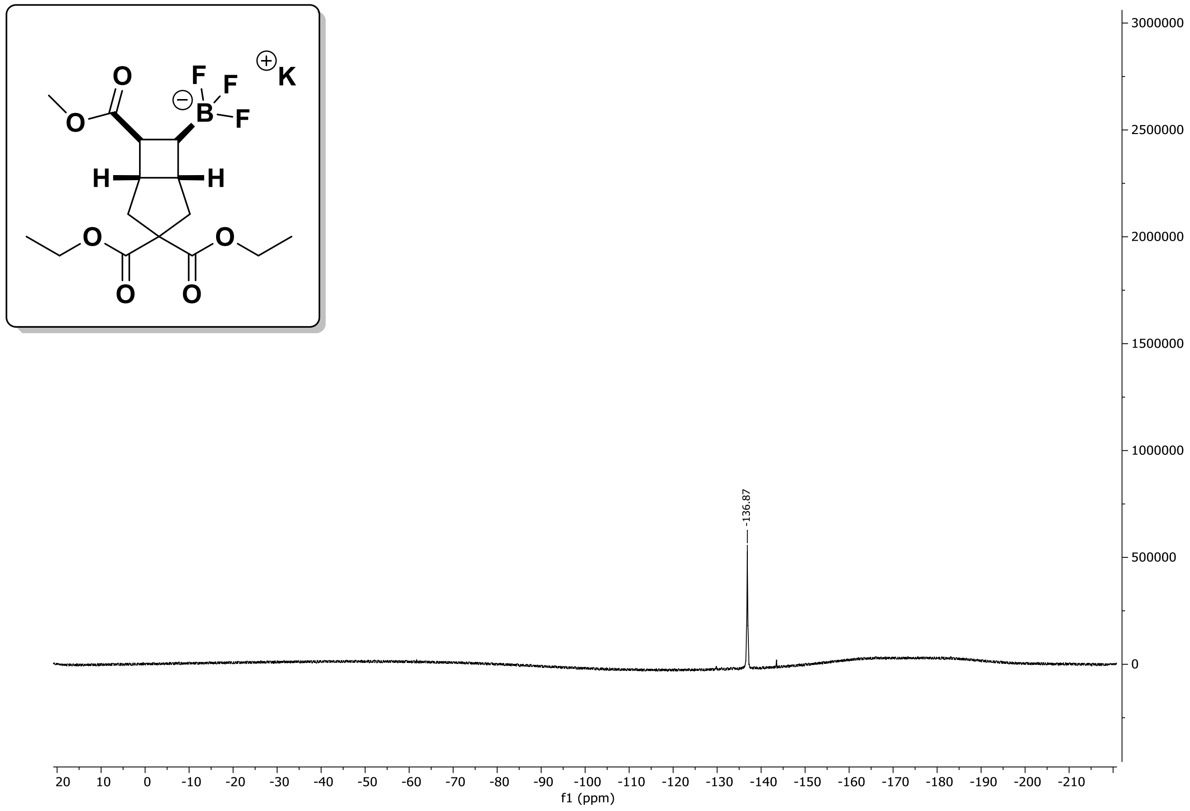

NOESY of 7:

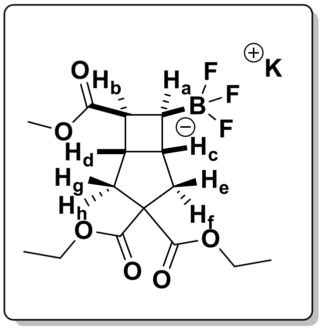

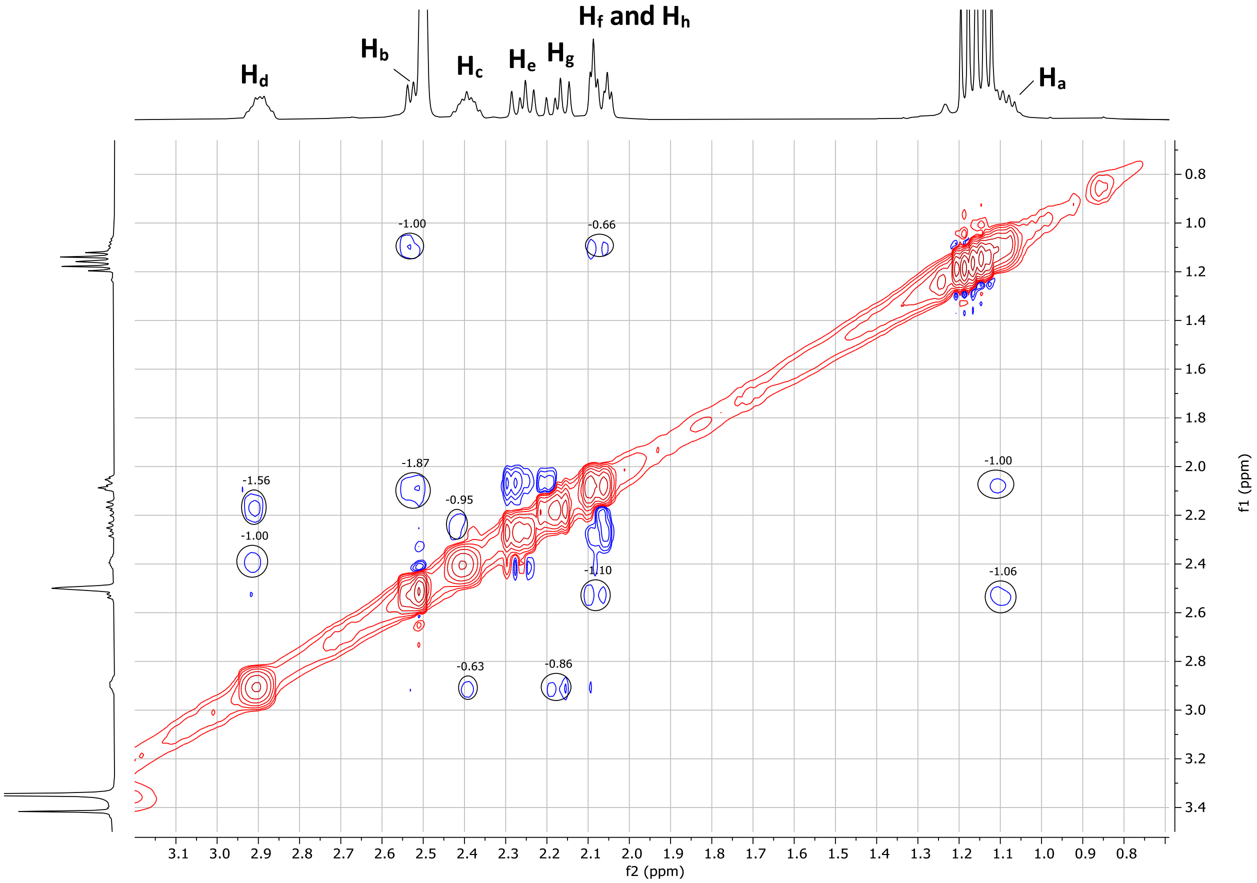

$\mathbf{H_a}$ shows nOe with $\mathbf{H_f}$, $\mathbf{H_h}$, and $\mathbf{H_b}$, and no nOe with $\mathbf{H_c}$, $\mathbf{H_d}$, $\mathbf{H_e}$, $\mathbf{H_g}$.

$\mathbf{H_b}$ shows nOe with $\mathbf{H_a}$, $\mathbf{H_f}$, and $\mathbf{H_h}$, and no nOe with $\mathbf{H_c}$, $\mathbf{H_d}$, $\mathbf{H_e}$, $\mathbf{H_g}$.

$\mathbf{H_c}$ shows nOe with $\mathbf{H_d}$ and $\mathbf{H_e}$, and no nOe with $\mathbf{H_a}$, $\mathbf{H_b}$, $\mathbf{H_f}$, and $\mathbf{H_h}$.

$\mathbf{H_d}$ shows nOe with $\mathbf{H_c}$ and $\mathbf{H_g}$, and no nOe with $\mathbf{H_a}$, $\mathbf{H_b}$, $\mathbf{H_f}$, and $\mathbf{H_h}$.
